# Prevalence of depression, anxiety, and stress among university students in the post-COVID-19 period: a systematic review and meta-analysis

**DOI:** 10.1186/s40359-026-05066-4

**Published:** 2026-06-29

**Authors:** Josefa A. Antón-Ruiz, Miguel Bernabé, Esther Heredia-Oliva

**Affiliations:** 1 Universidad de Alicante (UA), Alicante, Spain; 2 Universidad Nacional de Educación a Distancia (UNED), Madrid, Spain

**Keywords:** Depression, Anxiety, Stress, University students, Post-COVID-19

## Abstract

**Background:**

The COVID-19 pandemic has had lasting effects on university students’ mental health. While numerous studies have examined depression, anxiety, and stress during the pandemic, the post-pandemic burden of these conditions remains unclear. This systematic review and meta-analysis aimed to estimate the pooled prevalence of depression, anxiety, and stress among university students during the post-COVID-19 period.

**Methods:**

A systematic search of PubMed, Web of Science, MEDLINE, and Scopus was conducted from inception to August 2025 following PRISMA guidelines (OSF: 10.17605/OSF.IO/S3R2E). Cross-sectional studies published between 2022 and 2025 reporting the prevalence of depression, anxiety, and/or stress among university students using validated assessment tools were included. Study quality was assessed using the Joanna Briggs Institute checklist. Single-arm meta-analyses of proportions were performed using logit transformation. Subgroup analyses by geographic region, meta-regression, sensitivity analyses, and publication bias assessments were conducted.

**Results:**

Sixty-three studies involving more than 255,000 university students across multiple global regions were included. The pooled prevalence of overall depression was 45.1% (95% CI: 38.5% to 51.8%), with prevalence estimates of 21.9% for mild, 12.8% for moderate, and 4.1% for severe depression. The pooled prevalence of overall anxiety was 43.6% (95% CI: 35.3% to 52.3%), with 23.1% mild, 13.7% moderate, and 6.1% severe anxiety. Overall stress prevalence was 45.6% (95% CI: 32.9% to 58.8%), while high perceived stress affected 14.5% of students. Significant regional variations were observed for depression and anxiety. Meta-regression indicated no consistent temporal trends, although prevalence varied by sample size and, for moderate and severe anxiety, by publication year. Sensitivity analyses confirmed robust findings. Publication bias was detected in mild depression, mild anxiety, and severe anxiety subgroups.

**Conclusions:**

Depression, anxiety, and stress remain highly prevalent among university students in the post-pandemic period, highlighting a sustained mental health burden. Most studies were cross-sectional and relied on self-reported measures, with variability in sample sizes and sociocultural contexts, hindering comparability and generalizability. Despite these constraints, these findings highlight the urgent need for targeted mental health screening, preventive strategies, and accessible psychological support services within higher education settings.

**Supplementary Information:**

The online version contains supplementary material available at https://doi.org/10.1186/s40359-026-05066-4.

## Background

Mental health encompasses an individual’s emotional, psychological, and social well-being. It influences thoughts, emotions, behaviors, interpersonal relationships, and the ability to cope with stress and make decisions. Maintaining good mental health is essential throughout all stages of life and represents a fundamental component of overall health and well-being [1].

University students are considered a particularly vulnerable population for mental health problems due to the unique academic, social, and personal challenges associated with higher education [2, 3]. Academic workload, transitions in social identity, financial pressures, uncertainty regarding future employment, and changes in interpersonal relationships may collectively increase susceptibility to psychological distress during university years. Consequently, recent comparative epidemiological evidence suggests that university students tend to report higher prevalence rates of both anxiety and depression compared with age-matched individuals in the general population, based on aggregated estimates from separate population-based and student-focused studies [4]. However, most available data are derived from indirect comparisons rather than direct head-to-head analyses within the same study populations.

Among the most common mental health conditions affecting university students are depression and anxiety, both of which represent major global public health concerns. Depression is often associated with psychosomatic symptoms, including persistent sadness, low energy, diminished interest in activities, and impaired daily functioning [5]. Anxiety commonly manifests as excessive worry, restlessness, and heightened fear responses to everyday situations (DSM-5) [6]. In addition to anxiety and depression, stress has emerged as an important psychological construct among university students. Stress refers to the psychological and physiological response to internal or external demands perceived as exceeding an individual’s coping capacity. Although stress is conceptually distinct from anxiety and depression, these conditions are closely interrelated and may coexist or contribute to one another, particularly in academically demanding environments [7].

Globally, in 2019, anxiety disorders affected an estimated 301.4 million people (95% UI: 252.6–356.0), while depressive disorders affected 279.6 million people (251.6–310.3) and accounted for the largest proportion of mental disorder disability-adjusted life-years (DALYs) (37.3%) [8]. During the COVID-19 pandemic, the prevalence of anxiety and depression increased by approximately 25%, with young people and women disproportionately affected [9]. University students were particularly vulnerable during this period due to academic disruption, social isolation, remote learning, and uncertainty regarding educational and professional trajectories.

A systematic review and meta-analysis found pooled prevalence rates of 39.0% for anxiety symptoms and 33.6% for depressive symptoms among college students, indicating a substantially greater burden compared with the general population of similar age groups [4]. Furthermore, studies conducted during and after the pandemic demonstrated marked increases in psychological distress among university students across different regions and academic disciplines. A study from Pakistan reported anxiety symptoms in 56% and depression in 32% of university students, with contributing factors including academic responsibilities, economic resources, interpersonal relationships, sexual orientation, and management of free time [10, 11]. Post-pandemic studies among health science students reported depression prevalence approaching 50%, particularly among younger students, those with low social support, and students in earlier academic years [12].

The present review specifically focuses on the post–COVID-19 period, defined as the phase following the relaxation of major pandemic-related restrictions and the progressive return to in-person academic and social activities. Although definitions of the post-pandemic period may vary across studies and regions, recent literature generally characterizes this phase as the transition period after the acute public health emergency, during which universities resumed conventional educational practices while students continued experiencing residual psychological and academic consequences of the pandemic [13]. Emerging evidence suggests that mental health symptoms may persist beyond the acute pandemic phase despite the normalization of many academic and social activities [14].

Recent research has focused on characterizing depression, anxiety, and stress among university students in relation to academic performance during the post-pandemic period. Studies have reported significant negative associations between these mental health conditions and academic outcomes, including lower grade point averages, impaired concentration, and increased test anxiety [15]. Post-pandemic research also suggests that while perceived stress levels among students may have partially normalized, coping behaviours and study commitment remain altered compared with pre-pandemic baselines [14]. Anxiety symptoms have additionally been associated with female gender and poor sleep quality [15]. However, the influence of socioeconomic factors, parental education, and pre-existing mental health conditions on student mental health remains insufficiently understood.

Validated instruments such as the Beck Depression Inventory (BDI) and the Generalized Anxiety Disorder Scale (GAD) are widely used to assess depressive and anxiety symptoms, respectively, and demonstrate strong psychometric properties, including high internal consistency and test–retest reliability [16, 17]. In the present review, emphasis was placed on studies employing validated assessment tools to improve the reliability and comparability of prevalence estimates. Importantly, studies assessing stress were included only when they evaluated general psychological stress or stress-related symptoms rather than formal post-traumatic stress disorder (PTSD) diagnoses.

Despite growing recognition of mental health challenges among university students, evidence characterizing the prevalence of depression, anxiety, and stress during the post-pandemic period remains fragmented. Existing studies vary considerably in methodology, population characteristics, and assessment instruments, limiting the ability to generate comprehensive and comparable prevalence estimates. Furthermore, the influence of sociodemographic and academic factors on these outcomes remains insufficiently explored. Therefore, a systematic synthesis of available evidence is needed to better characterize the current burden of mental health symptoms among university students and inform evidence-based mental health interventions.

Accordingly, this systematic review and meta-analysis aimed to address the following PICOS question: What is the prevalence of anxiety, depression, and stress symptoms among university students during the post–COVID-19 period? The population of interest comprised university students, while the exposure was the period after the acute phase of COVID-19, following the relaxation of major restrictions and the resumption of conventional academic activities. Thus, the aim was to characterize the prevalence of anxiety, depression, and stress symptoms among university students during the broader post pandemic transition period, rather than to describe mental health symptoms exclusively related to COVID-19. To answer this question, we synthesized evidence from observational studies, including descriptive and correlational designs, without experimental manipulation.

## Methods

This study follows Preferred Reporting Items for Systematic Reviews and Meta-Analysis (PRISMA) instructions and the steps mentioned in Cochrane's Handbook of Systematic Reviews of Interventions [18]. The study protocol was also registered in the Open Science Framework (OSF) (https://doi.org/10.17605/OSF.IO/S3R2E).

### Search strategy, data collection, and eligibility criteria

A comprehensive literature search was conducted across PubMed, Web of Science (WoS), MEDLINE, and Scopus databases from inception to August 2025. The search strategy combined Medical Subject Headings (MeSH) terms and free-text keywords related to depression, anxiety, stress, and university students or college students. Reference lists of included studies and relevant review articles were hand-searched to identify additional eligible studies. The search queries that were applied can be seen in Table 1.

**Table 1:** Search queries used in each database

Database	Search query
PubMed	(("university student" OR "college student" OR undergraduate OR postgraduate OR "higher education student") AND (depression OR "depressive symptom" OR anxiety OR "anxious symptom" OR stress OR "psychological distress") AND (prevalence OR epidemiology OR "cross-sectional" OR survey))
Web of Science	TS = ("university student" OR "college student" OR undergraduate OR postgraduate OR "higher education student") AND TS = (depression OR "depressive symptom" OR anxiety OR "anxious symptom" OR stress OR "psychological distress") AND TS = (prevalence OR epidemiology OR "cross-sectional" OR survey)
MEDLINE (Ovid)	("university student".tw. OR "college student".tw. OR undergraduate.tw. OR postgraduate.tw. OR "higher education student".tw.) AND (depression.tw. OR "depressive symptom".tw. OR anxiety.tw. OR "anxious symptom".tw. OR stress.tw. OR "psychological distress".tw.) AND (prevalence.tw. OR epidemiology.tw. OR "cross-sectional".tw. OR survey.tw.)
Scopus	TITLE-ABS-KEY("university student" OR "college student" OR undergraduate OR postgraduate OR "higher education student") AND TITLE-ABS-KEY(depression OR "depressive symptom" OR anxiety OR "anxious symptom" OR stress OR "psychological distress") AND TITLE-ABS-KEY(prevalence OR epidemiology OR "cross-sectional" OR survey)

### Study selection, data extraction and outcomes

Two independent reviewers screened all titles and abstracts retrieved from the search, followed by full-text assessment to determine eligibility. Discrepancies were resolved through discussion or by consultation with a third reviewer. The inclusion criteria were as follows: (a) employed an observational, non-experimental study design (cross-sectional or cohort); (b) reported the prevalence of depression, anxiety, or stress among university or college students during the post-pandemic period (2022–2025); (c) used validated screening instruments for outcome assessment, including but not limited to the Patient Health Questionnaire-9 (PHQ-9) [19]; the Depression Anxiety Stress Scales-21 (DASS-21) [20], the Generalized Anxiety Disorder-7 (GAD-7) [21], the Beck Depression Inventory-II (BDI-II) [22], the Center for Epidemiologic Studies Depression Scale (CES-D) [23], the Self-Rating Anxiety Scale (SAS) [24], and the Perceived Stress Scale (PSS) [25]; (d) provided sufficient numerical data to calculate prevalence estimates (i.e., number of cases and total sample size); and (e) were published in English. Studies were excluded if they: (1) focused on specific clinical populations (e.g., students with pre-existing psychiatric diagnoses); (2) did not report original data; (3) were case reports, editorials, or conference abstracts; or (4) assessed post-traumatic stress disorder (PTSD) as the sole stress outcome using trauma-specific instruments (e.g., Impact of Event Scale [IES], PTSD Checklist [PCL]). Studies that administered IES/PCL alongside a general stress instrument (e.g., DASS-21 Stress subscale, PSS) were retained, although only data from the general stress instrument contributed to the pooled estimates. For each eligible study, data were extracted using a standardized form, capturing study characteristics, participant characteristics, and relevant outcome measures.

The primary outcome was prevalence of the following symptom domains, assessed using validated self-report or diagnostic instruments. For depression: validated scales (PHQ 9, CES D, BDI II, SDS, DASS 21 depression subscale), with the outcome metrics: Overall prevalence (any level of depressive symptoms) and Severity-specific prevalence (mild, moderate, severe). For Anxiety: validated scales (GAD 7, HAM A, HADS-A, SAS, BAI, DASS 21 anxiety subscale), with Overall prevalence and Severity-specific prevalence (mild, moderate, severe). And for stress: validated tools capturing general perceived stress and broader distress-related symptoms, including the DASS-21 stress subscale, PSS-10, PSS-14, and, where there was also administered a general stress measure: IES-6, IES-R, PCL-C, and PCL-5. Outcome metrics comprised overall stress prevalence and high perceived stress prevalence.

It should be noted that the construct of “stress” as assessed in this review was operationalized as general perceived stress and broader distress-related symptoms, conceptually distinct from PTSD. The pooled estimates are therefore based primarily on validated general stress instruments (e.g., PSS, DASS-21 Stress subscale). When some study included additionally administered trauma-related instruments (IES-6, IES-R, PCL-C, PCL-5) as complementary measures, data from these instruments were not aggregated into the pooled prevalence estimates.

In addition, data included study characteristics (author and year of publication, country and region, cross‑sectional design, and recruitment period within the post‑COVID‑19 timeframe) as well as the validated instruments used to assess outcomes (PHQ‑9, CES‑D, BDI‑II, DASS‑21, GAD‑7, HAM‑A, HADS‑A, PSS‑10, PSS‑14, IES‑6, PCL‑5, among others), together with available information on funding and competing interests, which were often unreported. Participant characteristics were also collected, including sociodemographic variables (age, gender, nationality or region of residence, and marital status), academic characteristics (education level, field of study, and institution type), socioeconomic characteristics (household income, monthly allowance, and employment status), and health‑related background (personal or family history of mental disorders and, occasionally, physical health conditions). Outcome data (number of cases and total sample size for each severity level) was extracted. Interrater reliability was assessed using Cohen's kappa; disagreements were resolved by consensus or consultation with a third reviewer.

Severity of depression, anxiety, and stress was defined according to the validated cut-off scores of each instrument as reported by the original study authors. To ensure comparability across instruments, a common four-level severity framework (none/minimal, mild, moderate, severe) was adopted as the reference taxonomy. The following standard thresholds were used when applicable: PHQ-9: 5–9 mild, 10–14 moderate, 15–19 moderately severe, > = 20 severe [19]; DASS-21 Depression subscale: 10–13 mild, 14–20 moderate, 21–27 severe, > = 28 extremely severe [20]; GAD-7: 5–9 mild, 10–14 moderate, > = 15 severe [21]; BDI-II: 14–19 mild, 20–28 moderate, > = 29 severe [22]; PSS-10: 0–13 low, 14–26 moderate, > = 27 high perceived stress [25].

### Risk of bias assessment

The methodological quality of the included cross-sectional studies was independently assessed using the Joanna Briggs Institute (JBI) Critical Appraisal Checklist for Analytical Cross-Sectional Studies [26]. This tool comprises eight domains evaluating the clarity of inclusion criteria, detailed description of study participants and setting, validity and reliability of exposure measurement, use of objective and standard criteria for outcome measurement, identification and management of confounding factors, strategies to address confounders, and appropriateness of statistical analysis.

Each item was rated as “Yes,” “No,” “Unclear,” or “Not applicable.” For each study, a score was calculated based on the number of items rated as “Yes.” Studies were categorized as high quality (≥ 70% of items fulfilled), moderate quality (50–69%), or low quality (< 50%). Two independent reviewers assessed the risk of bias of included studies, and interrater agreement was calculated using Cohen’s kappa statistic. The overall interrater reliability was substantial, with a Cohen’s kappa value of 0.82, indicating high agreement between reviewers. Any discrepancies in risk of bias ratings were resolved through discussion and, when necessary, consultation with a third reviewer to reach consensus. The quality assessment results were summarized in tabular form and considered in the interpretation of the findings.

### Data analysis

All statistical analyses were performed using R software version 4.3.2 [27]. Meta-analyses were conducted using the meta package version 8.2–1 [28] and the metafor package version 4.8–0 [29].

### Effect size calculation

Single-arm meta-analysis of proportions was performed using the logit transformation method. The logit transformation (log odds) was applied to stabilize the variance of proportions and ensure that confidence intervals remain within the valid range of 0–1. Effect sizes were calculated using the escalc function with measure = PLO (proportion, logit transformation). A continuity correction of 0.5 was applied to studies with zero events to avoid computational issues. Pooled prevalence estimates were back-transformed from the logit scale to proportions using the inverse logit function for interpretation.

Any outcome reported by less than ten studies was excluded from further analysis due to insufficient data to permit a meaningful synthesis. The small number of contributing studies precluded the performance of a reliable meta-analysis and did not allow for a robust narrative synthesis. Moreover, the limited evidence base increases the risk of imprecision and reduces the reliability and generalizability of the findings. The small number of studies also heightens susceptibility to selective outcome reporting and potential publication bias, further limiting confidence in any derived conclusions.

### Assessment of heterogeneity

Heterogeneity across studies was assessed using multiple statistical approaches. The Cochran Q statistic, a chi-squared test, was used to evaluate whether observed differences in study results are due to chance alone, with a *p*-value less than 0.10 indicating significant heterogeneity. The I-squared (I2) statistic was calculated to quantify the proportion of total variability due to true between-study heterogeneity rather than sampling error [30]. I-squared values were interpreted as follows: 0–25% indicates low heterogeneity, 25–50% indicates moderate heterogeneity, 50–75% indicates substantial heterogeneity, and values exceeding 75% indicate considerable heterogeneity. Additionally, tau-squared (τ2) was estimated to represent the between-study variance in the random-effects model, providing the actual magnitude of heterogeneity on the effect size scale.

### Meta-analytic model selection

Given the expected clinical, methodological, and statistical heterogeneity inherent in pooling prevalence data across diverse populations, geographic regions, cultural contexts, and measurement instruments, a random-effects model using the restricted maximum likelihood (REML) estimation method was applied for all analyses [31]. This approach accounts for both within-study sampling error and between-study variability in true prevalence rates, yielding pooled estimates and confidence intervals that appropriately reflect the uncertainty across heterogeneous study populations. Confidence intervals for individual study proportions were calculated using the Clopper-Pearson exact method.

### Subgroup analysis

Subgroup analyses were performed to explore potential sources of heterogeneity. Studies were stratified by: (1) geographic region (Asia, Europe, North America, South America, Middle East, and Africa); and (2) measurement instrument type (e.g., PHQ-9, DASS-21, GAD-7, BDI, CES-D, SAS, PSS for depression and anxiety outcomes; DASS-21 Stress subscale, PSS for stress outcomes). For each subgroup, a separate random-effects meta-analysis was conducted using REML estimation and pooled prevalence was calculated with 95% confidence intervals. The test for subgroup differences (Q-test for moderators) was performed to assess whether prevalence significantly varied across regions, with a *p*-value less than 0.05 for the interaction test indicating statistically significant subgroup differences.

### Meta-regression analysis

Mixed-effects meta-regression was conducted using REML to examine the association between study-level covariates and prevalence estimates. The following covariates were examined: (1) publication year (continuous); (2) sample size (log10-transformed continuous); (3) mean participant age (continuous, where reported); and (4) proportion of male participants (%, continuous, where reported). For each model, the beta coefficient represents the change in log-odds of prevalence per unit increase in the covariate. Statistical significance was assessed at alpha = 0.05, and R-squared was calculated to determine the proportion of between-study heterogeneity explained.

### Sensitivity analysis

Leave-one-out sensitivity analysis was performed to assess the robustness of individual studies on pooled estimates. Each study was sequentially excluded and the pooled prevalence recalculated. Results were considered stable if the range of leave-one-out estimates was less than 5 percentage points. Additionally, instrument-based sensitivity analysis was conducted by sequentially excluding all studies using each specific measurement instrument and recalculating pooled prevalence to assess whether results were dependent on any single instrument type.

### Publication bias assessment

Publication bias was assessed using funnel plot visual inspection and Egger linear regression test [32]. Additionally, Peters test [33], which regresses the effect size against the inverse of sample size, was conducted given its superior performance for proportion meta-analyses. It is important to note that both Egger and Peters tests have recognized limitations in meta-analyses of proportions characterized by substantial heterogeneity, where these tests detect the relationship between effect size and precision but cannot reliably distinguish publication bias from other causes of funnel plot asymmetry such as genuine small-study effects, clinical heterogeneity related to study size, or model misspecification [34, 35]. Therefore, significant results should be interpreted as indicative of potential small-study effects rather than definitive evidence of publication bias.

### Statistical significance

All statistical tests were two-sided with a significance level of α = 0.05 unless otherwise specified.

## Results

### Search strategy and study selection

**Fig. 1:**
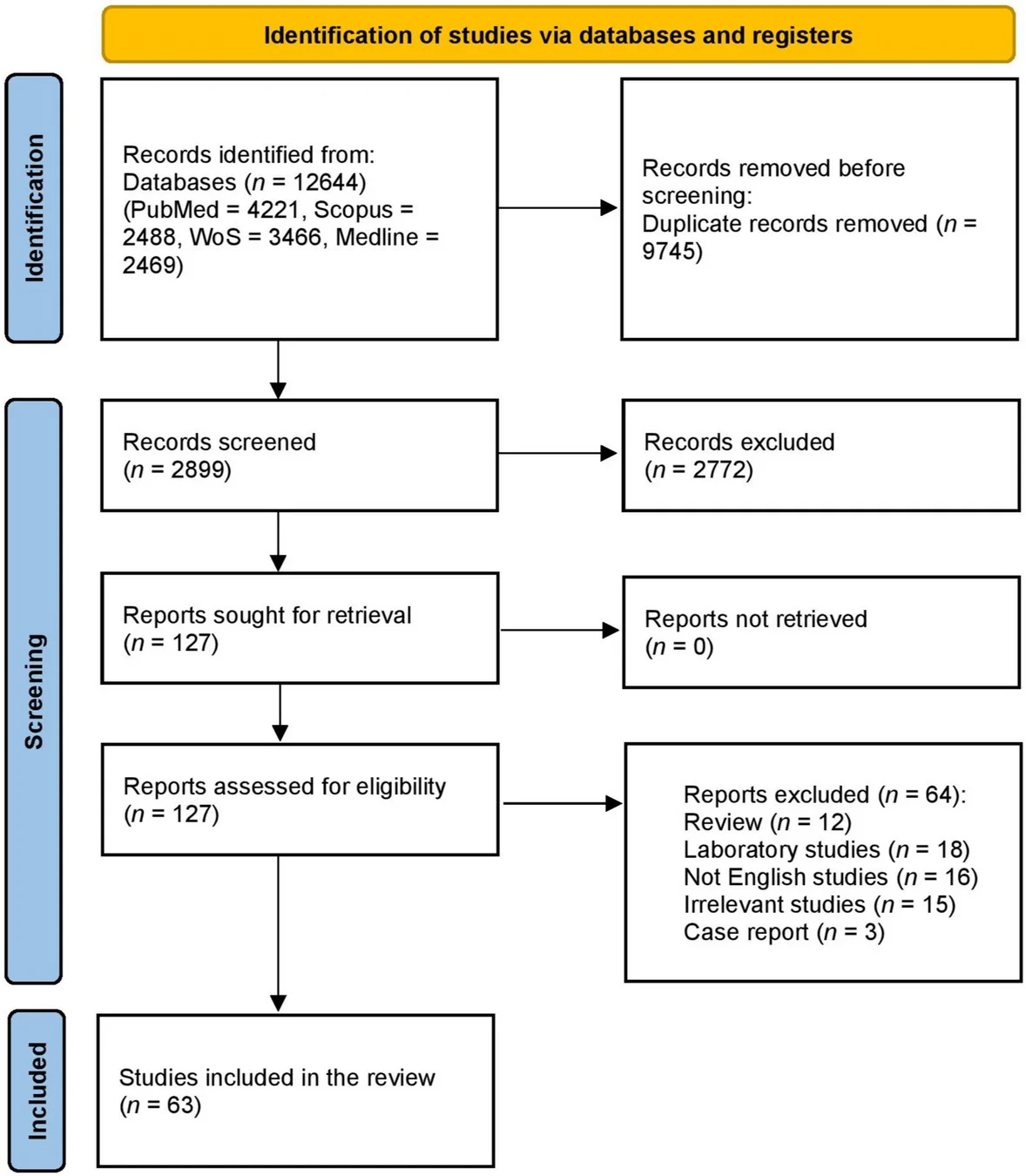
PRISMA diagram flow for the systematic review

The initial database search identified a total of 12,644 records. After removing duplicates, 2899 records remained for screening. Following the review of titles and abstracts, 2772 records were excluded for not meeting the inclusion criteria. The full texts of 127 articles were assessed for eligibility, of which 63 studies met the predefined criteria and were included in the final systematic review. The exclusion reasons were identified and reported as follows: review articles (12 studies), laboratory or non-clinical studies (18 studies), non-English language publications (16 studies), studies with irrelevant population or outcomes (15 studies), and case reports (3 studies). The study selection process is presented in the PRISMA flow diagram (Fig. 1).

### Summary of included studies

A total of 63 cross-sectional studies published between 2022 and 2025 were included in this systematic review, encompassing more than 255,000 university students across multiple geographical regions, including Asia, the Middle East, Europe, North America, Latin America, and Africa [36–98]. The largest proportion of studies was conducted in China, followed by studies from the United States (U.S), India, Saudi Arabia, Kuwait, Spain, and other countries, reflecting a broad international representation of post-pandemic university populations.

All included studies employed a cross-sectional design and assessed mental health outcomes during the post-COVID-19 period, with recruitment periods ranging from early 2022 to late 2024. Sample sizes varied considerably, from small cohorts of fewer than 100 participants to large national surveys exceeding 40,000 students, with a median sample size across studies was 1291 (IQR: 498–4592; range: 76–41,620), highlighting substantial heterogeneity in study scale.

Mental health outcomes were predominantly assessed using self-report questionnaires, with most studies relying on online or paper-based surveys. A small number of studies incorporated face-to-face supervised self-reporting or interview-based approaches, although self-administered methods remained the primary mode of data collection.

Depression was most evaluated using the Patient Health Questionnaire (PHQ) family of instruments, particularly PHQ-9, followed by Center for Epidemiological Studies Depression Scale (CES-D), Depression, Anxiety and Stress Scale-21 (DASS-21), depression subscle, BDI-II, and Self-Rating Depression Scale (SDS). Anxiety symptoms were frequently assessed using the GAD-7, alongside other validated tools such as DASS-21, anxiety subscale, Self-Rating Anxiety Scale (SAS), Hamilton Anxiety Rating Scale (HAM-A), Hospital Anxiety and Depression Scale-Anxiety subscale (HADS-A), and Beck Anxiety Inventory (BAI).

Stress-related outcomes were less consistently reported and were predominantly measured using the Depression, Anxiety and Stress Scale-21 (DASS-21) and the Perceived Stress Scale (PSS-10 and PSS-14). A minority of studies additionally administered trauma-related instruments, namely the Impact of Event Scale (IES-6 and IES-R) and the PTSD Checklist (PCL-C and PCL-5), as complementary measures; however, only data from general stress instruments contributed to the pooled prevalence estimates. Several studies did not report stress outcomes or excluded stress components from the final analysis. More details were presented in Table 2.

**Table 2:** Summary of the included studies

Author	Year	Study design	Country	University/Campus	Recruitment duration	Sample size	Method of reporting (self-report/interview)	Screening/Diagnostic tool of Depression	Screening/Diagnostic tool of Anexity	Screening/Diagnostic tool of Stress
Ahmed et al	2025	Cross-sectional study	Sudan	Public or private medical schools	November 2024	1891	Self-report	PHQ-9	GAD-7	PSS-10
Alawadhi et al	2025	Cross-sectional study	Kuwait	Kuwait University	February to April 2023	744	Self-report	DASS-21	DASS-21	The stress component of the DASS-21 was excluded from the analysis
Arias-Coronel et al	2025	Cross-sectional study	Colombia	Universidad Santiago de Cali	2023	394	Self-report	PHQ-9	Hamilton Anxiety Scale	NR
Barr-Porter et al	2025	Cross-sectional study	USA	University of Kentucky and University of Maine	January 2023	536	Self-report	Single self-reported item on professional diagnosis	Single self-reported item on professional diagnosis	NR
de Alencar Ribeiro et al	2025	Cross-sectional study	Brazil	Federal University of Piauí	February to June 2023	179	Self-report	DASS-21	DASS-21	DASS-21
Dubovi & Edwards	2025	Cross-sectional study	USA	Cornell University	May 1–30, 2023	1019	Self-report	PHQ-4	PHQ-4	PHQ-4
Erbil et al	2025	Cross-sectional study	Türkiye	Ordu University	March 1 – May 28, 2022	500	Self-report (face-to-face supervised)	DASS-21	DASS-21	DASS-21
Flesaker et al	2025	Cross-sectional study	USA	135 institutions (Healthy Minds study)	2022–2023	19,265	Self-report (Online survey)	PHQ-9	NR	NR
Frausing et al	2025	Cross-sectional study	Denmark	VIA University College	May 2023	152	Self-report (Electronic survey)	WHO-5 Well-Being Index	HADS-A	PSS-10
Han et al. (A)	2025	Cross-sectional study	China	106 universities in 31 provinces of China	June 20 – August 31, 2022	8932	Self-report questionnaire, face-to-face administration	PHQ-9	GAD-7	NR
Han et al. (B)	2025	Cross-sectional study	China	106 universities in 31 provinces of China	October 9—22, 2023	41,620	Self-report	CES-D	GAD-7	NR
Kong et al	2025	Cross-sectional study	China	Chongqing Medical University	September 2023	6378	Self-report	PHQ-9	GAD-7	NR
Kunda et al	2025	Cross-sectional study	Rwanda	University of Global Health Equity (UGHE)	6 May—31 May 2024	595	Self-report	PHQ-9	GAD-7	NR
Pratummanee et al	2025	Cross-sectional study	Thailand	Southern Thailand universities	September to December 2022	1611	Self-report	PHQ-9, Thai version	NR	NR
Sudershan et al	2025	Cross-sectional study	India	Institutions across the Kashmir division	January 2024 to April 2024	1471	Self-report	PHQ-9	GAD-7	PSS
Webb et al	2025	Cross-sectional study	UAE	Higher Colleges of Technology (across 16 campuses)	Nov 2022—Feb 2023	7244	Self-report	Single-item question on diagnosed disorders	GAD-7	Single-item question on stress levels
Xu et al	2025	Cross-sectional study	China	Hubei University	May to August 2024	2804	Self-report	NR	GAD-7	NR
Alotaibi et al	2024	Cross-sectional study	Kuwait	Kuwait University	January 2024	1142	Self-report	DASS-21	DASS-21	DASS-21
Chen et al. (A)	2024	Cross-sectional study	China	Wenzhou Medical University (Author origin)	T1: Dec 14–20, 2022 T2: Mar 1–7, 2023 T3: Jun 1–7, 2023 T4: Nov 25–30, 2023	2046T1: *N* = 689 T2: *N* = 456 T3: *N* = 300 T4: *N* = 601	Self-report	PHQ-9	GAD-7	PCL-C
Chen et al. (B)	2024	Cross-sectional study	China	North Sichuan Medical College	October 2023	4858	Self-report	DASS-21 (Chinese Version)	DASS-21 (Chinese Version)	DASS-21 (Chinese Version)
Feng et al	2024	Cross-sectional study	China	Quzhou University	August 2022—September 2022	2764	Self-report (Online questionnaires)	BDI-II	NR	NR
Gao et al	2024	Cross-sectional study	China	Five vocational universities in Wen Jiang District, Chengdu	January to February 2022	3049	Interview (face-to-face, self-report questionnaires)	PHQ-9	GAD-7	GHQ-12
Govindan et al	2024	Cross-sectional study	India	Sri Venkateshwaraa Medical College Hospital & Research Centre	January 2022—June 2022	347	Self-administered questionnaire	NR	HAM-A	NR
Jiang et al	2024	Cross-sectional study	China	25 university departments	May to August 2022	757	Self-report	SDS	SAS	NR
Kharroubi et al	2024	Cross-sectional study	Lebanon	American Univ. of Beirut, Univ. of Balamand, and Lebanese University	Fall 2023 semester (October-December)	361	Self-report	PHQ-9	GAD-7	PSS
Kilic et al	2024	Cross-sectional study	Turkey	Yozgat Bozok University	2022	518	Self-report	DASS-21	DASS-21	DASS-21
Lai et al	2024	Cross-sectional study	USA	University of Massachusetts Lowell	September to December 2022	222	Self-report	PHQ-9	GAD-7	PSS
Lee et al	2024	Cross-sectional study	China	Four colleges/universities located in three provinces (Shanxi, Hunan, Guangdong	March 15, 2022, to April 3, 2022	1359	Self-report	PHQ-9	GAD-7	IES-6
Li et al. (A)	2024	Cross-sectional study	China	104 universities in 31 provinces of China	September 2022	30,475	Self-report	CES-D	GAD-7	NR
Li et al. (B)	2024	Cross-sectional study	China	Three universities in Daqing City	March to April 2024	7845	Self-report	GHQ-20	GHQ-20	PSTR
Li et al. (C)	2024	Cross-sectional study	China	Three universities in Changzhou City	March to May 2023	7484	Self-report	PHQ-9	GAD-7	PCL-5 (PTSD Checklist for DSM-5)
Liu et al	2024	Cross-sectional study	China	Zunyi Medical University	September to November 2023	2066	Self-report	NR	GAD-7	NR
Makki et al	2024	Cross-sectional study	Saudi Arabia	Taibah University	July 2022	520	Self-report	DASS-21	DASS-21	DASS-21
Martínez-Esquivel et al	2024	Cross-sectional study	Costa Rica	University of Costa Rica	2022	76	Self-report	BDI-II	BAI	NR
Milić et al	2024	Cross-sectional study	Croatia	10 institutions (e.g., Catholic Univ. of Croatia, Univ. of Zadar, Univ. of Split)	March 2023	2137	Self-report	PHQ-9	GAD-7	NR
Murthy et al	2024	Cross-sectional study	India	Chettinad Academy of Research and Education (CARE)	July to September 2024	223	Self-report	PHQ-10	NR	NR
Saqib & Gazerani	2024	Cross-sectional study	Norway	Univ. of Oslo, OsloMet, NTNU, Univ. of Bergen, UiT, USN, etc	Nov 2023	132	Self-report	PHQ-9	GAD-7	NR
Simionescu et al	2024	Cross-sectional study	Romania	University of Bucharest	Jan 2022	422	Self-report	NR	Custom 5-item scale	Miller and Smith Scale (20-item)
Su et al	2024	Cross-sectional study	China	Xi’an Medical College and Shaanxi University of Traditional Chinese Medicine	December 5, 2022, to January 15, 2023	1327	Self-report	PHQ-2	GAD-7	NR
Sun et al	2024	Cross-sectional study	China	Three colleges and universities in Fujian Province	February 2023	498	Self-report	PHQ-9	GAD-7	NR
Wang et al	2024	Cross-sectional study	China	Suzhou Vocational Health College, Anhui Medical College, and Anhui Medical University	Oct to Dec 2022	7617	Self-report	PHQ-9	GAD-7	NR
Wu et al	2024	Cross-sectional study	China	Zhejiang Technical Institute of Economics	10—30 Dec 2022	1255	Self-report	DASS-21	DASS-21	DASS-21
Xin et al	2024	Cross-sectional study	China	Peking Union Medical College	January 5 to February 9, 2023	4254	Self-report (Online questionnaire)	PHQ-9	GAD-7	IES-6 (for PTSD symptoms)
Xu et al	2024	Cross-sectional study	China	Four colleges/universities in Shanxi, Hunan, and Guangdong	October 17 to 29, 2023	18,578	Self-report (Online questionnaire)	PHQ-2	GAD-2	NR
Yaghmour et al	2024	Cross-sectional study	Saudi Arabia	King Abdulaziz University	January 2023 to February 2023	382	Self-report	DASS-21	DASS-21	DASS-21
Alageel et al	2023	Cross-sectional study	Saudi Arabia	King Saud Bin Abdulaziz University for Health Sciences (KSAU-HS)	May 2023	434	Self-report	PHQ-9	GAD-7	NR
Cao et al	2023	Cross-sectional study	China	Tongji University	September 23 to October 23, 2022	1770	Self-report	NR	GAD-7	PSS-14
Cao & Zheng	2023	Cross-sectional study	China	Shaanxi College of Communications Technology	April to May 2022	4705	Self-report	College Students' Life Stress and Mental Health Questionnaire—Depression subscale (9 items)	NR	College Students' Life Stress and Mental Health Questionnaire—Life Stressors subscale (16 items) & Stress Response subscale (16 items)
Chen et al	2023	Cross-sectional study	China	Various universities in Hebei Province	December 31, 2022, to January 7, 2023	22,624	Self-report	PHQ-9	GAD-7	IES-R
Deng & Zhang	2023	Cross-sectional study	China	Zhanjiang Preschool Education College and Guangdong Medical University	August 1–7, 2022	1083	Self-report	HDRS-17	SAS	NR
He et al	2023	Cross-sectional study	China	Army Medical University	April 9, 2022—May 10, 2022	387	Self-report	PHQ-9	GAD-7	NR
Huang & Liu	2023	Cross-sectional study	China	Fujian Medical University and Lanzhou University	November 6, 2022, to December 2, 2022	1176	Self-report	PHQ-9	GAD-7	NR
Kavvadas et al	2023	Cross-sectional study	Greece	Aristotle University of Thessaloniki	10 November 2022 to 25 November 2022	1497	Self-report	DASS-21	DASS-21	DASS-21
Liu et al	2023	Cross-sectional study	China	Jiangxi Normal University and Jiangxi Vocational College of Finance and Economics	February 22—September 12, 2022	2381	Self-report	PHQ-9	GAD-7	PSS
Lv et al	2023	Cross-sectional study	China	Chongqing Medical University	18 January—18 February 2022	915	Self-report	CES-D	SAS	NR
Martínez-Líbano et al	2023	Cross-sectional study	Chile	Universidad Andrés Bello and Universidad de las Américas	November 2022	1062	Self-report	DASS-21	DASS-21	DASS-21
Nahrin et al	2023	Cross-sectional study	Bangladesh	Jahangirnagar University, Dhaka	4–11 September 2022	1523	Self-report	PHQ-9	GAD-7	NR
Pérez et al	2023	Cross-sectional study	Spain	Complutense University of Madrid (UCM)	February to March 2022	6798	Self-report	PHQ-9	GAD-7	NR
Qiao et al	2023	Cross-sectional study	China	Various universities	April 5th to May 29th, 2022	384	Self-report	PHQ-9	GAD-7	NR
Rajasekhar et al	2023	Cross-sectional study	India	68 universities in Jodhpur City (e.g., AIIMS Jodhpur)	September 2018 to October 2019	2035	Self-report	DASS-21	DASS-21	DASS-21
Ramón-Arbués et al	2023	Cross-sectional study	Spain	San Jorge University	February to May 2022	339	Self-report	HADS	HADS	NR
Xia et al	2023	Cross-sectional study	China	Bengbu Medical College, Wannan Medical College, and University of Science and Technology of China	March 7 to March 27, 2022	1016	Self-report (Online questionnaire)	PHQ-9	GAD-7	NR
Li et al	2022	Cross-sectional study	China	Lu’an Hospital (Anhui Medical Univ.) and Anhui Univ. of Traditional Chinese Medicine	April 6–20, 2022	6905	Self-report (online questionnaire)	SDS	SAS	NR

Abbreviations: *NR* Not reported, *PHQ-9* Patient Health Questionnaire-9, *PHQ-10* Patient Health Questionnaire-10, *PHQ-4* Patient Health Questionnaire-4, *PHQ-2* Patient Health Questionnaire-2, *CES-D* Center for Epidemiological Studies Depression Scale, *BDI-II* Beck Depression Inventory-II, *HDRS-17* 17-item Hamilton Depression Rating Scale, *SDS* Self-Rating Depression Scale, *WHO-5* World Health Organization-5 Well-Being Index, *GHQ-20* 20-item General Health Questionnaire, *DASS-21* Depression, Anxiety and Stress Scale-21, *GAD-7* Generalized Anxiety Disorder-7, *GAD-2* Generalized Anxiety Disorder-2, *HAM-A* Hamilton Anxiety Rating Scale, *HADS-A* Hospital Anxiety and Depression Scale – Anxiety subscale, *BAI* Beck Anxiety Inventory, *SAS* Self-Rating Anxiety Scale, *PSS* Perceived Stress Scale, *PSS-10* 10-item Perceived Stress Scale, *PSS-14* 14-item Perceived Stress Scale, *GHQ-12* 12-item General Health Questionnaire, *IES-6* Impact of Event Scale-6, *IES-R* Impact of Event Scale-Revised, *PCL-C* PTSD Checklist – Civilian Version, *PCL-5* PTSD Checklist for DSM-5, *PSTR* Psychological Stress Tolerance Index, *HADS* Hospital Anxiety and Depression Scale, *DASS* Depression, Anxiety and Stress Scale, *USA* United States of America, *UAE* United Arab Emirates

### Baseline characteristics of the participants

The baseline characteristics of participants across the included studies are summarized in Table 3. Overall, the studies involved diverse university student populations from multiple countries and regions, with substantial variability in demographic, socioeconomic, and academic characteristics. Across the included studies, participants were predominantly young adults, with mean ages generally ranging from approximately 18 to 23 years. Where reported, mean ages clustered around the early twenties, reflecting typical university-aged populations.

**Table 3:** Baseline characteristics of the included participants

Author	Year	Age, Mean (SD)	Male gender, N (%)	Region of Residence (Nationality), N (%)	Education Level, N (%)	Economic Status, N (%)	Employment Status, N (%)	Family history of mental disorders, N (%)	History of mental disorders, N (%)	Marital status, N (%)	Type of Faculty, N (%)
Ahmed et al	2025	22.5 (2.4)	696 (36.8%)	Sudan: 1164 (61.6%) Arabic Gulf: 373 (19.7%) North Africa: 301 (15.9%) Central Africa: 33 (1.7%) Other: 20 (1.1%)	1st: 176 (9.3%) 2nd: 308 (16.3%) 3rd: 398 (21.0%) 4th: 372 (19.7%) 5th: 462 (24.4%) 6th: 175 (9.3%)	Middle: 1198 (63.4%) Lower-middle: 282 (14.9%) Upper-middle: 244 (12.9%) Low: 125 (6.6%) High: 42 (2.2%)	Unemployed: 1512 (80.0%) Employed: 217 (11.5%) Freelancer: 162 (8.6%)	NR	NR	NR	Medical Students: 1891 (100%)
Alawadhi et al	2025	18–19: 281 (37.8%) 20–21: 252 (33.9%) ≥ 22: 211 (28.4%)	110 (14.8%)	Kuwaiti: 648 (87.1%) Non-Kuwaiti: 96 (12.9%)	NR	Average: 543 (73.0%) Above average: 163 (21.9%) Below average: 38 (5.1%)	NR	79 (10.6%)	181 (24.3%)	Single: 682 (91.7%) Married: 62 (8.3%)	Arts: 291 (39.1%) Sciences: 278 (37.4%) Health Sciences Center (HSC): 175 (23.5%)
Arias-Coronel et al	2025	NR	133 (33.8%)	Urban: 326 (82.7%) Rural: 68 (17.3%)	NR	1 (lowest): 37 (9.4%) 2: 102 (25.9%) 3: 140 (35.5%) 4: 74 (18.8%) 5: 36 (9.1%) 6 (highest): 5 (1.3%)	NR	NR	125 (31.7%)	Single: 351 (89.1%) Other: 43 (10.9%)	Health: 235 (59.6%) Business: 43 (10.9%) Law: 29 (7.4%) Basic Sciences: 27 (6.9%) Engineering: 22 (5.6%) Education: 20 (5.1%) Humanities: 16 (4.1%)
Barr-Porter et al	2025	19.93 (1.42)	149 (27.8%)	White: 441 (82.3%) Hispanic or Latino: 12 (2.2%) Black or African American: 7 (1.3%) Native American or American: 24 (4.5%) Indian or Asian/Pacific Islander: 14 (2.6%) Other: 9 (1.7%)	Freshman: 133 (24.8%) Sophomore: 152 (28.4%) Junior: 115 (21.5%) Senior: 109 (20.3%)	High: 205 (38.24%) Marginal: 117 (21.8%) Low: 75 (13.99%) Very low: 112 (20.9%)	NR	NR	NR	NR	NR
de Alencar Ribeiro et al	2025	17–19: 19 (10.6%) 20–24: 137 (76.5%) 25–30: 17 (9.5%) 31–39: 3 (1.7%) 40–49: 3 (1.7%)	56 (31.3%)	Brazil: 179 (100%)	NR	No income: 5 (2.8%) < 1 minimum wage: 39 (21.8%) 1–3 minimum wage: 79 (44.1%) 3–5 minimum wage: 33 (18.4%) 5–7 minimum wage: 16 (8.9%) 7–10 minimum wage: 5 (2.8%) > 10 minimum wage: 2 (1.1%)	Employed: 21 (11.7%) Not Employed: 158 (88.3%)	NR	NR	Single: 169 (94.4%) Married: 6 (3.4%) Common-law Marriage: 3 (1.7%) Divorced: 1 (0.6%)	Nursing Department: 179 (100%)
Dubovi & Edwards	2025	NR	Cisgender Men: 315 (36.2%) Cisgender Women: 501 (57.6%) TGNB: 54 (6.2%)	NR	Undergraduate: 602 (59.1%) Graduate: 450 (44.2%) Professional: 38 (3.9%)	NR	NR	NR	NR	NR	NR
Erbil et al	2025	NR	0 (0%)	All participants from the Black Sea Region of Turkey (Turkish nationality)	1st: 153 (30.6%) 2nd: 121 (24.2%) 3rd: 145 (29.0%) 4th: 81 (16.2%)	NR	NR	NR	19 (3.8%)	Married: 5 (1.0%) Single: 495 (99.0%)	Health Sciences: 264 (52.8%) Education: 236 (47.2%)
Flesaker et al	2025	20.19 (1.73)	7769 (40.3%)	African American/Black: 5598 (29.1%) American Indian/Alaskan Native: 105 (0.5%) Asian: 3847 (20.0%) Hispanic/Latin(x): 4320 (22.4%) Middle Eastern/Arab/Arab American: 451 (2.3%) Multiracial: 4882 (25.3%) Native Hawaiian/Pacific Islander: 62 (0.3%)	NR	NR	NR	NR	NR	NR	Public: 11,074 (57.5%) Private: 8191 (42.5%) Community College: 3067 (15.9%)
Frausing et al	2025	28.7 (21–52)#	21 (13.8%)	Danish: 152 (100%)	1st: 55 (36.2%) 2nd: 46 (30.3%) Final: 51 (33.5%)	NR	NR	NR	NR	NR	Educational Program for Psychomotor Therapy VIA University College University College Copenhagen
Han et al. (A)	2025	20.51 (1.667)	2847 (38.9%)	Western China: 2,346 (32.1%) Middle China: 1,974 (27.0%) Eastern China: 2,993 (40.9%) Han: 6475 (88.5%) Ethnic minorities: 838 (11.5%)	Junior college students: 743 (10.2%) Undergraduate students: 6261 (85.6%) Postgraduate students: 309 (4.2%)	≤ 3000 RMB: 2491 (34.1%) ≥ 3001 RMB: 4822 (65.9%)	NR	NR	NR	NR	Social sciences: 3069 (42.0%) Non–social sciences: 4244 (58.0%)
Han et al. (B)	2025	NR	17,212 (41.4%)	Area Eastern: 14,657 (35.2%) Westward: 10,300 (24.7%) Central: 16,663 (40.0%/) Ethnicity Han: 38,360 (92.2%) Minority: 3260 (7.8%)	Freshman: 23,878 (57.4%) Sophomore: 10,605 (25.5%)Junior: 5479 (13.2%)Senior: 1658 (4%)	NR	NR	NR	NR	NR	NR
Kong et al	2025	20.98 (3.37)	2335 (36.6%)	NR	First-year college students: 4266 (66.89%) First-year master’s students: 2112 (33.11%)	NR	NR	NR	NR	NR	NR
Kunda et al	2025	63 (12.5)	121 (20.3%)	Burera: 282 (47.4%) Kirche: 209 (35.1%) Kayonza: 104 (17.5%)	NR	Health Insurance: Yes: 595 (100%) (98.7% Community-based)	Employed: 534 (89.7%) Unemployed: 61 (10.3%)	85 (14.3%)	NR	Married/Cohabiting: 356 (59.8%) Widowed: 202 (33.9%) Single/Divorced/Separated: 37 (6.2%)	NR
Pratummanee et al	2025	19.4 (1.12)	317 (19.7%)	Rural: 1053 (65.4%) Urban: 558 (34.6%)	All participants were first-year university students	Monthly Income: • < 5,000 Baht: 1118 (69.4%) • 5,001–10,000 Baht: 451 (28%) • 10,001–15,000 Baht: 30 (1.9%) • > 15,000 Baht: 12 (0.7%)	NR	NR	87 (5.4%)	Stable Partner: Yes: 764 (47.4%) No: 847 (52.6%)	Humanities & Social Sciences: 1095 (68%) Science & Technology: 332 (20.6%) Health Sciences: 184 (11.4%)
Sudershan et al	2025	21.66 (1.94)	575 (39.1%)	Urban: 572 (38.88%) Rural: 899 (61.11%)Nationality/Religion: Muslim: 1404 (95.44%)Hindu: 57 (3.87%)Sikh: 8 (0.54%)Buddhist: 2 (0.13%)	NR	Monthly Income: < ₹10,000: 558 (37.93%) ₹10,000–20,000: 260 (17.67%) ₹21,000–30,000: 158 (10.74%) ₹31,000–40,000: 109 (7.40%) ₹41,000–50,000: 106 (7.20%) > ₹51,000: 280 (19.03%)	NR	NR	NR	Married: 115 (7.81%) Unmarried: 1356 (92.18%)	NR
Webb et al	2025	18–24: 6954 (96%)	2,685 (37.1%)	National Study: All participants were Emirati nationals	NR	Household Income: < AED 10,000: 1,512 (20.9%) AED 10,000–30,000: 3,804 (52.5%) AED 30,000–60,000: 1,332 (18.4%) > AED 60,000: 596 (8.2%)	Employed: 418 (5.8%) Unemployed: 6,826 (94.2%)	NR	NR	Single: 6,812 (94.0%) Married: 384 (5.3%) Divorced/Separated/Widowed: 48 (0.7%)	NR
Xu et al	2025	20.15 (1.21)	966 (34.84%)	Hubei Province, China (100%)	Third-year: 1280 (45.65%) Fourth-year: 1524 (54.35%)	NR	NR	NR	NR	NR	Arts: 2060 (73.47%)Science: 744 (26.53%)
Alotaibi et al	2024	19.9 (2.1)	303 (26.5%)	Citizen: 900 (78.8%) Resident: 242 (21.2%)	1st: 337 (29.512nd: 323 (28.28%) 3rd: 216 (18.91%) 4th: 266 (23.29%)	Low: 148 (13.0%) Medium: 891 (78.0%) High: 103 (9.0%)	NR	NR	NR	Single: 981 (85.9%) Married: 134 (11.7%) Divorced: 25 (2.2%) Widowed: 2 (0.2%)	Health & Scientific: 623 (54.6%) Science: 140 (12.3%) Allied Health: 353 (30.9%) Engineering: 130 (11.4%) Art & Humanity: 519 (45.4%) Education: 259 (22.7%) Sharia/Islamic: 116 (10.2%) Business Admin: 144 (12.6%)
Chen et al. (A)	2024	NR	442 (21.6%)	China: 2046 (100%) (Hebei Province)	Freshman: 957 (46.85%)Sophomore: 811 (39.6%)Junior:228 (11.14%)Senior: 50 (2.44%)	NR	NR	NR	NR	NR	Medical University: 2046 (100%)
Chen et al. (B)	2024	19.32 (0.66)	1893 (38.96)	China: 4858 (100%) (Hebei Province)	2nd: 4858 (100%)	NR	NR	NR	NR	NR	NR
Feng et al	2024	19.9 (1.2)	854 (30.9%)	China: 2764 (100%)	Freshman: 835 (30.2%) Sophomore: 1197 (43.3%) Junior: 536 (19.4%) Senior: 196 (7.1%)	NR	NR	NR	NR	NR	NR
Gao et al	2024	20.9 (0.02)	903 (29.6%)	Han: 2725 (89.4%) Minority: 324 (10.6%)	NR	Very good: 107 (3.5%) Average: 2,306 (75.6%) Poor: 636 (20.9%)	NR	NR	NR	No love: 2192(71.9%)In love: 832 (27.3%) Married: 25 (0.8%)	Students from several higher vocational colleges in Wenjiang District
Govindan et al	2024	23 (2)	91 (26.2%)	Puducherry, India (All participants from this region)	Undergraduate: 327 (94.2%) Postgraduate: 20 (5.8%)	NR	NR	NR	13 (3.7%)	NR	Medical and Para-medical courses (All participants)
Jiang et al	2024	20.90 (1.48)	274 (36.2%)	Han Chinese: 350 (46.23%) Uyghur: 312 (41.19%) 11 other ethnic groups: 95 (12.55%)	1st: 190 (25.1%)2nd: 189 (25.0%)3rd: 189 (25.0%)4th: 189 (25.0%)	NR	NR	NR	NR	NR	Medical Students (from 25 university departments across 68 academic disciplines)
Kharroubi et al	2024	20.29 (2.025)	126 (48.3%)	Lebanese: 241 (92.3%) Non-Lebanese: 20 (7.7%)	NR	< $100: 82 (31.4%) $100–300: 93 (35.6%) $300–500: 49 (18.8%) ≥ $500: 37 (14.2%)	No job: 189 (72.4%) Employed: 72 (27.6%)	NR	Previous psychological/psychiatric treatment: 60 (23%)	NR	Health-related majors: 69 (26.4%) Non-health-related majors: 192 (73.6%)
Kilic et al	2024	18–19: 159 (30.7%) 20: 151 (29.2%) 21: 118 (22.8%) ≥ 22: 90 (17.4%)	168 (32.4%)	CDI Dormitory: 305 (58.9%) Private Dormitory: 52 (10.0%) Student House: 41 (7.9%) With Family/Relative: 120 (23.2%)	Associate Degree: 277 (53.5%) Undergraduate: 241 (46.5%)	NR	NR	NR	NR	NR	NR
Lai et al	2024	20.3 (2.44)	41 (18.5%)	Non-Hispanic White: 97 (43.7%) African American: 30 (13.5%) Latino/Hispanic: 43 (19.4%) Asian/Asian American: 50 (22.5) Other: 2 (0.9%)	Freshman: 36 (16.2%) Sophomore: 75 (33.8%) Junior: 69 (31.1%) Senior: 41 (18.5%)	NR	NR	NR	Psychiatric History: 42 (18.9%)Anxiety: 25 (59.5% of those with history) Depression: 21 (50.0% of those with history) OCD: 2 (4.8%)ADHD: 1 (2.4%)Panic Disorder: 1 (2.4%)	NR	Nursing: 90 (40.5%) Biomedical Sciences: 37 (16.7%) Public Health: 39 (17.6%) Exercise Science: 29 (13.1%) Nutritional Science: 21 (9.5%) Pharmaceutical Sciences: 6 (2.7%)
Lee et al	2024	18–30	477 (35.1%)	Hong Kong SAR, China: 1359 (100%)	NR	NR	NR	NR	99 (7.3%)	NR	NR
Li et al. (A)	2024	18–30	12,440 (40.8%)	Eastern China: 10,184 (33.4%) Central China: 14,985 (49.2%)Western China: 5306 (17.4%)	Freshman: 9,718 (31.9%) Sophomore: 11,941 (39.2%) Junior: 6406 (21.0%) Senior: 2410 (7.9%)	NR	NR	NR	NR	NR	NR
Li et al. (B)	2024	20.39 (1.33)	2447 (31.2%)	Daqing City, Heilongjiang Province: 7845 (100%)	Junior College: 366 (4.7%) Bachelor's Degree: 7479 (95.3%)	NR	NR	NR	NR	NR	NR
Li et al. (C)	2024	20.24 (1.11)	4996 (66.8%)	Rural: 5780 (77.2%) Urban: 1704 (22.8%)	Junior College: 7354 (98.3%) Undergraduate: 130 (1.7%)	Poverty Students: 1372 (18.3%) Non-Poverty: 6112 (81.7%)	NR	NR	NR	NR	NR
Liu et al	2024	NR	631 (30.5%)	Rural: 1359 (65.8%) Urban: 707 (34.2%) Ethnicity Han: 1575 (76.2%) Minorities: 491 (23.8%)	Freshman: 232 (11.2%) Sophomore: 296 (14.3%) Junior: 626 (30.3%) Senior: 256 (12.4%) Fifth: 9 (0.4%) Graduate: 647 (31.3%)	Monthly Household Income ≤ 5000 RMB: 1164 (56.3%) 5000–8000 RMB: 452 (21.9%) > 8000 RMB: 450 (21.8%) Expenditure per month < 500 RMB: 62 (3.0%) 500–999 RMB: 469 (22.7%) 1000–1499 RMB: 963 (46.6%) 1500–1999 RMB: 340 (16.5%) ≥ 2000 RMB: 232 (11.2%)	NR	NR	NR	NR	Non-medical: 777 (37.6%) Medical: 1289 (62.4%)
Makki et al	2024	21.4 (1.9)	136 (26.2%)	Medina, Saudi Arabia: 520 (100%)	NR	NR	NR	NR	87 (16.7%)	Single: 495 (95.2%) Married: 23 (4.4%) Divorced: 2 (0.4%)	Health-Related: 268 (51.5%) Non-Health-Related: 252 (48.5%)
Martínez-Esquivel et al	2024	19.43 (1.75)	38 (50.0%)	Province of Domicile: Puntarenas: 29 (38.2%) San José: 25 (32.9%) Limón: 13 (17.1%) Other: 9 (11.8%)	Integrated General Studies (early years): 76 (100%)	NR	NR	NR	NR	Single: 72 (94.7%) Free Union: 4 (5.3%)	Multi-disciplinary (General Studies): 76 (100%)
Milić et al	2024	22 (20–26)*	292 (13.7%)	Urban: 1451 (67.9%) Rural: 686 (32.1%)	NR	Excellent (5): 183 (8.6%) Good (4): 826 (38.7%) Average (3): 1007 (47.1%) Low (2): 105 (4.9%) Very Low (1): 16 (0.7%)	Employed (Medical): 821 (38.4%) Employed (Other): 424 (19.8%)	NR	NR	NR	Health Sciences: Nursing: 1479 (69.2%) Physiotherapy: 332 (15.5%) Radiological Tech: 101 (4.7%) Sanitary Eng.: 68 (3.2%) Medical Lab: 74 (3.5%) Midwifery: 44 (2.1%) Occupational Therapy: 33 (1.5%) Other: 6 (0.3%)
Murthy et al	2024	20.03 (1.56)	71 (31.8%)	Chengalpattu District, Tamil Nadu, India: 223 (100%)	NR	NR	NR	NR	NR	NR	Engineering: 223 (100%)
Saqib & Gazerani	2024	18–21 years: 47 (36%) 22–25 years: 76 (57%) 26–29 years: 9 (7%)	26 (20%)	NR	NR	NR	NR	NR	NR	NR	Medicine and Health Sciences: 38 (29%) Other Fields: 94 (71%)
Simionescu et al	2024	19 years old: 119 (28.2%) 20 years old: 167 (39.6%) 21 years old: 84 (19.9%) 22 years old: 52 (12.3%)	255 (60.4%)	NR	NR	NR	NR	NR	NR	NR	NR
Su et al	2024	NR	NR	All participants were located in Shaanxi Province, China, under the Xi'an health lockdown	NR	NR	NR	NR	NR	NR	Shaanxi University of Traditional Chinese Medicine: 1089 (82.1%) Xi'an Medical College: 238 (17.9%)
Sun et al	2024	18–20	236 (47.4%)	Rural: 207 (41.6%) Urban: 291 (58.4%)	NR	NR	NR	NR	NR	NR	NR
Wang et al	2024	18.9 (0.84)	2312 (30.4%)	Rural: 4,100 (53.8%) Urban: 3,517 (46.2%)	All participants: First-year college students	Low: 1,978 (26.0%) Medium: 5,252 (69.0%) High: 387 (5.1%)	NR	NR	NR	NR	NR
Wu et al	2024	NR	497 (39.6%)	Hangzhou city: 184 (14.7%) Zhejiang province (non-Hangzhou): 764 (60.9%) Other provinces: 307 (24.5%)	Freshman: 670 (53.4%) Sophomore: 393 (31.3%) Junior: 192 (15.3%)	Poor: 188 (15.0%) Middle: 946 (75.4%) Rich: 121 (9.6%)	NR	NR	NR	NR	Liberal arts: 175 (14.0%) Economy: 776 (61.8%) Science: 304 (24.2%)
Xin et al	2024	19 (18–21)	1096 (25.8%)	Nationwide (China): (100%)	NR	NR	NR	NR	126 (3.0%)	NR	NR
Xu et al	2024	20.07 (1.63)	5931 (31.9%)	China: (100%)	NR	NR	NR	226 (1.2%)	NR	NR	NR
Yaghmour et al	2024	NR	NR	All participants were at King Abdulaziz University, Jeddah, Saudi Arabia	NR	NR	NR	NR	73 (19.1%) had a previous diagnosis of a mental illness	NR	All participants were medical students
Alageel et al	2023	NR	313 (72.1%)	NR	3rd: 87 (20.1%) 4th: 103 (23.7%) 5th: 114 (26.2%) 6th: 130 (30.0%)	NR	NR	NR	NR	NR	College of Medicine: 434 (100%)
Cao et al	2023	22.6 (2.5)	644 (36.4%)	China: 1770 (100%)	NR	NR	NR	NR	NR	NR	Liberal Arts: 730 (41.2%) Science: 1040 (58.8%)
Cao & Zheng	2023	17–33	3449 (73.30%)	Urban: 990 (21.04%) Rural: 3715 (78.96%)	NR	NR	NR	NR	NR	NR	Higher Vocational College: 4705 (100%)
Chen et al	2023	20.4 (19.3–21.5)*	10,527 (46.5%)	China: 22,624 (100%) (Hebei Province)	Junior College: 6,821 (30.1%) Undergraduate: 15,803 (69.9%)	NR	NR	NR	NR	NR	Medical: 2,579 (11.4%) Others: 20,045 (88.6%)
Deng & Zhang	2023	18–19: 475 (43.9%) 20–25: 608 (56.1%)	402 (37.1%)	China: 1083 (100%)	Freshman: 525 (48.5%) Sophomore: 462 (42.6%) Junior or above: 96 (8.9%)	< 3,000 RMB: 294 (27.1%) ≥ 3,000 RMB: 789 (72.9%)	NR	NR	NR	NR	Non-medical college students: 1083 (100%)
He et al	2023	41 (14)	206 (53.2%)	China: 387 (100%)	Primary school: 56 (14.5%) Junior school: 122 (31.5%) High school: 110 (28.4%) College and above: 99 (25.6%)	NR	Employed: 292 (75.5%) Unemployed: 61 (15.8%) Retirement: 34 (8.8%)	NR	NR	Single: 101 (26.1%) Married: 270 (69.8%) Divorced: 16 (4.1%)	NR
Huang & Liu	2023	19 (15–29)#	533 (45.3%)	Rural: 584 (49.7%) Urban: 592 (50.3%)	Freshman: 458 (39.0%) Sophomore: 229 (19.5%) Junior: 214 (18.2%) Senior: 275 (23.4%)	Low (< 999 CNY): 148 (12.6%) Middle (1000–1499 CNY): 352 (29.9%) High (1500–1999 CNY): 401 (34.1%) Super (≥ 2000 CNY): 275 (23.4%)	NR	NR	NR	NR	Arts & Humanities: 214 (18.2%) Science & Technology: 297 (25.3%) Medicine: 205 (17.4%) Management: 384 (32.7%) Others: 76 (6.5%)
Kavvadas et al	2023	18–25: 1138 (76%) ≥ 26: 359 (24%)	494 (33%)	Greece (Aristotle University of Thessaloniki): 1497 (100%)	Undergraduate (BSc/MD): 1108 (74%) Postgraduate (MSc/PhD): 389 (26%)	NR	NR	NR	Previous psychological/psychiatric treatment: 344 (23%) Current psychological/psychiatric treatment: 171 (11.4%) Current intake of psychoactive medication: 57 (3.8%)	unmarried/Single: 1362 (91%) Other: 135 (9%)	NR
Liu et al	2023	18.38 (0.92)	526 (22.1%)	Nanchang, Jiangxi Province, China: 2381 (100%)	NR	NR	NR	NR	NR	NR	All participants were vocational college students
Lv et al	2023	20.29 (1.51)	318 (34.75%)	Urban: 498 (54.43%) Rural: 417 (45.57%)	Freshman: 193 (21.09%) Sophomore: 267 (29.18%) Junior: 163 (17.81%) Senior + : 292 (31.91%)	NR	NR	NR	NR	NR	Literature: 213 (23.28%) Comprehensive: 190 (20.77%) Science/Engineering: 260 (28.42%) Medicine: 252 (27.54%)
Martínez-Líbano et al	2023	27.18 (8.65)	297 (28.0%)	Chilean: 1050 (98.9%) Foreign: 12 (1.1%)	University: 792 (74.6%) Technical Institute: 270 (25.4%)	NR	Study Only: 466 (43.9%) Work and Study: 596 (56.1%)	NR	Prescription Drug Use (Antidepressants/Anxiolytics): 107 (10.1%)	With Partner: 638 (60.1%) Without Partner: 424 (39.9%)	Health: 236 (22.2%) Social Sciences: 363 (34.2%) Engineering & Business: 227 (21.4%) Education: 165 (15.5%) Basic Sciences: 36 (3.4% Arts & Communications: 35 (3.3%)
Nahrin et al	2023	NR	1169 (76.8%)	Rural: 1127 (74.9%) Urban: 377 (25.1%)	NR	Monthly Family Income (BDT): < 15,000: 375 (36.5%) 15,000–30,000: 383 (37.3%) > 30,000: 270 (26.3%)	NR	NR	NR	NR	General University: 1100 (76.4%) Medical: 217 (15.1%) Engineering: 89 (6.2%) Agriculture: 33 (2.3%)
Pérez et al	2023	21 (19–24)*	1677 (24.7%)	Spanish (100%)	NR	NR	NR	NR	Anxiety: 30.8% (Never: 69.2%) Depression: 21.9% (Never: 78.1%)	With Partner: 573 (8.4%) Other categories not marital status	Education: 7.4% Language Studies: 7.1%
Qiao et al	2023	25 (24.0–27.0)*	118 (30.7%)	Shanghai, China (100%)	Undergraduate: 60 (15.6%) Postgraduate: 190 (49.6%) Doctoral student: 134 (34.9%)	NR	NR	NR	NR	Unmarried: 338 (88.0%) Married: 46 (12.0%)	NR
Rajasekhar et al	2023	20.0 (1.9)	1341 (65.9%)	Jodhpur, Rajasthan, India (100%)	NR	NR	NR	NR	NR	Unmarried: 1994 (98.0%) Ever Married: 41 (2.0%)	Health: 705 (34.6%) Engineering: 633 (31.1%) Others: 697 (34.3%)
Ramón-Arbués et al	2023	< 20: 153 (45.1%) 20–24: 134 (39.5%) ≥ 25: 52 (15.4%)	55 (16.2%)	Spain (100%)	NR	NR	NR	NR	NR	Unemployed: 263 (77.6%) Employed: 76 (22.4%)	Nursing: 339 (100%)
Xia et al	2023	NR	397 (39.1%)	China: 1016 (100%)	NR	NR	NR	NR	NR	NR	NR
Li et al	2022	< 18: 459 (6.65%) 18–25: 6446 (93.35%)	3575 (51.77%)	Lu'an City, Anhui Province, China: 6905 (100%)	Freshman (Grade 1): 4293 (62.17%) Sophomore (Grade 2): 2612 (37.83%)	NR	NR	NR	NR	NR	Medical: 2645 (38.31%) Non-medical: 4260 (61.69%)

*Abbreviations*: *SD* Standard Deviation, *N* Number, *N (%)* Number (Percentage), *NR* Not Reported, *OCD* Obsessive–Compulsive Disorder, *ADHD* Attention-Deficit/Hyperactivity Disorder, *TGNB* Transgender and Gender Non-Binary, *BSc* Bachelor of Science, *MD* Doctor of Medicine, *MSc* Master of Science, *PhD* Doctoral degree, *SAR* Special Administrative Region (e.g., Hong Kong SAR), *RMB* Renminbi (Chinese Yuan), *AED* United Arab Emirates Dirham, *CNY* Chinese Yuan, *HSC* Health Sciences Center, *CDI* Credit and Dormitory Institution, *BDT* Bangladeshi Taka

The proportion of male participants varied considerably between studies, ranging from approximately 13% to over 70%. Additionally, some studies reported a female predominance, while a minority demonstrated a more balanced or male-dominated sample. Several studies also reported gender diversity beyond the binary classification, including transgender and gender non-binary participants.

Participants were enrolled across various academic levels, including freshman through senior undergraduate students, as well as junior college, postgraduate, and doctoral students in some studies. Several studies focused exclusively on specific academic years, particularly first-year students. Regarding academic disciplines, students from health-related fields (e.g., medicine, nursing, allied health sciences) were frequently represented, either exclusively or alongside students from non-health-related faculties, such as social sciences, engineering, business, education, humanities, and basic sciences. Some studies included participants from multiple faculties, while others focused on a single discipline or institution.

Socioeconomic status was variably reported across studies using indicators such as household income, monthly allowance, or self-reported economic level. Where available, most participants were categorized within low-to-middle income brackets, although notable heterogeneity was observed across countries and regions. Employment status was reported in a subset of studies. Most participants were unemployed, consistent with full-time student status, while smaller proportions reported being employed or engaged in freelance work. A few studies specifically included participants who were both working and studying.

Where marital status was reported, most participants were single or unmarried, with only a small proportion being married or cohabiting. Family-related variables, such as family history of mental disorders, were infrequently reported, and when available, prevalence rates were generally low. Information on prior mental health conditions or treatment history was inconsistently reported. Some studies documented a history of diagnosed mental disorders, psychological or psychiatric treatment, or medication use, while many studies did not report these variables. The prevalence of previously diagnosed mental health conditions varied substantially across studies.

### Risk of bias assessment of included studies

The methodological quality of the included studies was assessed using the JBI Critical Appraisal Checklist for Analytical Cross-Sectional Studies. Overall, the included studies demonstrated moderate to high methodological quality, with most meeting most of the appraisal criteria.

Across studies, clear inclusion criteria and detailed descriptions of study subjects and settings were consistently reported, with nearly all studies receiving a “Yes” rating for these domains. Similarly, the use of objective and standard criteria for outcome measurement and appropriate statistical analysis was reported in most studies.

Regarding exposure measurement validity and reliability, most studies employed validated and reliable measurement instruments; however, several studies were rated as “No” or “Unclear,” indicating some variability in how exposure was assessed. In contrast, outcome measurement validity and reliability were generally well addressed, with most studies reporting the use of validated screening tools for mental health outcomes.

Assessment of confounding factors showed greater heterogeneity. While many studies adequately identified potential confounders, a smaller proportion clearly described strategies to address confounding, with several studies rated as “No” or “Unclear” for these domains. This suggests that residual confounding cannot be entirely excluded in some included studies.

Only a limited number of studies exhibited multiple methodological limitations, such as unclear exposure measurement, lack of confounder adjustment, or unclear outcome assessment. Nevertheless, no study was excluded based on quality assessment alone, as all were deemed sufficiently robust for inclusion in the quantitative synthesis. A detailed summary of the quality assessment results for individual studies is presented in Table 4.

**Table 4:** Quality assessment of cross-sectional studies

Author	Year	1	2	3	4	5	6	7	8
Ahmed et al	2025	Yes	Yes	Yes	Yes	Yes	Yes	Yes	Yes
Alawadhi et al	2025	No	Yes	No	Yes	Yes	Yes	Yes	Yes
Arias-Coronel et al	2025	Yes	Yes	No	Yes	No	No	Yes	Yes
Barr-Porter et al	2025	Yes	Yes	No	No	No	Yes	No	Yes
de Alencar Ribeiro et al	2025	Yes	Yes	Yes	Yes	No	No	Yes	Yes
Dubovi & Edwards	2025	Yes	Yes	Yes	Yes	No	No	Yes	Yes
Erbil et al	2025	Yes	Yes	Yes	Yes	No	No	Yes	Yes
Flesaker et al	2025	Yes	Yes	Yes	Yes	Yes	Yes	Yes	Yes
Frausing et al	2025	Yes	Yes	Yes	Yes	Yes	Yes	Yes	Yes
Han et al. (A)	2025	Yes	Yes	No	Yes	Yes	Yes	Yes	Yes
Han et al. (B)	2025	Yes	Yes	Yes	Yes	Yes	Yes	Yes	Yes
Kong et al	2025	Yes	Yes	Yes	Yes	Yes	Yes	Yes	Yes
Kunda et al	2025	Yes	Yes	No	Yes	Yes	Yes	Yes	Yes
Pratummanee et al	2025	Yes	Yes	Yes	Yes	Yes	Yes	Yes	Yes
Sudershan et al	2025	Yes	Yes	Unclear	Yes	No	Yes	Yes	Yes
Webb et al	2025	Yes	Yes	No	Yes	Yes	Yes	Yes	Yes
Xu et al	2025	Yes	Yes	Yes	Yes	Yes	Yes	Yes	Yes
Alotaibi et al	2024	Yes	Yes	Yes	Yes	Yes	Yes	Yes	Yes
Chen et al. (A)	2024	Yes	Yes	Yes	Yes	Yes	Yes	Yes	Yes
Chen et al. (B)	2024	Yes	Yes	Yes	Yes	Yes	Yes	Yes	Yes
Feng et al	2024	Yes	Yes	Yes	Yes	Yes	Yes	Yes	Yes
Gao et al	2024	Yes	Yes	Yes	Yes	Yes	Yes	Yes	Yes
Govindan et al	2024	Yes	Yes	Yes	Yes	Unclear	Unclear	Yes	Yes
Jiang et al	2024	Yes	Yes	Yes	Yes	Unclear	Yes	Yes	Yes
Kharroubi et al	2024	Yes	Yes	Yes	Yes	Yes	Yes	Yes	Yes
Kilic et al	2024	Yes	Yes	Yes	Yes	Unclear	Unclear	Yes	Yes
Lai et al	2024	Yes	Yes	Yes	Yes	Unclear	Yes	Yes	Yes
Lee et al	2024	Yes	Yes	Yes	Yes	Unclear	Unclear	Yes	Yes
Li et al. (A)	2024	Yes	Yes	Yes	Yes	Unclear	Yes	Yes	Yes
Li et al. (B)	2024	Yes	Yes	Yes	Yes	Unclear	No	Yes	Yes
Li et al. (C)	2024	Yes	Yes	Yes	Yes	Yes	Yes	Yes	Yes
Liu et al	2024	Yes	Yes	Yes	Yes	Yes	Yes	Yes	Yes
Makki et al	2024	Yes	Yes	Unclear	Yes	Yes	No	Yes	Yes
Martínez-Esquivel et al	2024	Yes	Yes	Yes	Yes	Yes	Yes	Yes	Yes
Milić et al	2024	Yes	Yes	Yes	Yes	Yes	Yes	Yes	Yes
Murthy et al	2024	Yes	Yes	Unclear	Unclear	No	No	Unclear	No
Saqib & Gazerani	2024	Yes	Yes	Unclear	Yes	Yes	Yes	Yes	Yes
Simionescu et al	2024	Yes	Yes	Yes	Yes	Yes	Yes	Yes	Yes
Su et al	2024	Yes	Yes	Yes	Yes	Yes	Yes	Yes	Yes
Sun et al	2024	Yes	Yes	Yes	Yes	Unclear	No	Yes	Yes
Wang et al	2024	Yes	Yes	Yes	Yes	Yes	Yes	Yes	Yes
Wu et al	2024	Yes	Yes	Yes	Yes	Yes	Yes	Yes	Yes
Xin et al	2024	Yes	Yes	Yes	Yes	Yes	Yes	Yes	Yes
Xu et al	2024	Yes	Yes	Yes	Yes	Yes	Yes	Yes	Yes
Yaghmour et al	2024	Yes	Yes	Yes	Yes	No	No	Yes	Yes
Alaqeel et al	2023	Yes	Yes	Yes	Yes	No	No	Yes	Yes
Cao et al	2023	Yes	Yes	Yes	Yes	Yes	Yes	Unclear	Yes
Cao & Zheng	2023	Yes	Yes	Yes	Unclear	Yes	Yes	Unclear	Yes
Chen et al	2023	Yes	Yes	Yes	Yes	Yes	Yes	Yes	Yes
Deng & Zhang	2023	Yes	Yes	Yes	Yes	Yes	Yes	Yes	Yes
He et al	2023	Yes	Yes	Yes	Yes	Yes	Yes	Yes	Yes
Huang & Liu	2023	Yes	Yes	Yes	Yes	Yes	Yes	Yes	Yes
Kavvadas et al	2023	Yes	Yes	Yes	Yes	Yes	Yes	Yes	Yes
Liu et al	2023	Yes	Yes	Yes	Yes	Yes	Yes	Yes	Yes
Lv et al	2023	Yes	Yes	Yes	Yes	Yes	Unclear	Yes	Yes
Martínez-Líbano et al	2023	Yes	Yes	Yes	Yes	Yes	Yes	Yes	Yes
Nahrin et al	2023	Yes	Yes	Yes	Yes	Yes	No	Yes	Yes
Pérez et al	2023	Yes	Yes	Yes	Yes	Yes	Yes	Yes	Yes
Qiao et al	2023	Yes	Yes	Yes	Yes	Yes	Yes	Yes	Yes
Rajasekhar et al	2023	Yes	Yes	Yes	Yes	Yes	Yes	Yes	Yes
Ramón-Arbués et al	2023	Yes	Yes	Yes	Yes	Yes	Yes	Yes	Yes
Xia et al	2023	Yes	Yes	Yes	Yes	Yes	Yes	Yes	Yes
Li et al	2022	Yes	Yes	Yes	Yes	Yes	Yes	Yes	Yes

1. Were the criteria for inclusion in the sample clearly defined? 2. Were the study subjects and the setting described in detail? 3. Was the exposure measured in a valid and reliable way? 4. Were objective, standard criteria used for measurement of the condition? 5. Were confounding factors identified? 6. Were strategies to deal with confounding factors stated? 7. Were the outcomes measured in a valid and reliable way? 8. Was appropriate statistical analysis used?

### Pooled prevalence estimates

#### Depression outcomes

**Fig. 2:**
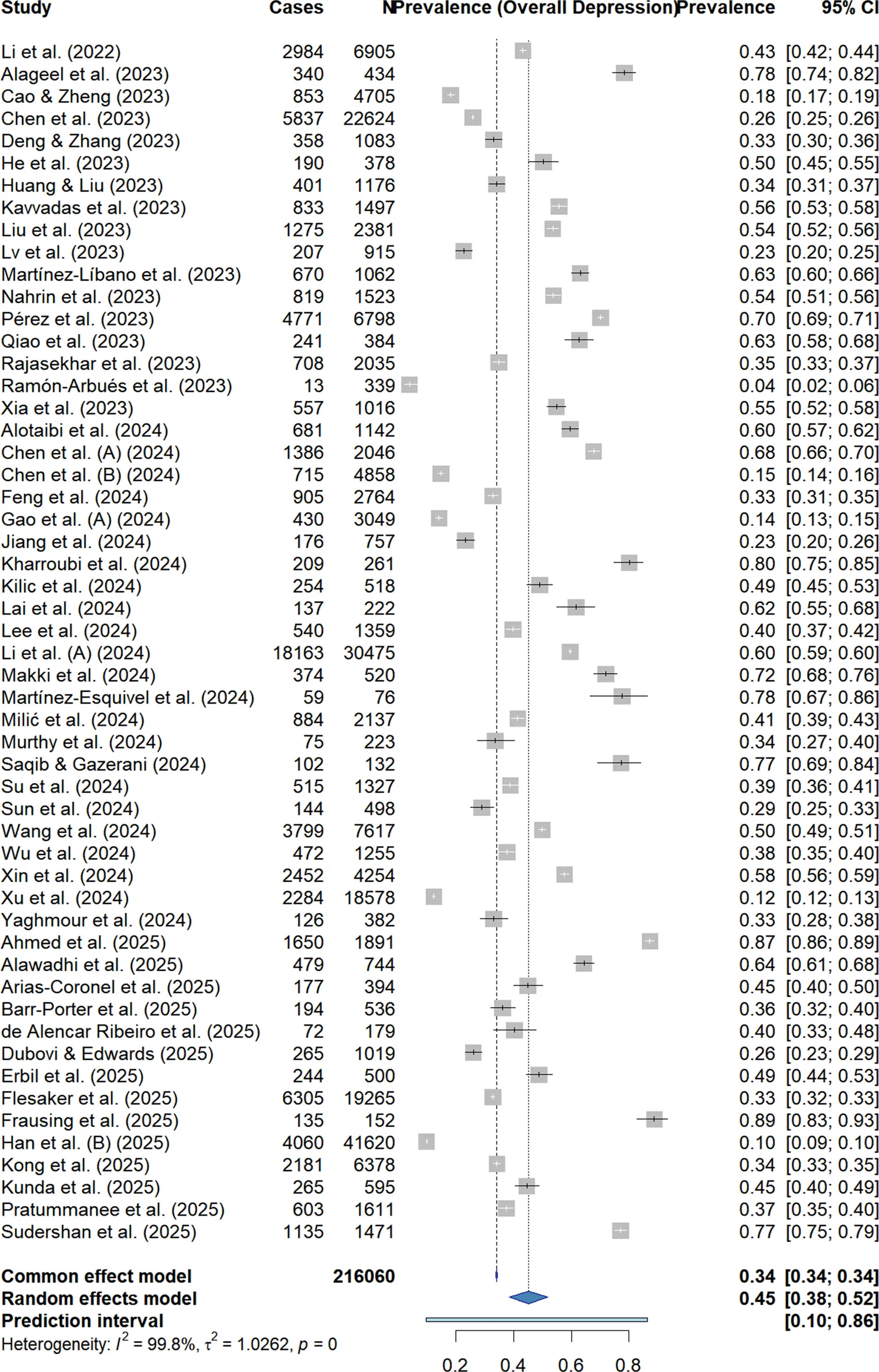
Forest plot showing the pooled prevalence of depression. CI, confidence interval; I2, heterogeneity index. * Random-effects results are the primary focus due to substantial heterogeneity

Overall Depression: 54 studies (216,060 participants) reported overall depression. Using the random-effects model (I-squared = 99.9%, tau-squared = 1.0360, Q = 34,258.62, *p* < 0.001), the pooled prevalence was 45.1% (95% CI: 38.5% to 51.8%) (Fig. 2). More details were provided in Table 5.

**Table 5:** Summary of pooled prevalence estimates

Outcome	N Studies	Total N	Prevalence	95% CI	I-squared	Model
Overall Depression	54	216,060	45.1%	38.5–51.8%	99.9%	Random
Overall Anxiety	54	200,113	43.6%	35.3–52.3%	99.9%	Random
Overall Stress	26	71,182	45.6%	32.9–58.8%	99.9%	Random

#### Anxiety outcomes

**Fig. 3:**
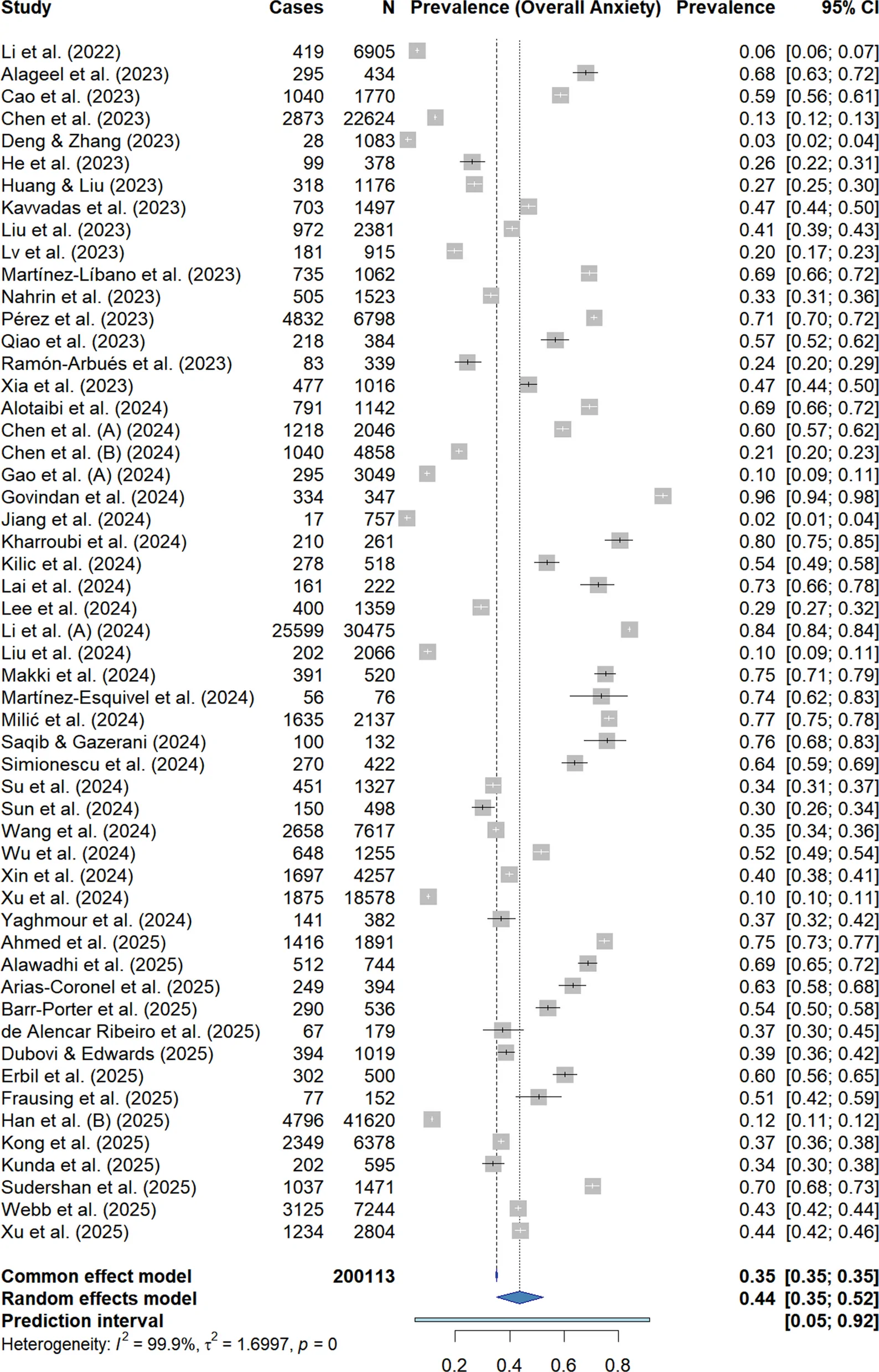
Forest plot showing the pooled prevalence of anxiety. CI, confidence interval; I2, heterogeneity index. * Random-effects results are the primary focus due to substantial heterogeneity

Overall Anxiety: 54 studies (200,113 participants). Using the random-effects model (I-squared = 99.9%, tau-squared = 1.7179, Q = 52,468.94, *p* < 0.001), the pooled prevalence was 43.6% (95% CI: 35.3% to 52.3%) (Fig. 3). More details were provided in Table 5.

### Stress outcomes

**Fig. 4:**
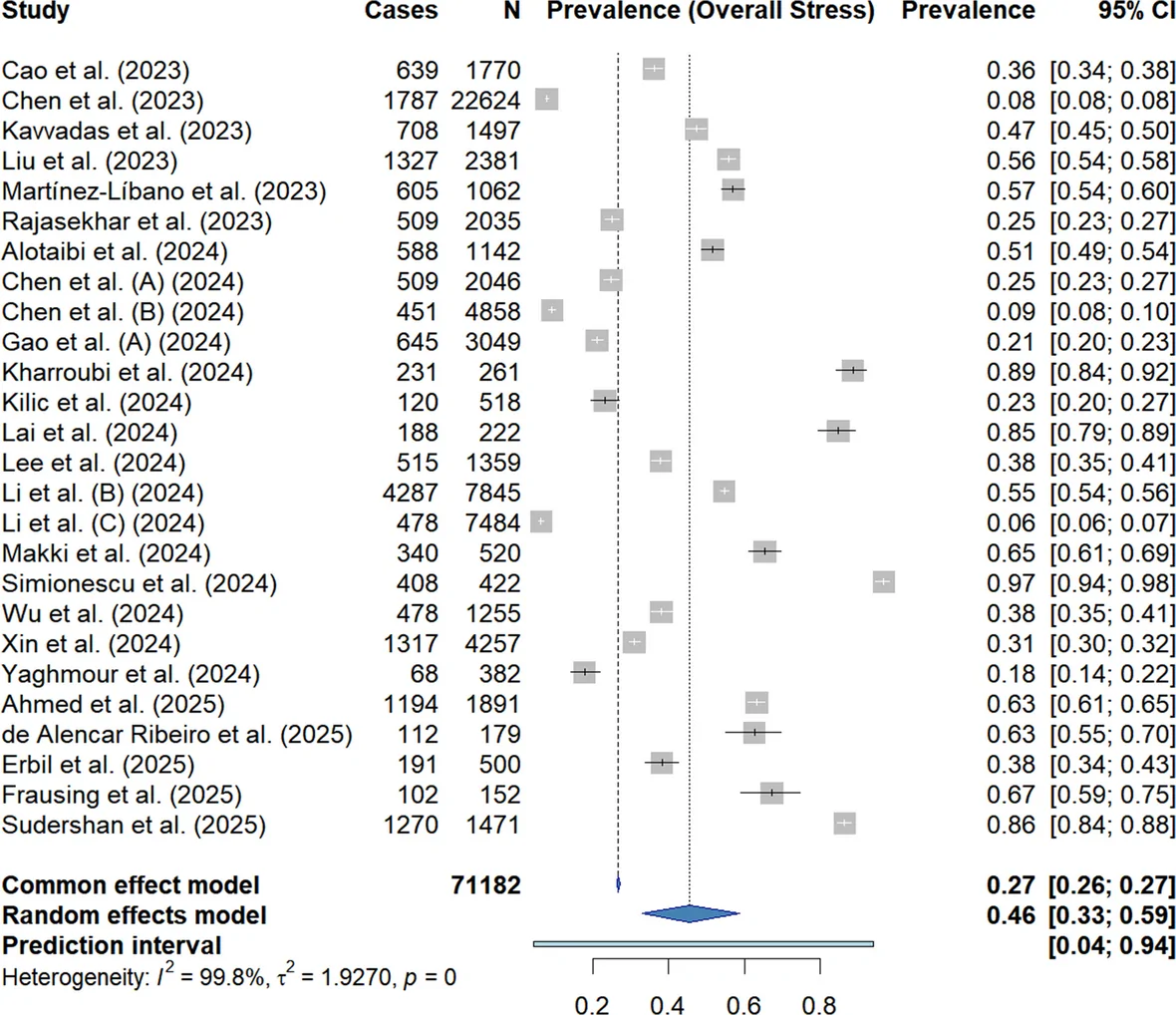
Forest plot showing the pooled prevalence of stress. CI, confidence interval; I2, heterogeneity index. * Random-effects results are the primary focus due to substantial heterogeneity

Overall Stress: 26 studies (71,182 participants). Using the random-effects model (I-squared = 99.9%, tau-squared = 1.9891, Q = 13,558.06, *p* < 0.001), the pooled prevalence was 45.6% (95% CI: 32.9% to 58.8%) (Fig. 4). More details were provided in Table 5.

### Severity-specific analyses

Severity-specific analyses demonstrated varying prevalence estimates across different levels of depression severity. Mild depression was assessed in 32 studies comprising 118,838 participants, yielding a pooled prevalence of 21.9% (95% CI: 17.8%–26.7%). A high degree of heterogeneity was observed among studies (I2 = 99.7%).

Moderate depression was reported in 31 studies including 86,736 participants. The pooled prevalence was estimated at 12.8% (95% CI: 10.0%–16.3%), with substantial heterogeneity across studies (I2 = 99.2%).

Severe depression was evaluated in 32 studies involving 114,967 participants, with a pooled prevalence of 4.1% (95% CI: 2.2%–7.6%). Heterogeneity remained considerably high in this analysis as well (I2 = 99.8%). More details were provided in Table 6 and Supplementary file 1.

**Table 6:** Summary of pooled prevalence estimates of severity specific analyses

Outcome	N Studies	Total N	Prevalence	95% CI	I-squared	Model
Mild Depression	32	118,838	21.9%	17.8–26.7%	99.7%	Random
Moderate Depression	31	86,736	12.8%	10.0–16.3%	99.2%	Random
Severe Depression	32	114,967	4.1%	2.2–7.6%	99.8%	Random
Mild Anxiety	33	112,450	23.1%	18.1–28.9%	99.8%	Random
Moderate Anxiety	32	81,646	13.7%	9.5–19.2%	99.7%	Random
Severe Anxiety	33	103,709	6.1%	3.0–11.9%	99.9%	Random
High Stress	19	46,998	14.5%	6.9–27.8%	99.8%	Random

*CI* Confidence interval

Severity-stratified analyses demonstrated a decreasing prevalence with increasing anxiety severity. Mild anxiety was reported in 33 studies comprising 112,450 participants, with a pooled prevalence of 23.1% (95% CI: 18.1%–28.9%; I2 = 99.8%). Moderate anxiety was assessed in 32 studies including 81,646 participants and showed a pooled prevalence of 13.7% (95% CI: 9.5%–19.2%; I2 = 99.7%). Severe anxiety was reported across 33 studies involving 103,709 participants, with a pooled prevalence of 6.1% (95% CI: 3.0%–11.9%; I2 = 99.9%). All analyses were conducted using random-effects models with REML estimation and showed considerable between-study heterogeneity (I2 ≥ 99.7% in all three subgroups), consistent with the heterogeneity observed in the overall pooled estimates. More details were provided in Table 6 and Supplementary file 1.

**Fig. 5:**
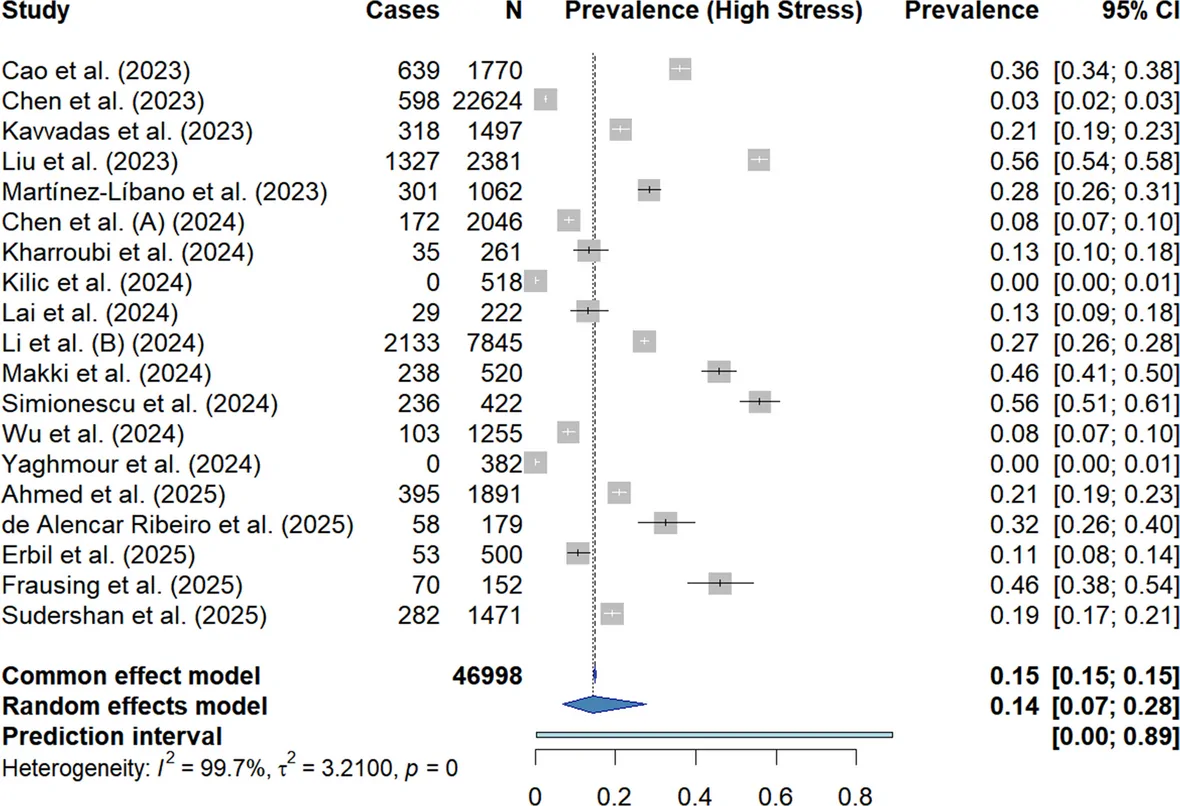
Forest plot showing the pooled prevalence of high perceived stress. CI, confidence interval; I2, heterogeneity index. * Random-effects results are the primary focus due to substantial heterogeneity

High Perceived Stress: 19 studies (46,998 participants). Pooled prevalence: 14.5% (95% CI: 6.9% to 27.8%). I-squared = 99.8% (Fig. 5). More details were provided in Table 6.

### Subgroup analysis by geographic region

For Overall Depression, subgroup analysis by geographic region showed: Africa (70.1%, 95% CI: 22.3%−95.0%, *n* = 2); Middle East (63.3%, 95% CI: 49.9%−75.0%, *n* = 7); South America (56.8%, 95% CI: 39.3%−72.7%, *n* = 4); North America (38.4%, 95% CI: 24.8%−54.1%, *n* = 4); Asia (37.8%, 95% CI: 31.2%−45.0%, *n* = 29); Other (73.1%, 95% CI: 25.4%−95.6%, *n* = 2); Europe (44.0%, 95% CI: 14.6%−78.3%, *n* = 5). The test for subgroup interaction was statistically significant (*p* = 0.026). More details were provided in Table 7 and Supplementary file 1.

**Table 7:** Subgroup analysis by geographic region

Outcome	Region	N	Prevalence	95% CI	I-squared	p-interaction
Overall Depression	Africa	2	70.1%	22.3–95.0%	99.7%	0.026
	Middle East	7	63.3%	49.9–75.0%	98.5%	0.026
	South America	4	56.8%	39.3–72.7%	97.3%	0.026
	North America	4	38.4%	24.8–54.1%	99.0%	0.026
	Asia	29	37.8%	31.2–45.0%	99.8%	0.026
	Other	2	73.1%	25.4–95.6%	98.3%	0.026
	Europe	5	44.0%	14.6–78.3%	99.9%	0.026
Overall Anxiety	Africa	2	55.3%	18.1–87.4%	99.7%	0.001
	Middle East	8	62.8%	51.3–72.9%	98.9%	0.001
	South America	4	61.3%	44.9–75.4%	96.8%	0.001
	North America	3	55.4%	35.6–73.6%	98.3%	0.001
	Asia	27	28.5%	19.8–39.1%	99.9%	0.001
	Other	4	74.1%	40.9–92.2%	99.2%	0.001
	Europe	5	59.7%	38.2–78.0%	99.7%	0.001
Overall Stress	Middle East	5	49.9%	22.1–77.9%	99.5%	0.236
	Asia	12	30.8%	17.8–47.8%	99.9%	0.236
	South America	2	58.8%	53.5–63.8%	49.1%	0.236
	Other	3	76.7%	26.2–96.8%	99.3%	0.236
Mild Depression	Middle East	5	26.4%	18.4–36.3%	95.4%	0.231
	South America	4	20.5%	10.4–36.3%	96.7%	0.231
	Asia	14	25.5%	20.0–31.9%	99.7%	0.231
	North America	2	8.2%	0.7–54.5%	99.6%	0.231
	Europe	5	18.4%	10.5–30.3%	99.1%	0.231
Moderate Depression	Middle East	4	19.2%	15.3–23.8%	78.6%	0.007
	South America	4	20.2%	16.3–24.8%	69.5%	0.007
	Asia	14	9.2%	6.3–13.2%	99.3%	0.007
	Other	2	20.1%	17.2–23.4%	0.0%	0.007
	North America	2	4.9%	0.6–30.8%	99.1%	0.007
	Europe	4	17.6%	12.7–23.8%	97.1%	0.007
Severe Depression	Middle East	5	2.9%	0.2–26.8%	99.2%	0.186
	South America	4	13.8%	9.3–19.9%	84.6%	0.186
	Asia	14	2.5%	1.2–5.3%	99.7%	0.186
	Other	2	27.2%	1.2–91.7%	99.5%	0.186
	North America	2	1.9%	0.2–15.3%	98.2%	0.186
	Europe	4	7.8%	2.1–24.7%	99.6%	0.186
Mild Anxiety	Middle East	6	23.2%	14.9–34.3%	98.2%	0.920
	South America	4	16.8%	6.4–37.2%	97.8%	0.920
	Asia	14	21.4%	13.3–32.7%	99.9%	0.920
	Other	2	30.6%	11.4–60.2%	98.4%	0.920
	Europe	5	27.6%	17.3–41.1%	99.2%	0.920
Moderate Anxiety	Middle East	5	19.2%	12.8–27.9%	97.1%	0.304
	South America	4	17.4%	15.7–19.3%	0.0%	0.304
	Asia	14	7.8%	3.6–16.2%	99.8%	0.304
	Other	3	25.4%	15.3–39.2%	94.3%	0.304
	Europe	4	19.4%	12.4–29.1%	98.7%	0.304
Severe Anxiety	Middle East	6	9.7%	2.2–33.7%	99.6%	0.036
	South America	4	17.9%	7.0–38.9%	97.4%	0.036
	Asia	14	2.1%	0.7–6.0%	99.8%	0.036
	Other	3	21.2%	16.0–27.5%	77.3%	0.036
	Europe	4	20.3%	9.3–38.6%	99.6%	0.036
High Stress	Asia	7	16.9%	7.2–34.7%	99.9%	0.674
	South America	2	29.2%	26.1–32.5%	18.4%	0.674
	Other	3	33.6%	10.7–68.0%	98.9%	0.674
	Middle East	4	2.5%	0.1–41.9%	99.4%	0.674

p-interaction: *p*-value for test of subgroup differences

For Mild Depression, subgroup analysis by geographic region showed: Middle East (26.4%, 95% CI: 18.4%−36.3%, *n* = 5); South America (20.5%, 95% CI: 10.4%−36.3%, *n* = 4); Asia (25.5%, 95% CI: 20.0%−31.9%, *n* = 14); North America (8.2%, 95% CI: 0.7%−54.5%, *n* = 2); Europe (18.4%, 95% CI: 10.5%−30.3%, *n* = 5). The test for subgroup interaction was not statistically significant (*p* = 0.231). More details were provided in Table 7 and Supplementary file 1.

For Moderate Depression, subgroup analysis by geographic region showed: Middle East (19.2%, 95% CI: 15.3%−23.8%, *n* = 4); South America (20.2%, 95% CI: 16.3%−24.8%, *n* = 4); Asia (9.2%, 95% CI: 6.3%−13.2%, *n* = 14); Other (20.1%, 95% CI: 17.2%−23.4%, *n* = 2); North America (4.9%, 95% CI: 0.6%−30.8%, *n* = 2); Europe (17.6%, 95% CI: 12.7%−23.8%, *n* = 4). The test for subgroup interaction was statistically significant (*p* = 0.007). More details were provided in Table 7 and Supplementary file 1.

For Severe Depression, subgroup analysis by geographic region showed: Middle East (2.9%, 95% CI: 0.2%−26.8%, *n* = 5); South America (13.8%, 95% CI: 9.3%−19.9%, *n* = 4); Asia (2.5%, 95% CI: 1.2%−5.3%, *n* = 14); Other (27.2%, 95% CI: 1.2%−91.7%, *n* = 2); North America (1.9%, 95% CI: 0.2%−15.3%, *n* = 2); Europe (7.8%, 95% CI: 2.1%−24.7%, *n* = 4). The test for subgroup interaction was not statistically significant (*p* = 0.186). More details were provided in Table 7 and Supplementary file 1.

For Overall Anxiety, subgroup analysis by geographic region showed: Africa (55.3%, 95% CI: 18.1%−87.4%, *n* = 2); Middle East (62.8%, 95% CI: 51.3%−72.9%, *n* = 8); South America (61.3%, 95% CI: 44.9%−75.4%, *n* = 4); North America (55.4%, 95% CI: 35.6%−73.6%, *n* = 3); Asia (28.5%, 95% CI: 19.8%−39.1%, *n* = 27); Other (74.1%, 95% CI: 40.9%−92.2%, *n* = 4); Europe (59.7%, 95% CI: 38.2%−78.0%, *n* = 5). The test for subgroup interaction was statistically significant (*p* = 0.001). More details were provided in Table 7 and Supplementary file 1.

For Mild Anxiety, subgroup analysis by geographic region showed: Middle East (23.2%, 95% CI: 14.9%−34.3%, *n* = 6); South America (16.8%, 95% CI: 6.4%−37.2%, *n* = 4); Asia (21.4%, 95% CI: 13.3%−32.7%, *n* = 14); Other (30.6%, 95% CI: 11.4%−60.2%, *n* = 2); Europe (27.6%, 95% CI: 17.3%−41.1%, *n* = 5). The test for subgroup interaction was not statistically significant (*p* = 0.920). More details were provided in Table 7 and Supplementary file 1.

For Moderate Anxiety, subgroup analysis by geographic region showed: Middle East (19.2%, 95% CI: 12.8%−27.9%, *n* = 5); South America (17.4%, 95% CI: 15.7%−19.3%, *n* = 4); Asia (7.8%, 95% CI: 3.6%−16.2%, *n* = 14); Other (25.4%, 95% CI: 15.3%−39.2%, *n* = 3); Europe (19.4%, 95% CI: 12.4%−29.1%, *n* = 4). The test for subgroup interaction was not statistically significant (*p* = 0.304). More details were provided in Table 7 and Supplementary file 1.

For Severe Anxiety, subgroup analysis by geographic region showed: Middle East (9.7%, 95% CI: 2.2%−33.7%, *n* = 6); South America (17.9%, 95% CI: 7.0%−38.9%, *n* = 4); Asia (2.1%, 95% CI: 0.7%−6.0%, *n* = 14); Other (21.2%, 95% CI: 16.0%−27.5%, *n* = 3); Europe (20.3%, 95% CI: 9.3%−38.6%, *n* = 4). The test for subgroup interaction was statistically significant (*p* = 0.036). More details were provided in Table 7 and Supplementary file 1.

For Overall Stress, subgroup analysis by geographic region showed: Middle East (49.9%, 95% CI: 22.1%−77.9%, *n* = 5); Asia (30.8%, 95% CI: 17.8%−47.8%, *n* = 12); South America (58.8%, 95% CI: 53.5%−63.8%, *n* = 2); Other (76.7%, 95% CI: 26.2%−96.8%, *n* = 3). The test for subgroup interaction was not statistically significant (*p* = 0.236). More details were provided in Table 7 and Supplementary file 1.

For High Stress, subgroup analysis by geographic region showed: Asia (16.9%, 95% CI: 7.2%−34.7%, *n* = 7); South America (29.2%, 95% CI: 26.1%−32.5%, *n* = 2); Other (33.6%, 95% CI: 10.7%−68.0%, *n* = 3); Middle East (2.5%, 95% CI: 0.1%−41.9%, *n* = 4). The test for subgroup interaction was not statistically significant (*p* = 0.674). More details were provided in Table 7 and Supplementary file 1.

### Subgroup analysis by measurement screening instruments

To address potential heterogeneity arising from the use of different screening instruments with varying psychometric properties and cut-off thresholds, subgroup analyses were stratified by instrument type.

For Overall Depression, subgroup analysis by measurement instrument showed: PHQ-9 (50.4%, 95% CI: 42.5%−58.4%, *n* = 29); DASS-21 (47.1%, 95% CI: 36.9%−57.5%, *n* = 12); Other (31.3%, 95% CI: 8.0%−70.6%, *n* = 5); BDI (56.1%, 95% CI: 15.7%−89.7%, *n* = 2); CES-D (26.5%, 95% CI: 7.5%−61.6%, *n* = 3); SDS (32.5%, 95% CI: 16.3%−54.3%, *n* = 2). The test for subgroup interaction was not statistically significant (*p* = 0.298). More details were provided in Table 8 and Supplementary file 1.

**Table 8:** Subgroup analysis by measurement instrument

Outcome	Instrument	N	Prevalence	95% CI	I-squared	p-interaction
Overall Depression	PHQ-9	29	50.4%	42.5–58.4%	99.8%	0.298
	DASS-21	12	47.1%	36.9–57.5%	99.2%	0.298
	Other	5	31.3%	8.0–70.6%	99.8%	0.298
	BDI	2	56.1%	15.7–89.7%	98.0%	0.298
	CES-D	3	26.5%	7.5–61.6%	100.0%	0.298
	SDS	2	32.5%	16.3–54.3%	99.1%	0.298
Overall Anxiety	GAD-7	29	45.6%	35.9–55.6%	99.9%	< 0.001
	DASS-21	11	53.8%	43.0–64.3%	99.1%	< 0.001
	Other	7	64.9%	41.8–82.6%	99.5%	< 0.001
	SAS	4	5.4%	1.9–14.2%	99.1%	< 0.001
	HADS	2	36.5%	15.7–64.0%	96.8%	< 0.001
Overall Stress	PSS	7	71.5%	55.0–83.7%	99.5%	0.041
	DASS-21	11	37.3%	25.7–50.6%	99.4%	0.041
	Other	7	35.9%	11.3–71.2%	100.0%	0.041
Mild Depression	PHQ-9	18	24.0%	17.9–31.3%	99.6%	0.094
	Other	2	11.3%	6.9–18.0%	87.3%	0.094
	DASS-21	8	16.5%	12.5–21.4%	95.0%	0.094
	BDI	2	25.8%	9.6–53.3%	96.1%	0.094
	SDS	1	36.9%	35.8–38.0%	–%	0.094
	CES-D	1	49.6%	49.0–50.2%	–%	0.094
Moderate Depression	PHQ-9	18	13.2%	9.2–18.6%	99.5%	0.324
	Other	3	6.7%	1.9–21.0%	98.6%	0.324
	DASS-21	7	16.6%	13.9–19.6%	86.4%	0.324
	BDI	2	15.2%	8.6–25.7%	81.9%	0.324
	SDS	1	6.2%	5.7–6.8%	–%	0.324
Severe Depression	PHQ-9	18	4.6%	2.7–7.6%	99.3%	0.211
	Other	3	7.2%	0.3–66.4%	99.6%	0.211
	DASS-21	8	5.0%	1.1–19.6%	99.6%	0.211
	SDS	1	0.1%	0.0–0.2%	–%	0.211
	CES-D	1	10.0%	9.7–10.3%	–%	0.211
	BDI	1	17.1%	10.2–27.3%	–%	0.211
Mild Anxiety	GAD-7	19	30.4%	24.8–36.6%	99.7%	< 0.001
	Other	3	38.6%	29.8–48.2%	84.1%	< 0.001
	DASS-21	8	13.7%	10.2–18.1%	94.8%	< 0.001
	SAS	2	3.3%	1.1–9.7%	96.0%	< 0.001
	HADS	1	28.9%	24.3–34.0%	–%	< 0.001
Moderate Anxiety	GAD-7	19	15.0%	10.8–20.4%	99.6%	< 0.001
	Other	3	25.4%	16.5–37.1%	89.7%	< 0.001
	DASS-21	7	17.7%	13.1–23.5%	95.7%	< 0.001
	SAS	2	0.4%	0.3–0.5%	0.0%	< 0.001
	HADS	1	29.6%	22.9–37.3%	–%	< 0.001
Severe Anxiety	GAD-7	19	6.7%	3.6–12.3%	99.8%	< 0.001
	Other	3	12.6%	5.9–25.0%	92.0%	< 0.001
	DASS-21	8	16.2%	5.7–38.1%	99.6%	< 0.001
	SAS	2	0.1%	0.0–1.0%	87.3%	< 0.001
	HADS	1	21.1%	15.3–28.3%	–%	< 0.001
High Stress	PSS	7	27.0%	16.5–40.8%	99.3%	0.371
	Other	4	15.6%	3.5–48.8%	99.9%	0.371
	DASS-21	8	8.9%	2.1–31.5%	99.7%	0.371

For Mild Depression, subgroup analysis by measurement instrument showed: PHQ-9 (24.0%, 95% CI: 17.9%−31.3%, *n* = 18); Other (11.3%, 95% CI: 6.9%−18.0%, *n* = 2); DASS-21 (16.5%, 95% CI: 12.5%−21.4%, *n* = 8); BDI (25.8%, 95% CI: 9.6%−53.3%, *n* = 2); SDS (36.9%, 95% CI: 35.8%−38.0%, *n* = 1); CES-D (49.6%, 95% CI: 49.0%−50.2%, *n* = 1). The test for subgroup interaction was not statistically significant (*p* = 0.094). More details were provided in Table 8 and Supplementary file 1.

For Moderate Depression, subgroup analysis by measurement instrument showed: PHQ-9 (13.2%, 95% CI: 9.2%−18.6%, *n* = 18); Other (6.7%, 95% CI: 1.9%−21.0%, *n* = 3); DASS-21 (16.6%, 95% CI: 13.9%−19.6%, *n* = 7); BDI (15.2%, 95% CI: 8.6%−25.7%, *n* = 2); SDS (6.2%, 95% CI: 5.7%−6.8%, *n* = 1). The test for subgroup interaction was not statistically significant (*p* = 0.324). More details were provided in Table 8 and Supplementary file 1.

For Severe Depression, subgroup analysis by measurement instrument showed: PHQ-9 (4.6%, 95% CI: 2.7%−7.6%, *n* = 18); Other (7.2%, 95% CI: 0.3%−66.4%, *n* = 3); DASS-21 (5.0%, 95% CI: 1.1%−19.6%, *n* = 8); SDS (0.1%, 95% CI: 0.0%−0.2%, *n* = 1); CES-D (10.0%, 95% CI: 9.7%−10.3%, *n* = 1); BDI (17.1%, 95% CI: 10.2%−27.3%, *n* = 1). The test for subgroup interaction was not statistically significant (*p* = 0.211). More details were provided in Table 8 and Supplementary file 1.

For Overall Anxiety, subgroup analysis by measurement instrument showed: GAD-7 (45.6%, 95% CI: 35.9%−55.6%, *n* = 29); DASS-21 (53.8%, 95% CI: 43.0%−64.3%, *n* = 11); Other (64.9%, 95% CI: 41.8%−82.6%, *n* = 7); SAS (5.4%, 95% CI: 1.9%−14.2%, *n* = 4); HADS (36.5%, 95% CI: 15.7%−64.0%, *n* = 2). The test for subgroup interaction was statistically significant (*p* < 0.001). More details were provided in Table 8 and Supplementary file 1.

For Mild Anxiety, subgroup analysis by measurement instrument showed: GAD-7 (30.4%, 95% CI: 24.8%−36.6%, *n* = 19); Other (38.6%, 95% CI: 29.8%−48.2%, *n* = 3); DASS-21 (13.7%, 95% CI: 10.2%−18.1%, *n* = 8); SAS (3.3%, 95% CI: 1.1%−9.7%, *n* = 2); HADS (28.9%, 95% CI: 24.3%−34.0%, *n* = 1). The test for subgroup interaction was statistically significant (*p* < 0.001). More details were provided in Table 8 and Supplementary file 1.

For Moderate Anxiety, subgroup analysis by measurement instrument showed: GAD-7 (15.0%, 95% CI: 10.8%−20.4%, *n* = 19); Other (25.4%, 95% CI: 16.5%−37.1%, *n* = 3); DASS-21 (17.7%, 95% CI: 13.1%−23.5%, *n* = 7); SAS (0.4%, 95% CI: 0.3%−0.5%, *n* = 2); HADS (29.6%, 95% CI: 22.9%−37.3%, *n* = 1). The test for subgroup interaction was statistically significant (*p* < 0.001). More details were provided in Table 8 and Supplementary file 1.

For Severe Anxiety, subgroup analysis by measurement instrument showed: GAD-7 (6.7%, 95% CI: 3.6%−12.3%, *n* = 19); Other (12.6%, 95% CI: 5.9%−25.0%, *n* = 3); DASS-21 (16.2%, 95% CI: 5.7%−38.1%, *n* = 8); SAS (0.1%, 95% CI: 0.0%−1.0%, *n* = 2); HADS (21.1%, 95% CI: 15.3%−28.3%, *n* = 1). The test for subgroup interaction was statistically significant (*p* < 0.001). More details were provided in Table 8 and Supplementary file 1.

For Overall Stress, subgroup analysis by measurement instrument showed: PSS (71.5%, 95% CI: 55.0%−83.7%, *n* = 7); DASS-21 (37.3%, 95% CI: 25.7%−50.6%, *n* = 11); Other (35.9%, 95% CI: 11.3%−71.2%, *n* = 7). The test for subgroup interaction was statistically significant (*p* = 0.041). More details were provided in Table 8 and Supplementary file 1.

For High Stress, subgroup analysis by measurement instrument showed: PSS (27.0%, 95% CI: 16.5%−40.8%, *n* = 7); Other (15.6%, 95% CI: 3.5%−48.8%, *n* = 4); DASS-21 (8.9%, 95% CI: 2.1%−31.5%, *n* = 8). The test for subgroup interaction was not statistically significant (*p* = 0.371). More details were provided in Table 8 and Supplementary file 1.

### Meta-regression analysis

Meta-regression analyses examined the association between study-level covariates and prevalence estimates. In addition to publication year and sample size, mean participant age and proportion of male participants were examined where sufficient data were available.

For Overall Depression: Publication Year was not significantly associated with prevalence (beta = 0.1113, 95% CI: −0.2361 to 0.4586, *p* = 0.530). Sample Size (log10) was significantly associated with prevalence (beta = −0.5753, 95% CI: −0.9913 to −0.1593, *p* = 0.007). Mean Age was not significantly associated with prevalence (beta = 0.0129, 95% CI: −0.0254 to 0.0513, *p* = 0.509). Male Proportion (%) was not significantly associated with prevalence (beta = −0.0057, 95% CI: −0.0253 to 0.0138, *p* = 0.565). More details were provided in Table 9 and Supplementary file 1.

**Table 9:** Meta-regression analysis results

Outcome	Covariate	N	Beta	95% CI	SE	*p*-value	R-squared
Overall Depression	Publication Year	54	0.1113	−0.2361 to 0.4586	0.1772	0.530	0.00%
	Sample Size (log10)	53	−0.5753	−0.9913 to −0.1593	0.2123	0.007	11.19%
	Mean Age	40	0.0129	−0.0254 to 0.0513	0.0196	0.509	0.00%
	Male Proportion (%)	49	−0.0057	−0.0253 to 0.0138	0.0100	0.565	0.00%
Overall Anxiety	Publication Year	54	0.3705	−0.0717 to 0.8126	0.2256	0.101	3.14%
	Sample Size (log10)	53	−0.6641	−1.2053 to −0.1230	0.2761	0.016	8.53%
	Mean Age	40	−0.0028	−0.0587 to 0.0530	0.0285	0.921	0.00%
	Male Proportion (%)	49	−0.0155	−0.0434 to 0.0124	0.0142	0.276	0.39%
Overall Stress	Publication Year	26	0.6083	−0.2098 to 1.4265	0.4174	0.145	4.38%
	Sample Size (log10)	25	−1.5540	−2.4211 to −0.6868	0.4424	< 0.001	32.43%
	Mean Age	21	0.0740	−0.1597 to 0.3078	0.1193	0.535	0.00%
	Male Proportion (%)	22	−0.0149	−0.0572 to 0.0274	0.0216	0.490	0.00%
Mild Depression	Publication Year	32	−0.0701	−0.4121 to 0.2719	0.1745	0.688	0.00%
	Sample Size (log10)	32	−0.1221	−0.5391 to 0.2949	0.2128	0.566	0.00%
	Mean Age	23	−0.0749	−0.2264 to 0.0767	0.0773	0.333	0.00%
	Male Proportion (%)	30	0.0015	−0.0180 to 0.0211	0.0100	0.877	0.00%
Moderate Depression	Publication Year	31	0.2547	−0.0851 to 0.5944	0.1733	0.142	3.74%
	Sample Size (log10)	31	−0.6163	−1.0355 to −0.1970	0.2139	0.004	20.38%
	Mean Age	23	0.0486	−0.0188 to 0.1160	0.0344	0.158	4.44%
	Male Proportion (%)	29	−0.0182	−0.0379 to 0.0014	0.0100	0.069	7.91%
Severe Depression	Publication Year	32	0.5812	−0.1152 to 1.2776	0.3553	0.102	6.24%
	Sample Size (log10)	32	−0.7291	−1.5833 to 0.1251	0.4358	0.094	8.76%
	Mean Age	24	0.1098	−0.0159 to 0.2355	0.0642	0.087	6.84%
	Male Proportion (%)	30	−0.0410	−0.0796 to −0.0024	0.0197	0.037	11.37%
Mild Anxiety	Publication Year	33	0.3382	−0.0297 to 0.7061	0.1877	0.072	6.89%
	Sample Size (log10)	33	−0.1176	−0.6012 to 0.3661	0.2467	0.634	0.00%
	Mean Age	24	−0.0032	−0.1742 to 0.1678	0.0872	0.971	0.00%
	Male Proportion (%)	31	−0.0066	−0.0336 to 0.0205	0.0138	0.635	0.00%
Moderate Anxiety	Publication Year	32	0.6485	0.2157 to 1.0813	0.2208	0.003	20.46%
	Sample Size (log10)	32	−0.5900	−1.2196 to 0.0396	0.3212	0.066	7.88%
	Mean Age	24	0.0558	−0.0314 to 0.1431	0.0445	0.210	1.84%
	Male Proportion (%)	30	−0.0291	−0.0623 to 0.0041	0.0169	0.086	6.58%
Severe Anxiety	Publication Year	33	0.7651	0.0059 to 1.5243	0.3873	0.048	8.06%
	Sample Size (log10)	33	−0.7248	−1.6832 to 0.2336	0.4890	0.138	4.82%
	Mean Age	25	0.0901	−0.0568 to 0.2370	0.0749	0.229	0.00%
	Male Proportion (%)	31	−0.0315	−0.0848 to 0.0217	0.0272	0.246	1.81%
High Stress	Publication Year	19	0.0370	−0.8575 to 0.9314	0.4564	0.935	0.00%
	Sample Size (log10)	19	−0.3804	−1.5541 to 0.7933	0.5988	0.525	0.00%
	Mean Age	15	0.0877	−0.1205 to 0.2958	0.1062	0.409	0.00%
	Male Proportion (%)	17	−0.0140	−0.0667 to 0.0388	0.0269	0.603	0.00%

Beta: change in log-odds per unit increase. R^2^: proportion of heterogeneity explained. SE: standard error of the mean

For Overall Anxiety: Publication Year was not significantly associated with prevalence (beta = 0.3705, 95% CI: −0.0717 to 0.8126, *p* = 0.101). Sample Size (log10) was significantly associated with prevalence (beta = −0.6641, 95% CI: −1.2053 to −0.1230, *p* = 0.016). Mean Age was not significantly associated with prevalence (beta = −0.0028, 95% CI: −0.0587 to 0.0530, *p* = 0.921). Male Proportion (%) was not significantly associated with prevalence (beta = −0.0155, 95% CI: −0.0434 to 0.0124, *p* = 0.276). More details were provided in Table 9 and Supplementary file 1.

For Overall Stress: Publication Year was not significantly associated with prevalence (beta = 0.6083, 95% CI: −0.2098 to 1.4265, *p* = 0.145). Sample Size (log10) was significantly associated with prevalence (beta = −1.5540, 95% CI: −2.4211 to −0.6868, *p* < 0.001). Mean Age was not significantly associated with prevalence (beta = 0.0740, 95% CI: −0.1597 to 0.3078, *p* = 0.535). Male Proportion (%) was not significantly associated with prevalence (beta = −0.0149, 95% CI: −0.0572 to 0.0274, *p* = 0.490). More details were provided in Table 9 and Supplementary file 1.

For Mild Depression: Publication Year was not significantly associated with prevalence (beta = −0.0701, 95% CI: −0.4121 to 0.2719, *p* = 0.688). Sample Size (log10) was not significantly associated with prevalence (beta = −0.1221, 95% CI: −0.5391 to 0.2949, *p* = 0.566). Mean Age was not significantly associated with prevalence (beta = −0.0749, 95% CI: −0.2264 to 0.0767, *p* = 0.333). Male Proportion (%) was not significantly associated with prevalence (beta = 0.0015, 95% CI: −0.0180 to 0.0211, *p* = 0.877). More details were provided in Table 9 and Supplementary file 1.

For Moderate Depression: Publication Year was not significantly associated with prevalence (beta = 0.2547, 95% CI: −0.0851 to 0.5944, *p* = 0.142). Sample Size (log10) was significantly associated with prevalence (beta = −0.6163, 95% CI: −1.0355 to −0.1970, *p* = 0.004). Mean Age was not significantly associated with prevalence (beta = 0.0486, 95% CI: −0.0188 to 0.1160, *p* = 0.158). Male Proportion (%) was not significantly associated with prevalence (beta = −0.0182, 95% CI: −0.0379 to 0.0014, *p* = 0.069). More details were provided in Table 9 and Supplementary file 1.

For Severe Depression: Publication Year was not significantly associated with prevalence (beta = 0.5812, 95% CI: −0.1152 to 1.2776, *p* = 0.102). Sample Size (log10) was not significantly associated with prevalence (beta = −0.7291, 95% CI: −1.5833 to 0.1251, *p* = 0.094). Mean Age was not significantly associated with prevalence (beta = 0.1098, 95% CI: −0.0159 to 0.2355, *p* = 0.087). Male Proportion (%) was significantly associated with prevalence (beta = −0.0410, 95% CI: −0.0796 to −0.0024, *p* = 0.037). More details were provided in Table 9 and Supplementary file 1.

For Mild Anxiety: Publication Year was not significantly associated with prevalence (beta = 0.3382, 95% CI: −0.0297 to 0.7061, *p* = 0.072). Sample Size (log10) was not significantly associated with prevalence (beta = −0.1176, 95% CI: −0.6012 to 0.3661, *p* = 0.634). Mean Age was not significantly associated with prevalence (beta = −0.0032, 95% CI: −0.1742 to 0.1678, *p* = 0.971). Male Proportion (%) was not significantly associated with prevalence (beta = −0.0066, 95% CI: −0.0336 to 0.0205, *p* = 0.635). More details were provided in Table 9 and Supplementary file 1.

For Moderate Anxiety: Publication Year was significantly associated with prevalence (beta = 0.6485, 95% CI: 0.2157 to 1.0813, *p* = 0.003). Sample Size (log10) was not significantly associated with prevalence (beta = −0.5900, 95% CI: −1.2196 to 0.0396, *p* = 0.066). Mean Age was not significantly associated with prevalence (beta = 0.0558, 95% CI: −0.0314 to 0.1431, *p* = 0.210). Male Proportion (%) was not significantly associated with prevalence (beta = −0.0291, 95% CI: −0.0623 to 0.0041, *p* = 0.086). More details were provided in Table 9 and Supplementary file 1.

For Severe Anxiety: Publication Year was significantly associated with prevalence (beta = 0.7651, 95% CI: 0.0059 to 1.5243, *p* = 0.048). Sample Size (log10) was not significantly associated with prevalence (beta = −0.7248, 95% CI: −1.6832 to 0.2336, *p* = 0.138). Mean Age was not significantly associated with prevalence (beta = 0.0901, 95% CI: −0.0568 to 0.2370, *p* = 0.229). Male Proportion (%) was not significantly associated with prevalence (beta = −0.0315, 95% CI: −0.0848 to 0.0217, *p* = 0.246). More details were provided in Table 9 and Supplementary file 1.

For High Stress: Publication Year was not significantly associated with prevalence (beta = 0.0370, 95% CI: −0.8575 to 0.9314, *p* = 0.935). Sample Size (log10) was not significantly associated with prevalence (beta = −0.3804, 95% CI: −1.5541 to 0.7933, *p* = 0.525). Mean Age was not significantly associated with prevalence (beta = 0.0877, 95% CI: −0.1205 to 0.2958, *p* = 0.409). Male Proportion (%) was not significantly associated with prevalence (beta = −0.0140, 95% CI: −0.0667 to 0.0388, *p* = 0.603). More details were provided in Table 9 and Supplementary file 1.

### Leave-one-out sensitivity analysis

Leave-one-out sensitivity analyses were performed to evaluate the robustness of the pooled prevalence estimates. Each included study was sequentially excluded, and the meta-analyses were recalculated. Overall, the findings demonstrated high stability, with most pooled estimates varying by less than 5 percentage points. For overall depression, prevalence estimates ranged from 44.1% to 46.4% (range: 2.3%), indicating robust results. Similarly, overall anxiety estimates varied between 42.0% and 45.2% (range: 3.1%), while overall stress showed a wider range (42.1% to 48.0%; range: 5.9%), suggesting some sensitivity to individual studies, although the overall conclusions remained unchanged. Subgroup analyses by severity also demonstrated stability: prevalence ranges were 21.3%–23.4% for mild depression, 12.5%–13.7% for moderate depression, and 4.2%–5.4% for severe depression. For anxiety, prevalence estimates ranged from 22.2%–24.7% for mild anxiety, 13.3%–15.4% for moderate anxiety, and 6.6%–8.5% for severe anxiety. High perceived stress prevalence varied between 16.0% and 20.0% (range: 4.0%). Overall, these findings indicate that the pooled estimates were largely robust and not unduly influenced by any single study. Sensitivity Analysis Results were presented in Table 10 and Supplementary file 1.

**Table 10:** Sensitivity analysis results

**Outcome**	**Min**	**Max**	**Range**	**LOO Mean**	**LOO SD**	**Overall**	**Stable**
Overall Depression	44.1%	46.4%	2.3%	45.1%	0.48	45.1%	Yes
Overall Anxiety	42.0%	45.2%	3.1%	43.6%	0.61	43.6%	Yes
Overall Stress	42.1%	48.0%	5.9%	45.5%	1.40	45.6%	No
Mild Depression	21.3%	23.4%	2.1%	22.0%	0.43	22.0%	Yes
Moderate Depression	12.5%	13.7%	1.2%	12.9%	0.31	12.9%	Yes
Severe Depression	4.2%	5.4%	1.2%	4.7%	0.25	4.7%	Yes
Mild Anxiety	22.2%	24.7%	2.4%	23.1%	0.51	23.1%	Yes
Moderate Anxiety	13.3%	15.4%	2.1%	13.8%	0.48	13.8%	Yes
Severe Anxiety	6.6%	8.5%	2.0%	7.1%	0.44	7.1%	Yes
High Stress	16.0%	20.0%	4.0%	17.3%	1.27	17.3%	Yes

**Outcome**	**Min**	**Max**	**Range**	**LOO Mean**	**LOO SD**	**Overall**	**Stable**
Overall Depression	44.1%	46.4%	2.3%	45.1%	0.48	45.1%	Yes
Overall Anxiety	42.0%	45.2%	3.1%	43.6%	0.61	43.6%	Yes
Overall Stress	42.1%	48.0%	5.9%	45.5%	1.40	45.6%	No
Mild Depression	21.3%	23.4%	2.1%	22.0%	0.43	22.0%	Yes
Moderate Depression	12.5%	13.7%	1.2%	12.9%	0.31	12.9%	Yes
Severe Depression	4.2%	5.4%	1.2%	4.7%	0.25	4.7%	Yes
Mild Anxiety	22.2%	24.7%	2.4%	23.1%	0.51	23.1%	Yes
Moderate Anxiety	13.3%	15.4%	2.1%	13.8%	0.48	13.8%	Yes
Severe Anxiety	6.6%	8.5%	2.0%	7.1%	0.44	7.1%	Yes
High Stress	16.0%	20.0%	4.0%	17.3%	1.27	17.3%	Yes

Stable: Range < 5 percentage points

### Instrument-based sensitivity analysis

To assess whether pooled estimates were dependent on any single measurement instrument, all studies using each specific instrument were sequentially excluded and the meta-analysis recalculated.

For Overall Depression (overall: 45.1%): excluding PHQ-9 studies (*n* = 29): 39.0% (29.0%−50.0%); excluding DASS-21 studies (*n* = 12): 44.5% (36.5%−52.8%); excluding Other studies (*n* = 5): 46.5% (40.2%−52.9%); excluding BDI studies (*n* = 2): 44.7% (38.0%−51.6%); excluding CES-D studies (*n* = 3): 46.3% (39.6%−53.1%); excluding SDS studies (*n* = 2): 45.6% (38.8%−52.6%). More details were provided in Table 11 and Supplementary file 1.

**Table 11:** Instrument-based sensitivity analysis

Outcome	Excluded Tool	N Excluded	N Remaining	Prevalence	95% CI	Overall	Difference
Overall Depression	PHQ-9	29	25	39.0%	29.0–50.0%	45.1%	−6.1%
	DASS-21	12	42	44.5%	36.5–52.8%	45.1%	−0.6%
	Other	5	49	46.5%	40.2–52.9%	45.1%	1.4%
	BDI	2	52	44.7%	38.0–51.6%	45.1%	−0.4%
	CES-D	3	51	46.3%	39.6–53.1%	45.1%	1.2%
	SDS	2	52	45.6%	38.8–52.6%	45.1%	0.5%
Overall Anxiety	GAD-7	29	25	41.3%	27.7–56.3%	43.6%	−2.3%
	DASS-21	11	43	41.0%	31.3–51.5%	43.6%	−2.6%
	Other	7	47	40.5%	32.0–49.5%	43.6%	−3.1%
	SAS	4	50	48.7%	41.2–56.3%	43.6%	5.1%
	HADS	2	52	43.9%	35.2–52.9%	43.6%	0.3%
Overall Stress	PSS	7	19	35.7%	23.3–50.4%	45.6%	−9.8%
	DASS-21	11	15	51.8%	31.4–71.6%	45.6%	6.3%
	Other	7	19	49.2%	36.4–62.1%	45.6%	3.7%
Mild Depression	PHQ-9	18	14	19.6%	14.3–26.1%	22.0%	−2.4%
	Other	2	30	22.9%	18.5–28.0%	22.0%	0.9%
	DASS-21	8	24	24.1%	18.7–30.5%	22.0%	2.1%
	BDI	2	30	21.7%	17.4–26.8%	22.0%	−0.2%
	SDS	1	31	21.6%	17.4–26.5%	22.0%	−0.4%
	CES-D	1	31	21.3%	17.3–26.0%	22.0%	−0.7%
Moderate Depression	PHQ-9	18	13	12.4%	8.7–17.3%	12.9%	−0.5%
	Other	3	28	13.8%	10.8–17.4%	12.9%	0.9%
	DASS-21	7	24	12.0%	8.7–16.3%	12.9%	−0.9%
	BDI	2	29	12.7%	9.7–16.4%	12.9%	−0.2%
	SDS	1	30	13.2%	10.2–16.8%	12.9%	0.3%
Severe Depression	PHQ-9	18	14	4.7%	1.4–14.4%	4.7%	0.0%
	Other	3	29	4.5%	2.6–7.8%	4.7%	−0.1%
	DASS-21	8	24	4.5%	2.4–8.1%	4.7%	−0.2%
	SDS	1	31	5.4%	3.2–8.9%	4.7%	0.7%
	CES-D	1	31	4.6%	2.5–8.1%	4.7%	−0.1%
	BDI	1	31	4.5%	2.5–7.9%	4.7%	−0.2%
Mild Anxiety	GAD-7	19	14	15.2%	9.6–23.3%	23.1%	−7.9%
	Other	3	30	22.0%	16.9–28.1%	23.1%	−1.2%
	DASS-21	8	25	27.0%	20.6–34.6%	23.1%	3.9%
	SAS	2	31	25.7%	21.2–30.9%	23.1%	2.6%
	HADS	1	32	23.0%	17.8–29.1%	23.1%	−0.2%
Moderate Anxiety	GAD-7	19	13	12.1%	5.4–24.9%	13.8%	−1.7%
	Other	3	29	12.9%	8.7–18.7%	13.8%	−0.9%
	DASS-21	7	25	12.8%	8.0–19.7%	13.8%	−1.0%
	SAS	2	30	16.9%	13.5–20.9%	13.8%	3.1%
	HADS	1	31	13.4%	9.3–19.1%	13.8%	−0.4%
Severe Anxiety	GAD-7	19	14	8.0%	2.5–22.5%	7.1%	0.9%
	Other	3	30	6.6%	3.4–12.6%	7.1%	−0.5%
	DASS-21	8	25	5.5%	2.7–10.7%	7.1%	−1.6%
	SAS	2	31	9.5%	5.9–15.0%	7.1%	2.4%
	HADS	1	32	6.8%	3.7–12.4%	7.1%	−0.3%
High Stress	PSS	7	12	11.6%	4.4–27.3%	17.3%	−5.8%
	Other	4	15	18.2%	10.1–30.5%	17.3%	0.9%
	DASS-21	8	11	22.2%	12.5–36.5%	17.3%	4.9%

For Overall Anxiety (overall: 43.6%): excluding GAD-7 studies (*n* = 29): 41.3% (27.7%−56.3%); excluding DASS-21 studies (*n* = 11): 41.0% (31.3%−51.5%); excluding Other studies (*n* = 7): 40.5% (32.0%−49.5%); excluding SAS studies (*n* = 4): 48.7% (41.2%−56.3%); excluding HADS studies (*n* = 2): 43.9% (35.2%−52.9%). More details were provided in Table 11 and Supplementary file 1.

For Overall Stress (overall: 45.6%): excluding PSS studies (*n* = 7): 35.7% (23.3%−50.4%); excluding DASS-21 studies (*n* = 11): 51.8% (31.4%−71.6%); excluding Other studies (*n* = 7): 49.2% (36.4%−62.1%). More details were provided in Table 11 and Supplementary file 1.

For Mild Depression (overall: 22.0%): excluding PHQ-9 studies (*n* = 18): 19.6% (14.3%−26.1%); excluding Other studies (*n* = 2): 22.9% (18.5%−28.0%); excluding DASS-21 studies (*n* = 8): 24.1% (18.7%−30.5%); excluding BDI studies (*n* = 2): 21.7% (17.4%−26.8%); excluding SDS studies (*n* = 1): 21.6% (17.4%−26.5%); excluding CES-D studies (*n* = 1): 21.3% (17.3%−26.0%). More details were provided in Table 11 and Supplementary file 1.

For Moderate Depression (overall: 12.9%): excluding PHQ-9 studies (*n* = 18): 12.4% (8.7%−17.3%); excluding Other studies (*n* = 3): 13.8% (10.8%−17.4%); excluding DASS-21 studies (*n* = 7): 12.0% (8.7%−16.3%); excluding BDI studies (*n* = 2): 12.7% (9.7%−16.4%); excluding SDS studies (*n* = 1): 13.2% (10.2%−16.8%). More details were provided in Table 11 and Supplementary file 1.

For Severe Depression (overall: 4.7%): excluding PHQ-9 studies (*n* = 18): 4.7% (1.4%−14.4%); excluding Other studies (*n* = 3): 4.5% (2.6%−7.8%); excluding DASS-21 studies (*n* = 8): 4.5% (2.4%−8.1%); excluding SDS studies (*n* = 1): 5.4% (3.2%−8.9%); excluding CES-D studies (*n* = 1): 4.6% (2.5%−8.1%); excluding BDI studies (*n* = 1): 4.5% (2.5%−7.9%).

For Mild Anxiety (overall: 23.1%): excluding GAD-7 studies (*n* = 19): 15.2% (9.6%−23.3%); excluding Other studies (*n* = 3): 22.0% (16.9%−28.1%); excluding DASS-21 studies (*n* = 8): 27.0% (20.6%−34.6%); excluding SAS studies (*n* = 2): 25.7% (21.2%−30.9%); excluding HADS studies (*n* = 1): 23.0% (17.8%−29.1%). More details were provided in Table 11 and Supplementary file 1.

For Moderate Anxiety (overall: 13.8%): excluding GAD-7 studies (*n* = 19): 12.1% (5.4%−24.9%); excluding Other studies (*n* = 3): 12.9% (8.7%−18.7%); excluding DASS-21 studies (*n* = 7): 12.8% (8.0%−19.7%); excluding SAS studies (*n* = 2): 16.9% (13.5%−20.9%); excluding HADS studies (*n* = 1): 13.4% (9.3%−19.1%). More details were provided in Table 11 and Supplementary file 1.

For Severe Anxiety (overall: 7.1%): excluding GAD-7 studies (*n* = 19): 8.0% (2.5%−22.5%); excluding Other studies (*n* = 3): 6.6% (3.4%−12.6%); excluding DASS-21 studies (*n* = 8): 5.5% (2.7%−10.7%); excluding SAS studies (*n* = 2): 9.5% (5.9%−15.0%); excluding HADS studies (*n* = 1): 6.8% (3.7%−12.4%). More details were provided in Table 11 and Supplementary file 1.

For High Stress (overall: 17.3%): excluding PSS studies (*n* = 7): 11.6% (4.4%−27.3%); excluding other studies (*n* = 4): 18.2% (10.1%−30.5%); excluding DASS-21 studies (*n* = 8): 22.2% (12.5%−36.5%). More details were provided in Table 11 and Supplementary file 1.

### Publication bias

Publication bias was assessed using funnel plots in addition to Egger and Peters tests. Across the ten outcomes examined, Egger's test suggested significant funnel plot asymmetry in three subgroups (mild depression, mild anxiety, and severe anxiety), whereas Peters' test indicated potential small-study effects in seven of the ten outcomes (overall depression, overall stress, mild depression, mild anxiety, moderate depression, moderate anxiety, and high stress). As noted in the Methods section, these approaches have recognized limitations in proportion meta-analyses characterized by substantial between-study heterogeneity. Therefore, statistically significant findings should be interpreted cautiously as indicative of potential small-study effects rather than definitive evidence of publication bias.

For overall depression, Egger test did not demonstrate significant funnel plot asymmetry (z = 1.15, *p* = 0.255), whereas Peters test was statistically significant (z = −2.50, *p* = 0.016). Similarly, for overall anxiety, no significant asymmetry was detected using Egger test (z = 0.77, *p* = 0.447), and Peters test was also non-significant (z = −1.63, *p* = 0.108). In the analysis of overall stress, Egger test did not reveal significant asymmetry (z = 1.46, *p* = 0.157), although Peters test suggested potential small-study effects (z = −4.06, *p* < 0.001). More details were presented in Table 12 and Supplementary file 1.

**Table 12:** Publication bias assessment

Outcome	Egger z	Egger p	Peters z	Peters p	Asymmetry
Overall Depression	1.15	0.255	−2.50	0.016	No
Overall Anxiety	0.77	0.447	−1.63	0.108	No
Overall Stress	1.46	0.157	−4.06	< 0.001	No
Mild Depression	−2.50	0.018	2.75	0.010	Yes
Moderate Depression	0.23	0.822	−5.20	< 0.001	No
Severe Depression	−1.80	0.081	−1.59	0.123	No
Mild Anxiety	−2.64	0.013	3.92	< 0.001	Yes
Moderate Anxiety	−0.79	0.438	−4.67	< 0.001	No
Severe Anxiety	−2.62	0.013	0.07	0.943	Yes
High Stress	−0.31	0.763	−4.74	< 0.001	No

Severity-stratified analyses for depression showed that mild depression demonstrated significant funnel plot asymmetry according to Egger test (z = −2.50, *p* = 0.018), with Peters test also reaching statistical significance (z = 2.75, *p* = 0.010). Given the very high heterogeneity (I2 > 99%), these findings may reflect genuine differences between smaller and larger studies rather than true publication bias. Moderate depression did not show significant asymmetry on Egger test (z = 0.23, *p* = 0.822), although Peters test was significant (z = −5.20, *p* < 0.001). Severe depression similarly showed no significant asymmetry according to Egger test (z = −1.80, *p* = 0.081), while Peters test remained non-significant (z = −1.59, *p* = 0.123). More details were presented in Table 12 and Supplementary file 1.

For anxiety severity subgroups, mild anxiety demonstrated significant funnel plot asymmetry on Egger test (z = −2.64, *p* = 0.013), with Peters test also indicating potential small-study effects (z = 3.92, *p* < 0.001). As with depression outcomes, the substantial heterogeneity observed across studies (I2 > 99%) suggests that these findings should be interpreted cautiously. Moderate anxiety did not demonstrate significant asymmetry on Egger test (z = −0.79, *p* = 0.438), although Peters test was significant (z = −4.67, *p* < 0.001). Severe anxiety showed significant asymmetry on Egger test (z = −2.62, *p* = 0.013), whereas Peters test was non-significant (z = 0.07, *p* = 0.943), again suggesting that observed asymmetry may be attributable to heterogeneity and study-scale differences rather than publication bias alone. More details were presented in Table 12 and Supplementary file 1.

For high stress, Egger test did not reveal evidence of significant asymmetry (z = −0.31, *p* = 0.763), while Peters test suggested potential small-study effects (z = −4.74, *p* < 0.001). Overall, the mixed findings between Egger and Peters tests, together with the extremely high heterogeneity across analyses, indicate that the observed asymmetry should be interpreted with caution. More details were presented in Table 12 and Supplementary file 1.

## Discussion

This systematic review and meta-analysis provides a comprehensive synthesis of post-pandemic mental health outcomes among university students, encompassing data from 63 cross-sectional studies and over 255,000 participants worldwide. The findings indicate a substantial burden of depression, anxiety, and stress in this population, highlighting the enduring psychological impact of the COVID-19 pandemic on higher education students.

The pooled prevalence of overall depression (45.1%) and anxiety (43.6%) among university students is concerning, indicating that approximately one in three students experienced clinically relevant symptoms during the post-pandemic period. These findings are comparable to those reported in previous systematic reviews conducted during the pandemic, which identified pooled prevalence estimates of approximately 33.6% for depression and 39.0% for anxiety among university students [8]. The pooled anxiety prevalence estimated in the present study (43.6%) appears comparable to or somewhat higher than that reported during the acute pandemic phase (39.0%), suggesting that psychological distress among university students has not returned to pre-pandemic levels and may have been sustained or exacerbated in the post-pandemic period. Stress prevalence, although lower at 26.8%, remained clinically significant. This finding is consistent with previous post-pandemic studies reporting that while perceived stress levels may partially normalize over time, symptoms of anxiety and depression often persist longer [13].

Severity-specific analyses revealed that mild symptoms were most common, whereas moderate and severe cases were less frequent. Similar patterns have been reported in previous studies showing that mild-to-moderate psychological symptoms predominate among university students [99, 100]. However, despite their lower prevalence, moderate and severe symptoms may still substantially impair academic performance, social functioning, and overall well-being. The predominance of mild symptoms may reflect increased mental health awareness and improved psychological screening during the post-pandemic period, potentially allowing earlier recognition of psychological distress before progression to more severe clinical manifestations.

Subgroup analyses highlighted significant regional variations in mental health outcomes. Overall depression prevalence was highest in Africa (70.1%) and the Middle East (63.3%), whereas Asia and North America reported relatively lower rates. Similar regional patterns were observed for anxiety symptoms. These findings align with previous studies suggesting that socioeconomic instability, limited access to mental health resources, educational disruption, and political uncertainty may disproportionately affect students in low- and middle-income settings [101–103]. In contrast, lower prevalence estimates observed in some higher-income regions may reflect a range of factors, that should be analysed in future investigations, including greater availability of institutional support systems, cultural differences in the recognition or reporting of psychological distress, or methodological variation across studies.

Interestingly, the test for subgroup differences in severe depression was not statistically significant, suggesting that severe depressive symptoms may be relatively consistently distributed across regions. In contrast, severe anxiety demonstrated significant regional variation, implying that extreme anxiety symptoms may be more sensitive to contextual stressors, uncertainty, and variability in institutional or social support systems. Differences in cultural perceptions of mental health and symptom reporting may also contribute to this regional heterogeneity.

Consistent with previous studies, low socioeconomic status (SES) emerged as an important risk factor for depression and anxiety. Several included studies reported markedly higher prevalence estimates among students from lower-income backgrounds, with depression prevalence exceeding 70% in some populations [101]. Similarly, previous studies reported prevalence rates of 44% for depression and 66% for anxiety among socioeconomically vulnerable students [38], substantially higher than estimates reported in other populations [102, 103]. These findings support earlier evidence demonstrating that financial stress, economic instability, and limited access to support resources are strongly associated with adverse mental health outcomes among university students. Additionally, students with pre-existing mental health conditions prior to university enrolment appeared particularly vulnerable, emphasizing the importance of early psychological screening and targeted intervention programs for high-risk groups.

Regarding parental education, our findings are consistent with Arias-Coronel et al. (2025), who found no evidence supporting a protective effect of higher parental educational attainment on depression or anxiety prevalence [38]. This finding contrasts with previous assumptions that parental education may buffer psychological distress through improved academic support or family resources. Instead, our findings suggest that direct economic stability and psychosocial support may exert greater influence on student mental health outcomes than parental educational background alone [104].

Academic stress was consistently associated with elevated levels of anxiety and depression, which agrees with previous studies linking academic pressure and psychological distress among university students [105]. Most prior research has reported higher anxiety prevalence relative to depression in academically demanding environments [99, 100], which is comparable to our findings. However, some studies have identified higher depression prevalence associated with chronic academic stress and burnout [106]. These discrepancies may be related to differences in academic disciplines, educational systems, timing of assessments, and mental health assessment tools across studies. Furthermore, because many included studies focused on students in health-related disciplines, the generalizability of these findings to broader university populations should be interpreted cautiously.

Female students demonstrated greater susceptibility to mental health challenges, with higher rates of depression and anxiety compared with male students, which is consistent with previous literature conducted during and after the pandemic [107]. Potential explanations include gender differences in coping strategies, emotional processing, help-seeking behaviors, and exposure to psychosocial stressors. However, the overrepresentation of female participants in several included studies may have partially influenced these estimates.

Some studies additionally highlighted the interaction between gender, academic stress, and low social support in exacerbating psychological symptoms [108]. Sexual minority students appeared particularly vulnerable, with reported anxiety prevalence ranging from 96 to 100% and depression prevalence ranging from 70 to 100% in some studies [38]. These estimates are substantially higher than those reported among heterosexual students in previous literature [108], potentially reflecting the cumulative effects of minority stress, discrimination, social stigma, and reduced access to supportive environments. Interestingly, some studies suggested that being in a romantic relationship during university may serve as a protective factor among sexual minority students by strengthening emotional and social support networks.

Meta-regression analyses indicated that publication year was not consistently associated with prevalence for depression or stress but was positively associated with moderate and severe anxiety, suggesting a possible gradual increase in anxiety symptoms throughout the post-pandemic period. This finding contrasts with earlier expectations that mental health symptoms would progressively improve following the return to conventional academic life. One possible explanation is that students may continue to experience residual psychological effects of the pandemic alongside additional societal stressors occurring during the post-pandemic period, including economic instability and geopolitical conflicts. Larger sample sizes were inversely associated with moderate depression prevalence, which may reflect overestimation of prevalence in smaller studies due to selective sampling or reporting bias.

### Strengths and limitations points

This systematic review and meta-analysis has several strengths. To our knowledge, it is among the few studies specifically focusing on the post-pandemic period to evaluate the prevalence and severity of depression, anxiety, and stress among university students. By distinguishing the post–COVID-19 phase from the acute pandemic period, this review provides updated evidence regarding the persistence of psychological distress following the relaxation of pandemic-related restrictions. Another major strength is the large cumulative sample size, which exceeded 255,000 university students across diverse geographic and academic settings, thereby providing a broad overview of post-pandemic mental health patterns. In addition, the inclusion of severity-stratified analyses allowed for a more detailed assessment of symptom burden beyond overall prevalence estimates. The review also followed rigorous methodological standards, including comprehensive database searching, duplicate screening and data extraction, quality assessment, and adherence to PRISMA guidelines.

Nevertheless, several limitations should be acknowledged when interpreting the findings. First, substantial statistical heterogeneity was observed across nearly all analyses (I2 frequently > 99%), indicating considerable variability between studies. This heterogeneity likely reflects differences in study populations, cultural contexts, sampling strategies, timing of assessment, and mental health measurement tools. Second, the included studies used a wide range of psychometric instruments and cut-off thresholds for depression, anxiety, and stress assessment, which may limit the comparability of pooled prevalence estimates. Furthermore, the construct of stress showed greater variability across studies than depression or anxiety, with a small number of studies administering trauma-related instruments (IES, PCL) alongside general stress measures. Although these data were not pooled, this construct heterogeneity may have contributed to the wider confidence interval, and the relatively lower stability of the stress prevalence estimate in the leave-one-out analysis. Severity classifications were not fully standardized across instruments, and some stress-related measures assessed distinct constructs, including perceived stress and trauma-related symptoms, which may have influenced the interpretation of pooled stress outcomes.

Third, the included studies were cross-sectional in design, limiting causal inference and preventing assessment of temporal changes in mental health outcomes at the individual level. Fourth, despite the use of publication bias assessments, including Egger and Peters tests, these methods have recognized limitations in proportion meta-analyses with high heterogeneity and may reflect small-study effects or methodological variability rather than true publication bias. Therefore, findings related to funnel plot asymmetry should be interpreted cautiously.

Additionally, mental health outcomes observed during the post-pandemic period may reflect the cumulative effects of multiple contemporaneous societal and global stressors rather than the isolated impact of COVID-19 alone.

Finally, although the large pooled sample enhances the breadth of the evidence, the methodological variability and uncertainty surrounding pooling strategies mean that the reported prevalence estimates should be interpreted as broad indicators of psychological distress rather than precise epidemiological estimates. Consequently, the findings are more suitable for highlighting the potential persistence of student mental health concerns in the post-pandemic period than for supporting definitive policy or institutional recommendations. Further longitudinal studies using standardized and comparable assessment frameworks are needed to strengthen the reliability and interpretability of future evidence.

### Clinical implications and future research

The findings of this systematic review and meta-analysis reveal that depression, anxiety, and stress remain highly prevalent among university students in the post-pandemic period, highlighting a sustained public health concern within higher education settings. Beyond the descriptive prevalence estimates, these findings may have implications for clinical practice, institutional planning, and future research. However, the findings presented here reflect descriptive prevalence data rather than evidence-based recommendations for specific policy frameworks or intervention models. Accordingly, any suggestions derived from these findings should be considered exploratory and subject to adaptation based on local context, available resources, and additional sources of evidence.

The consistently high prevalence of mental health symptoms across diverse geographic regions underscores the importance of awareness among healthcare providers working with university populations. Routine mental health screening within university health services may facilitate early identification of at-risk students, though the optimal screening strategies and referral pathways would require further investigation. The observed regional variations in prevalence estimates suggest that clinical approaches should be informed by locally relevant data rather than relying exclusively on pooled global estimates.

In addition, the magnitude of the mental health burden identified in this review may warrant consideration within institutional strategic planning processes, specially in universities that have not already done so. While the findings of this review cannot directly support specific policy recommendations, they may contribute to the broader evidence base informing discussions about mental health in higher education.

Finally, this review highlights several gaps in the current evidence base that warrant further investigation. Future research should examine the effectiveness of specific mental health interventions within university settings, using rigorous study designs that can support causal inference. Studies employing longitudinal designs would be particularly valuable for understanding the trajectory of mental health symptoms beyond the pandemic period. Additionally, research examining the implementation and scaling of mental health services within diverse higher education contexts would complement the descriptive prevalence data presented here.

## Conclusions

This systematic review and meta-analysis suggests that symptoms of depression, anxiety, and stress remain common among university students during the post-pandemic period. The findings indicate a substantial burden of psychological distress across diverse geographic and academic settings, with anxiety emerging as the most frequently reported outcome. Severity-stratified analyses further demonstrated that mild symptoms were more prevalent than moderate or severe forms across most outcomes.

However, these findings should be interpreted cautiously due to the considerable methodological and clinical heterogeneity across included studies. Differences in the psychological constructs measured across stress-related instruments may have influenced the interpretation of stress prevalence findings.

The current review suggests that psychological distress among university students continues to represent an important public health and educational concern beyond the acute phase of the COVID-19 pandemic. The findings highlight the need for further longitudinal and methodologically standardized research using more consistent assessment frameworks to better characterize post-pandemic mental health trends and improve the reliability of future prevalence estimates. Such efforts may help inform future student mental health strategies, support services, and evidence-based interventions across university settings.

## Supplementary Information

Supplementary Material 1

Supplementary Material 2

Supplementary Material 3

## Data Availability

All data analysed during this study were obtained from previously published studies cited in the manuscript.

## Abbreviations

BAI: Beck Anxiety Inventory
BDI: Beck Depression Inventory
BDI-II: Beck Depression Inventory - Second Edition
CES-D: Center for Epidemiological Studies Depression Scale
CI: Confidence Interval
COVID-19: Coronavirus Disease 2019
DASS-21: Depression, Anxiety and Stress Scale - 21 Items
DSM-5: Diagnostic and Statistical Manual of Mental Disorders – Fifth Edition
GAD: Generalized Anxiety Disorder
GAD-2: Generalized Anxiety Disorder – 2 Items
GAD-7: Generalized Anxiety Disorder – 7 Items
HAM-A: Hamilton Anxiety Rating Scale
HADS-A: Hospital Anxiety and Depression Scale – Anxiety Subscale
IES-6: Impact of Event Scale – 6 Items
IES-R: Impact of Event Scale – Revised
I2: I-squared (heterogeneity statistic)
JBI: Joanna Briggs Institute
OSF: Open Science Framework
PHQ: Patient Health Questionnaire
PHQ-9: Patient Health Questionnaire – 9 Items
PCL-C: PTSD Checklist – Civilian Version
PCL-5: PTSD Checklist for DSM-5
PRISMA: Preferred Reporting Items for Systematic Reviews and Meta-Analyses
PSS-10: Perceived Stress Scale – 10 Items
PSS-14: Perceived Stress Scale – 14 Items
PTSD: Post-traumatic Stress Disorder
RCT: Randomized Controlled Trial
REML: Restricted Maximum Likelihood
SAS: Self-Rating Anxiety Scale
SDS: Self-Rating Depression Scale
SDG: Sustainable Development Goals
SE: Standard Error
SES: Socioeconomic Status
τ2: Tau-squared (between-study variance)
U.S.: United States
WHO: World Health Organization
WoS: Web of Science
