# Supplementary material for: Prevalence of depression, anxiety, and stress among university students in the post-COVID-19 period: a systematic review and meta-analysis
Source: BMC Psychol. 2026 Jun 29;14:1332. doi: 10.1186/s40359-026-05066-4 (PMC13573407; doi:10.1186/s40359-026-05066-4)
Supplement: Supplementary file 1 [file 40359_2026_5066_MOESM1_ESM.pdf]

Figure 2A: Studies by Region

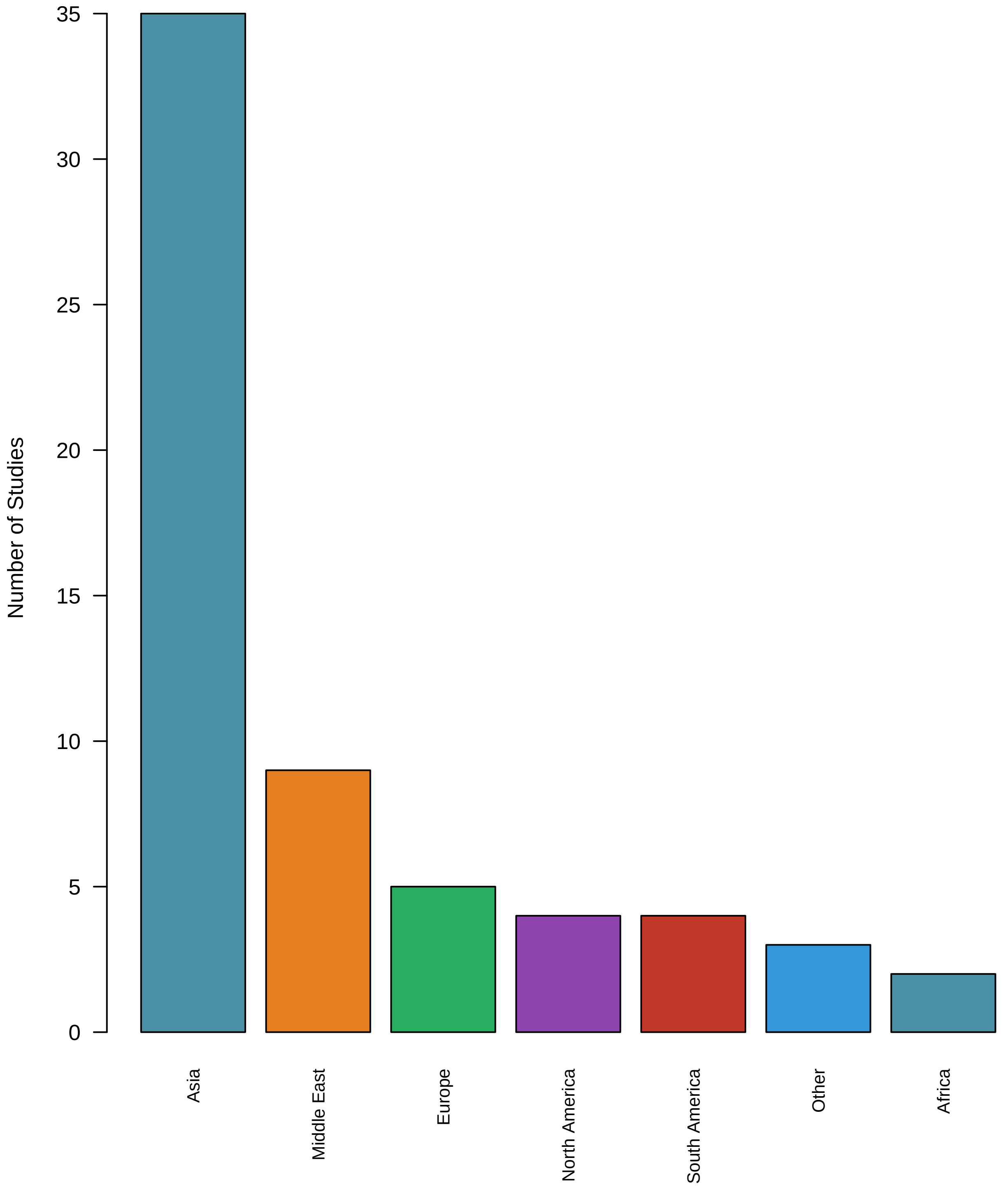

Figure 2B: Studies by Publication Year

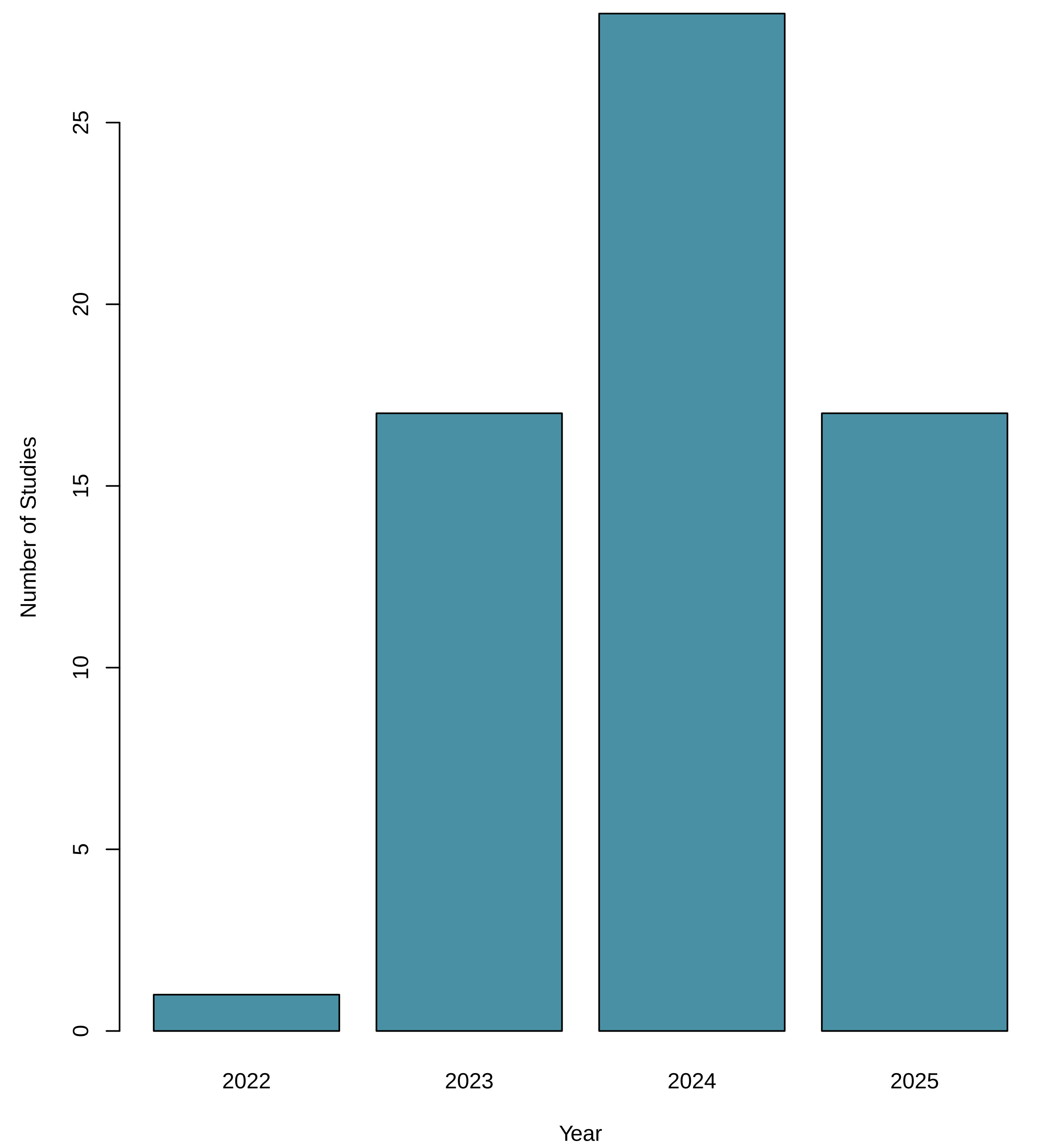

Figure 2C: Sample Size Distribution

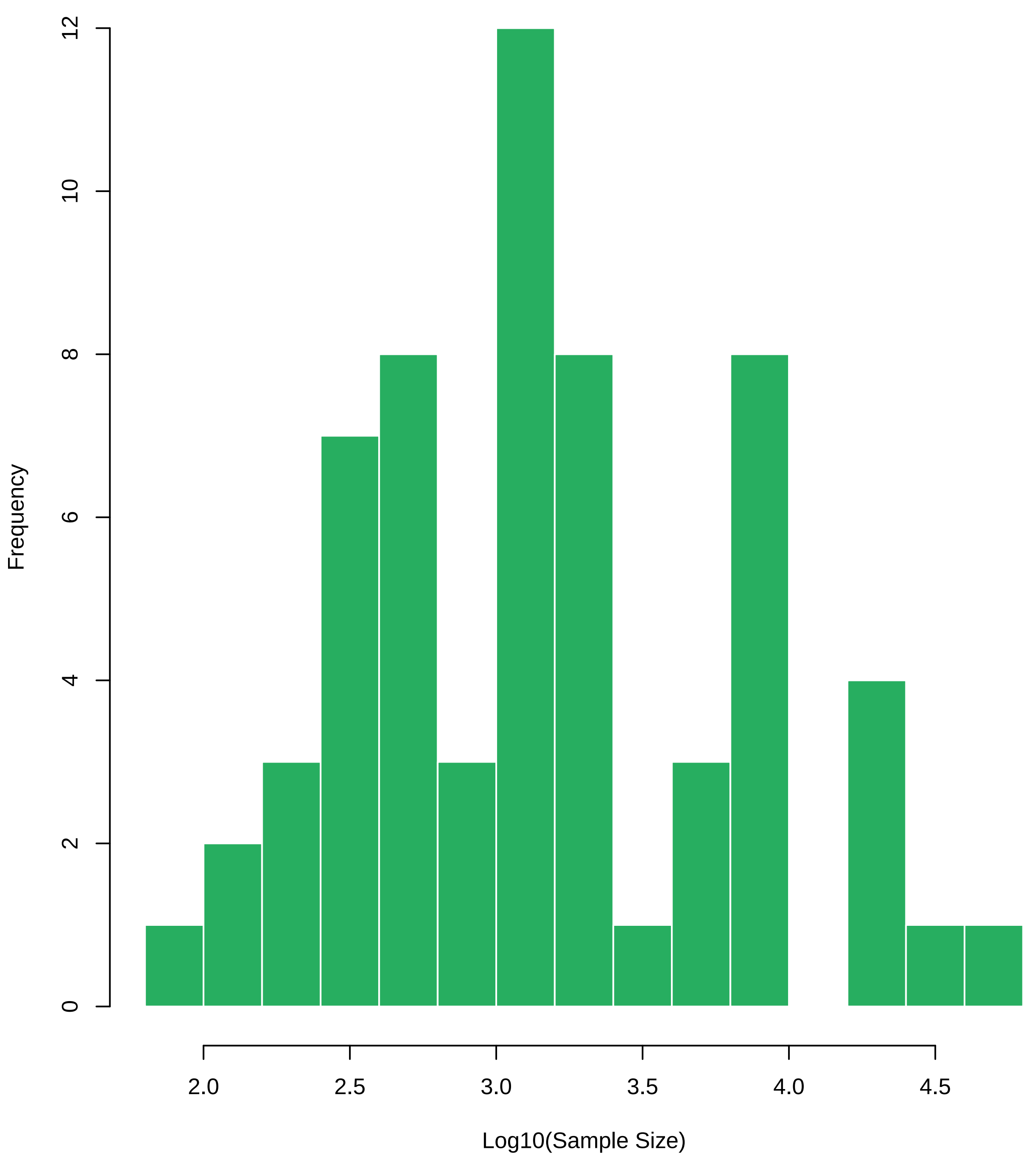

Figure 2D: Depression Screening Tools

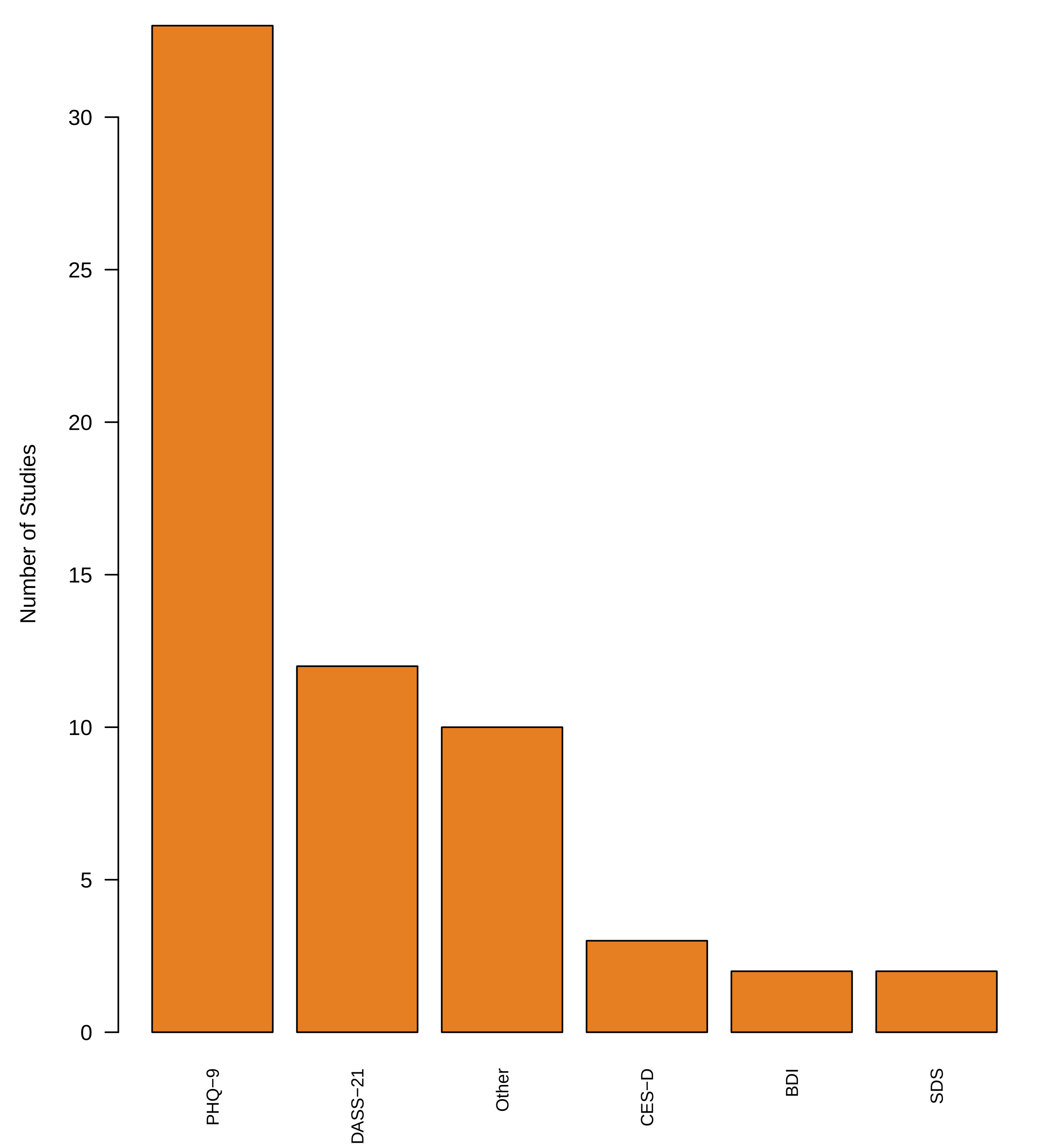

Figure 3A: Forest Plot − Depression Prevalence

N = 54 studies

Study	Events	N	Prevalence (%)	95% CI	Weight
Li et al. (2022)	2984	6905	0.43	[0.42; 0.44]	−−%
Alageel et al. (2023)	340	434	0.78	[0.74; 0.82]	−−%
Cao & Zheng (2023)	853	4705	0.18	[0.17; 0.19]	−−%
Chen et al. (2023)	5837	22624	0.26	[0.25; 0.26]	−−%
Deng & Zhang (2023)	358	1083	0.33	[0.30; 0.36]	−−%
He et al. (2023)	190	378	0.50	[0.45; 0.55]	−−%
Huang & Liu (2023)	401	1176	0.34	[0.31; 0.37]	−−%
Kavvadas et al. (2023)	833	1497	0.56	[0.53; 0.58]	−−%
Liu et al. (2023)	1275	2381	0.54	[0.52; 0.56]	−−%
Lv et al. (2023)	207	915	0.23	[0.20; 0.25]	−−%
Martínez−Líbano et al. (2023)	670	1062	0.63	[0.60; 0.66]	−−%
Nahrin et al. (2023)	819	1523	0.54	[0.51; 0.56]	−−%
Pérez et al. (2023)	4771	6798	0.70	[0.69; 0.71]	−−%
Qiao et al. (2023)	241	384	0.63	[0.58; 0.68]	−−%
Rajasekhar et al. (2023)	708	2035	0.35	[0.33; 0.37]	−−%
Ramón−Arbués et al. (2023)	13	339	0.04	[0.02; 0.06]	−−%
Xia et al. (2023)	557	1016	0.55	[0.52; 0.58]	−−%
Alotaibi et al. (2024)	681	1142	0.60	[0.57; 0.62]	−−%
Chen et al. (A) (2024)	1386	2046	0.68	[0.66; 0.70]	−−%
Chen et al. (B) (2024)	715	4858	0.15	[0.14; 0.16]	−−%
Feng et al. (2024)	905	2764	0.33	[0.31; 0.35]	−−%
Gao et al. (A) (2024)	430	3049	0.14	[0.13; 0.15]	−−%
Jiang et al. (2024)	176	757	0.23	[0.20; 0.26]	−−%
Kharroubi et al. (2024)	209	261	0.80	[0.75; 0.85]	−−%
Kilic et al. (2024)	254	518	0.49	[0.45; 0.53]	−−%
Lai et al. (2024)	137	222	0.62	[0.55; 0.68]	−−%
Lee et al. (2024)	540	1359	0.40	[0.37; 0.42]	−−%
Li et al. (A) (2024)	18163	30475	0.60	[0.59; 0.60]	−−%
Makki et al. (2024)	374	520	0.72	[0.68; 0.76]	−−%
Martínez−Esquivel et al. (2024)	59	76	0.78	[0.67; 0.86]	−−%
Milic et al. (2024)	884	2137	0.41	[0.39; 0.43]	−−%
Murthy et al. (2024)	75	223	0.34	[0.27; 0.40]	−−%
Saqib & Gazerani (2024)	102	132	0.77	[0.69; 0.84]	−−%
Su et al. (2024)	515	1327	0.39	[0.36; 0.41]	−−%
Sun et al. (2024)	144	498	0.29	[0.25; 0.33]	−−%
Wang et al. (2024)	3799	7617	0.50	[0.49; 0.51]	−−%
Wu et al. (2024)	472	1255	0.38	[0.35; 0.40]	−−%
Xin et al. (2024)	2452	4254	0.58	[0.56; 0.59]	−−%
Xu et al. (2024)	2284	18578	0.12	[0.12; 0.13]	−−%
Yaghmour et al. (2024)	126	382	0.33	[0.28; 0.38]	−−%
Ahmed et al. (2025)	1650	1891	0.87	[0.86; 0.89]	−−%
Alawadhi et al. (2025)	479	744	0.64	[0.61; 0.68]	−−%
Arias−Coronel et al. (2025)	177	394	0.45	[0.40; 0.50]	−−%
Barr−Porter et al. (2025)	194	536	0.36	[0.32; 0.40]	−−%
de Alencar Ribeiro et al. (2025)	72	179	0.40	[0.33; 0.48]	−−%
Dubovi & Edwards (2025)	265	1019	0.26	[0.23; 0.29]	−−%
Erbil et al. (2025)	244	500	0.49	[0.44; 0.53]	−−%
Flesaker et al. (2025)	6305	19265	0.33	[0.32; 0.33]	−−%
Frausing et al. (2025)	135	152	0.89	[0.83; 0.93]	−−%
Han et al. (B) (2025)	4060	41620	0.10	[0.09; 0.10]	−−%
Kong et al. (2025)	2181	6378	0.34	[0.33; 0.35]	−−%
Kunda et al. (2025)	265	595	0.45	[0.40; 0.49]	−−%
Pratummanee et al. (2025)	603	1611	0.37	[0.35; 0.40]	−−%
Sudershan et al. (2025)	1135	1471	0.77	[0.75; 0.79]	−−%
**Common effect model**		**216060**	**0.34**	**[0.34; 0.34]**	**.**
**Random effects model**			**0.45**	**[0.38; 0.52]**	**100.0%**

Heterogeneity: $I^2 = 99.8\%$, $\tau^2 = 1.0262$, $p = 0$

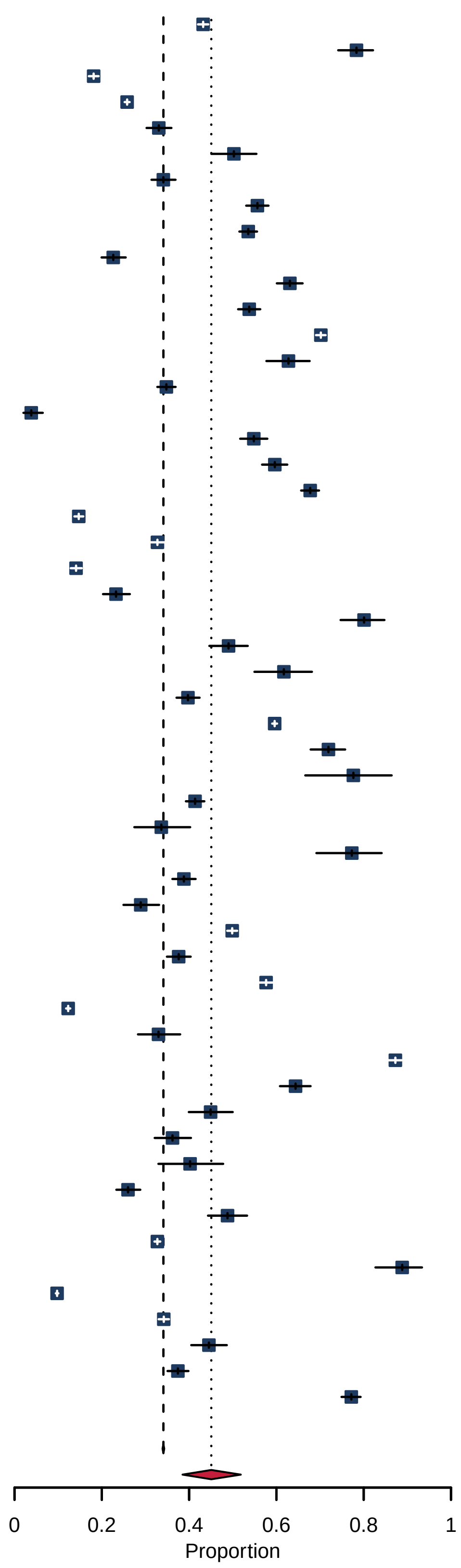

Figure 3B: Leave−One−Out Analysis − Depression Prevalence

Pooled estimate after omitting each study

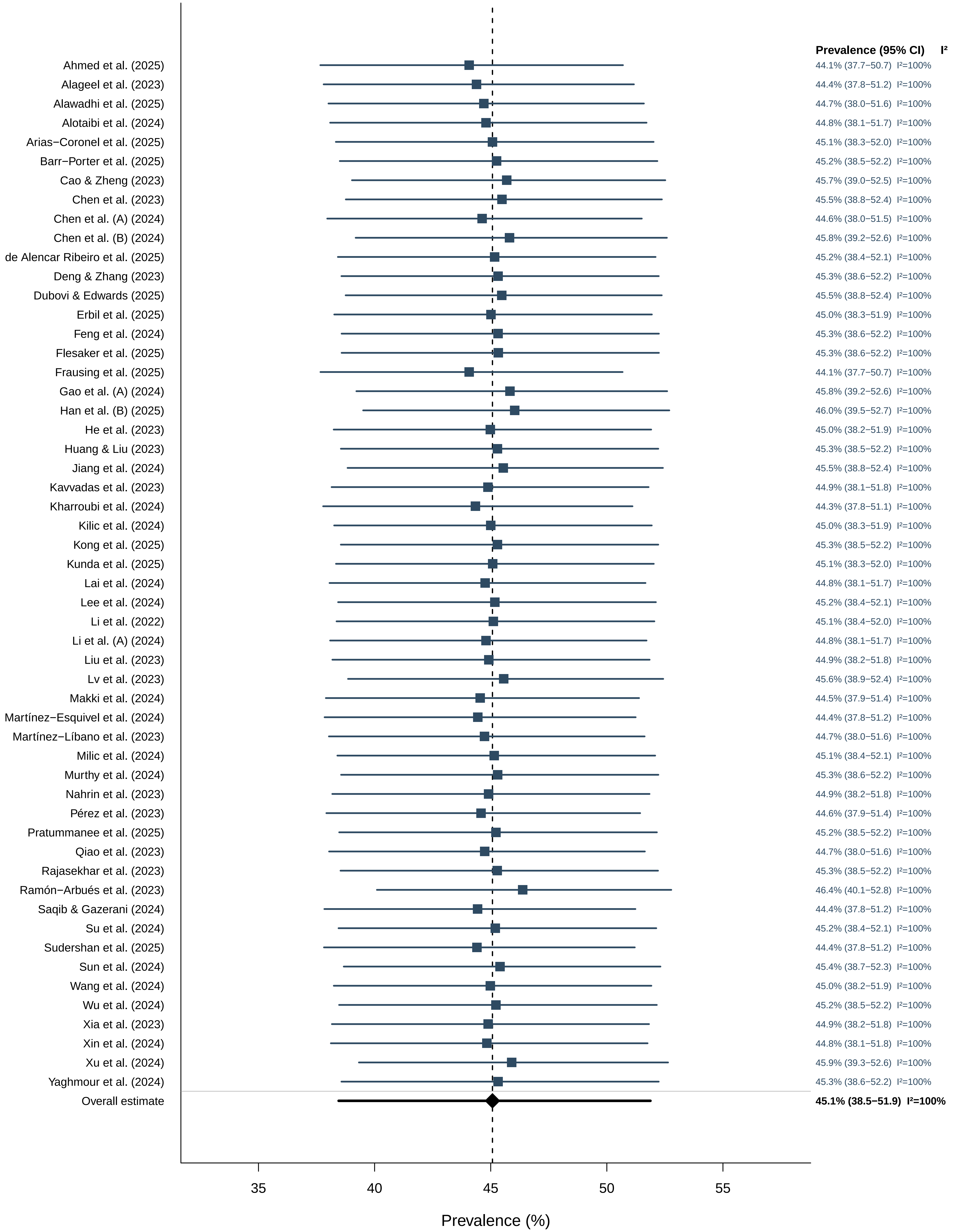

Figure 3C: Subgroup Analysis − Depression Prevalence

Stratified by study characteristics

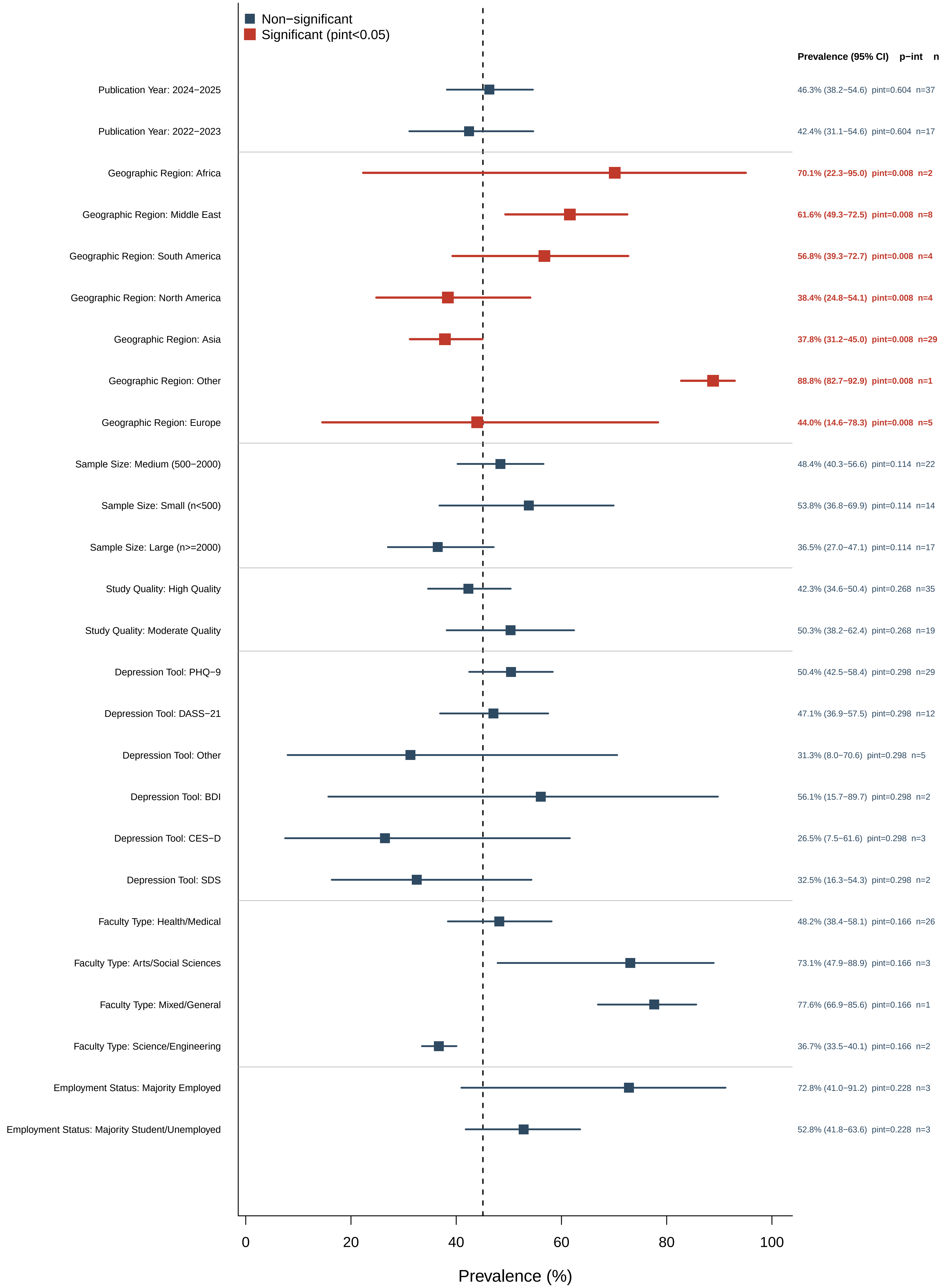

Figure 3D: Meta−Regression Analysis

Depression Prevalence

Effect of baseline covariates on prevalence (univariate analysis)

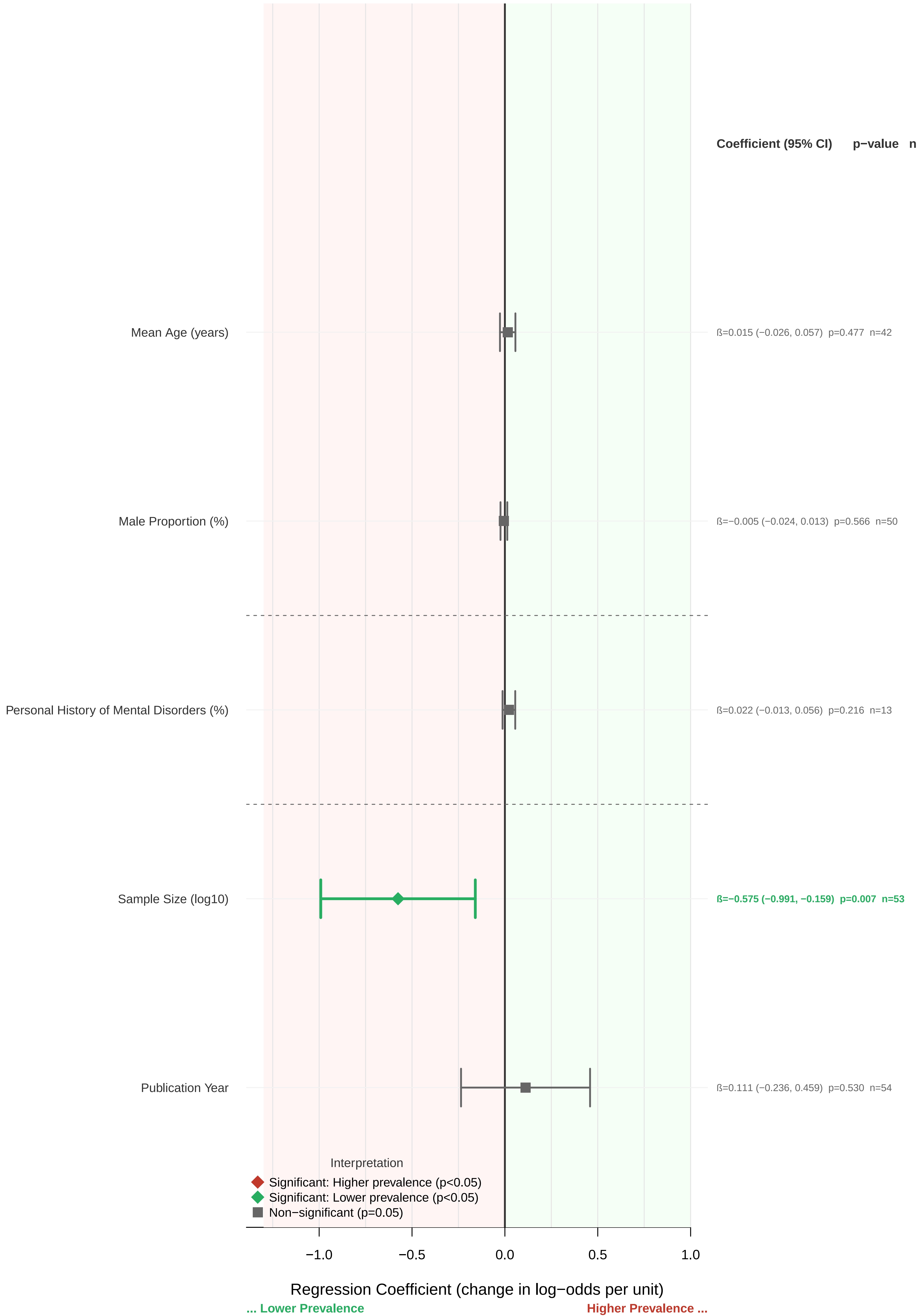

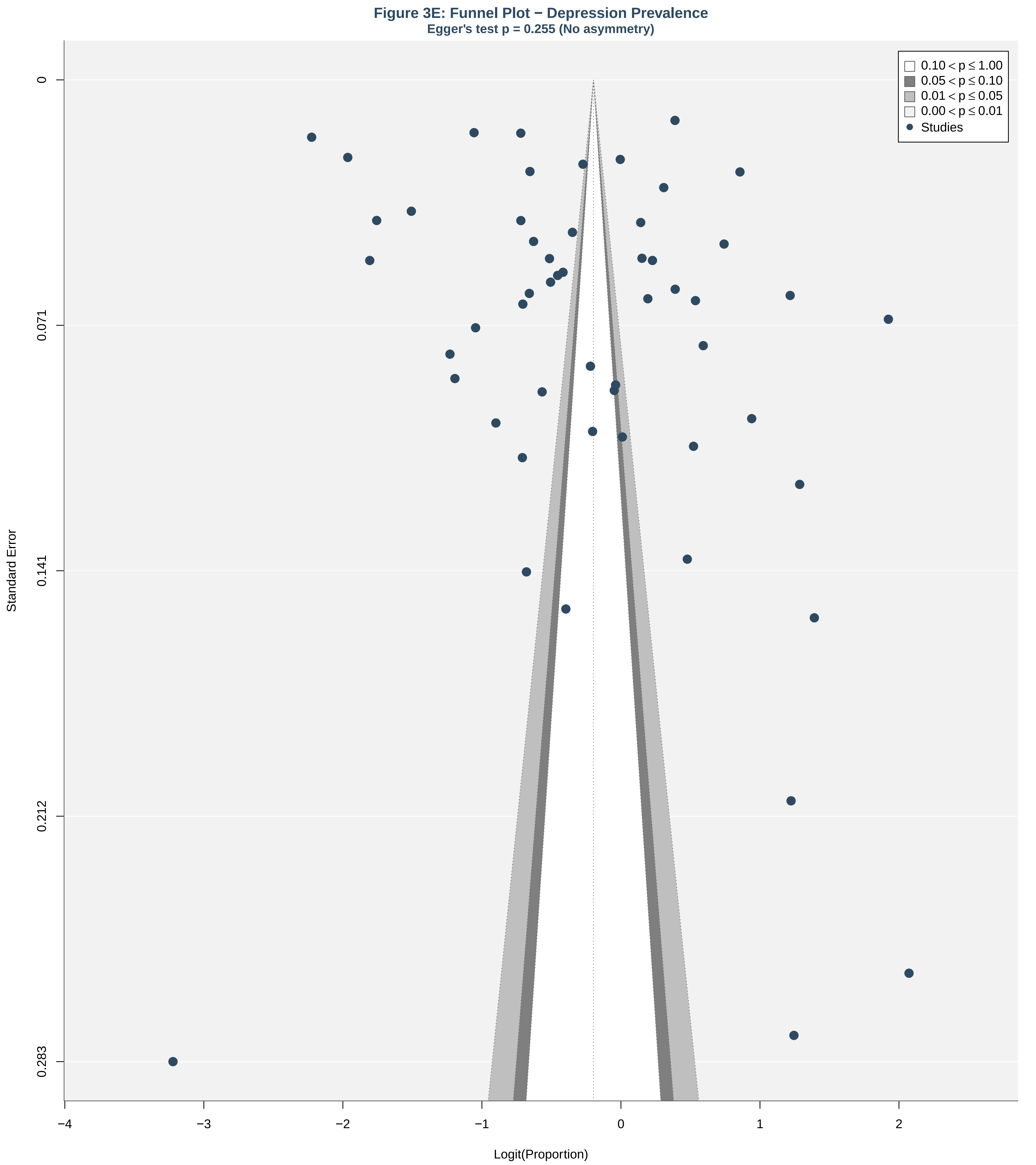

Figure 3E: Funnel Plot − Depression Prevalence
Egger's test p = 0.255 (No asymmetry)
0.10 < p ≤ 1.00
0.05 < p ≤ 0.10
0.01 < p ≤ 0.05
0.00 < p ≤ 0.01
Studies
Standard Error
0
0.071
0.141
0.212
0.283
−4
−3
−2
−1
0
1
2
Logit(Proportion)

Figure 4A: Forest Plot − Anxiety Prevalence

N = 54 studies

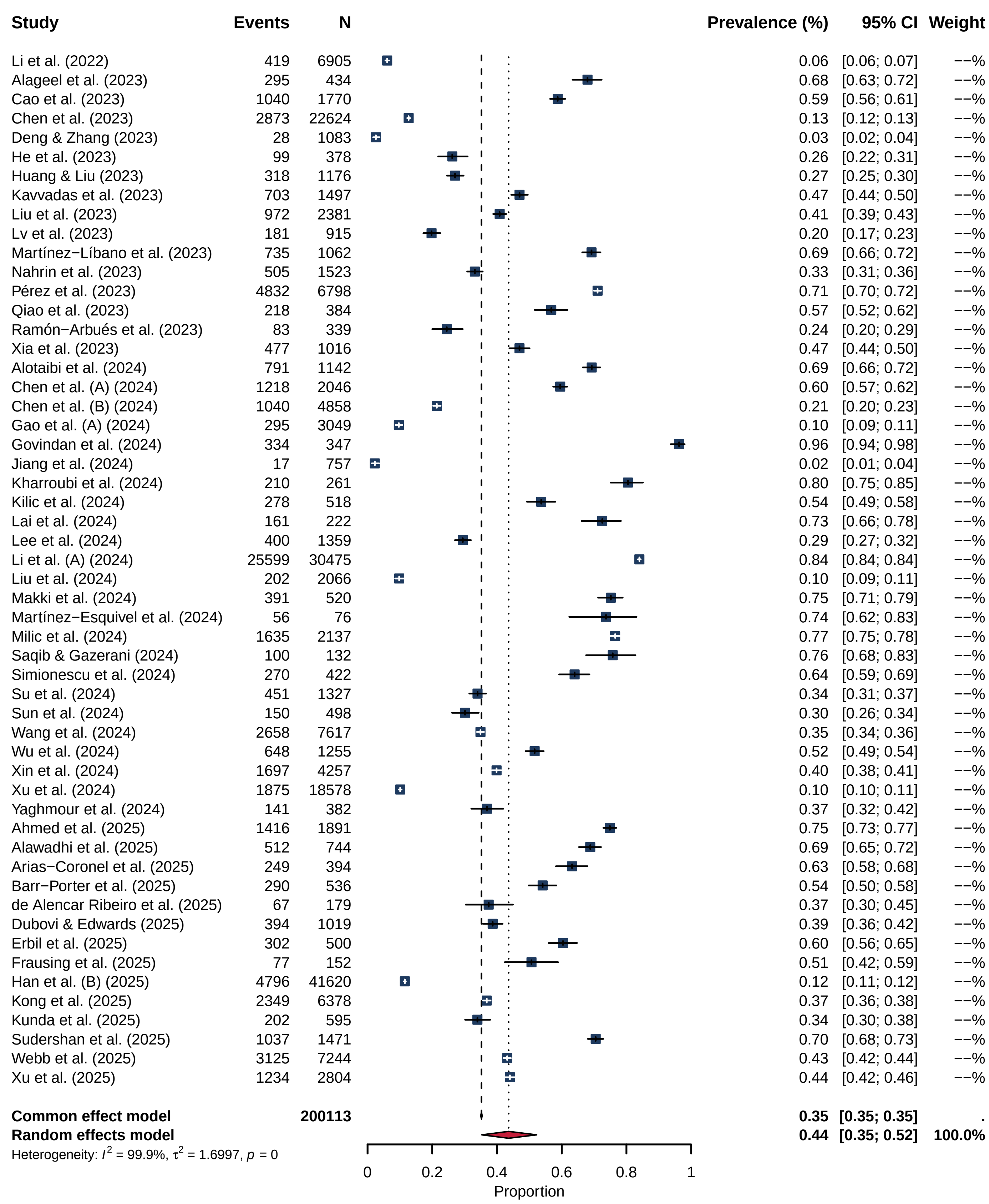

Figure 4B: Leave−One−Out Analysis − Anxiety Prevalence

Pooled estimate after omitting each study

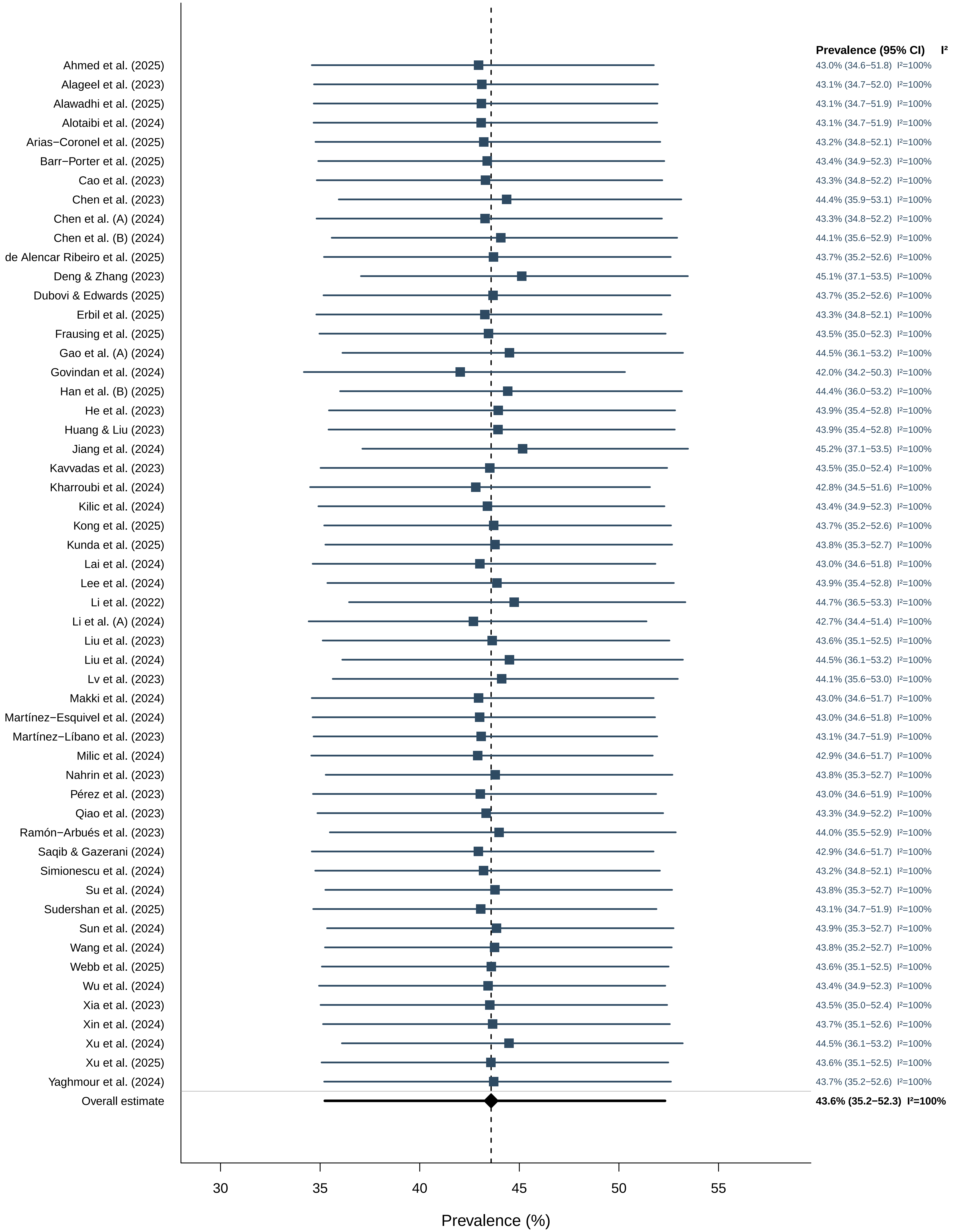

Figure 4C: Subgroup Analysis − Anxiety Prevalence

Stratified by study characteristics

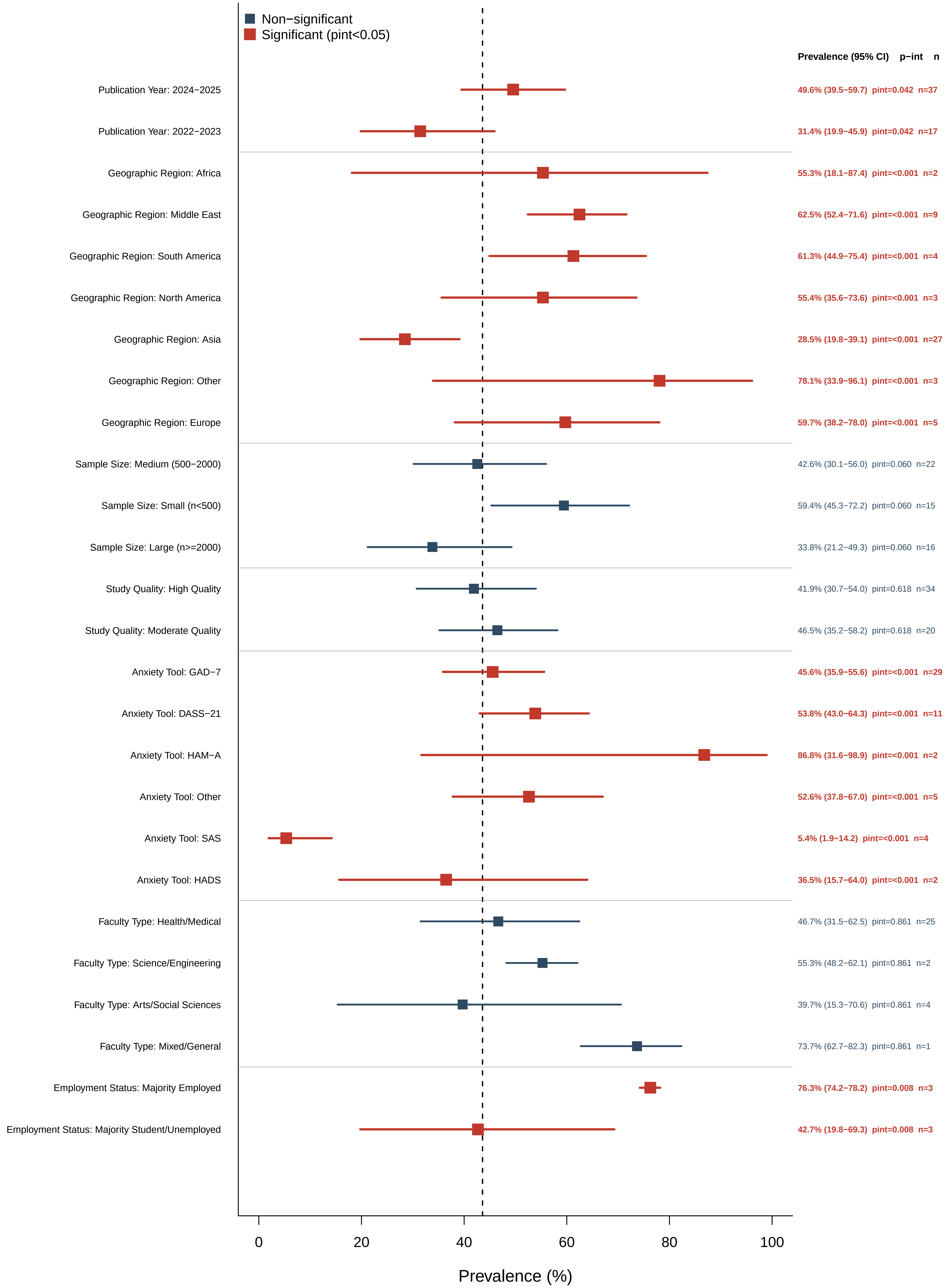

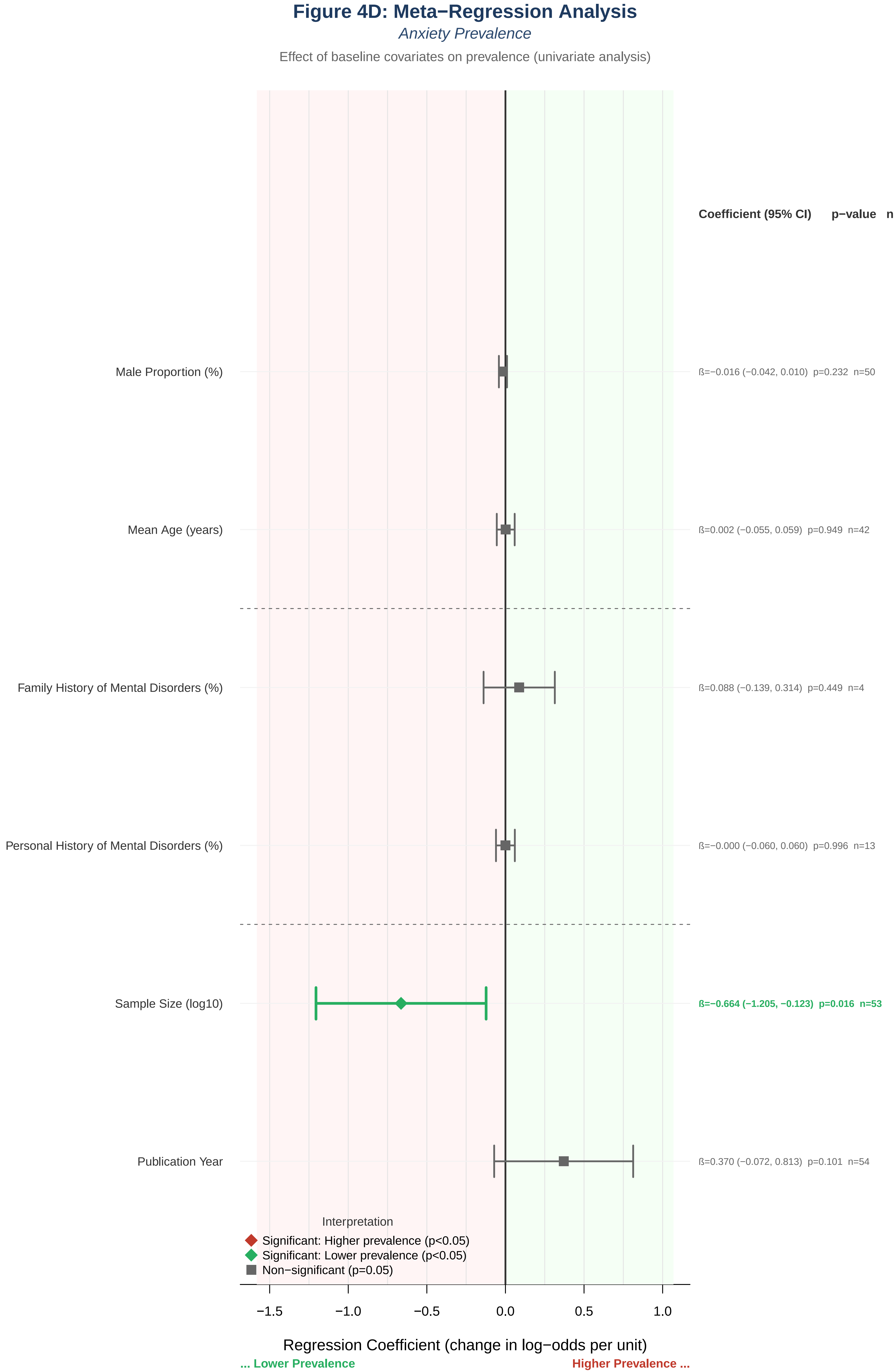

Figure 4D: Meta−Regression Analysis
Anxiety Prevalence
Effect of baseline covariates on prevalence (univariate analysis)
Coefficient (95% CI) p−value n
Male Proportion (%)
ß=−0.016 (−0.042, 0.010) p=0.232 n=50
Mean Age (years)
ß=0.002 (−0.055, 0.059) p=0.949 n=42
Family History of Mental Disorders (%)
ß=0.088 (−0.139, 0.314) p=0.449 n=4
Personal History of Mental Disorders (%)
ß=−0.000 (−0.060, 0.060) p=0.996 n=13
Sample Size (log10)
ß=−0.664 (−1.205, −0.123) p=0.016 n=53
Publication Year
ß=0.370 (−0.072, 0.813) p=0.101 n=54
Interpretation
Significant: Higher prevalence (p<0.05)
Significant: Lower prevalence (p<0.05)
Non−significant (p=0.05)
−1.5
−1.0
−0.5
0.0
0.5
1.0
Regression Coefficient (change in log−odds per unit)
... Lower Prevalence
Higher Prevalence ...

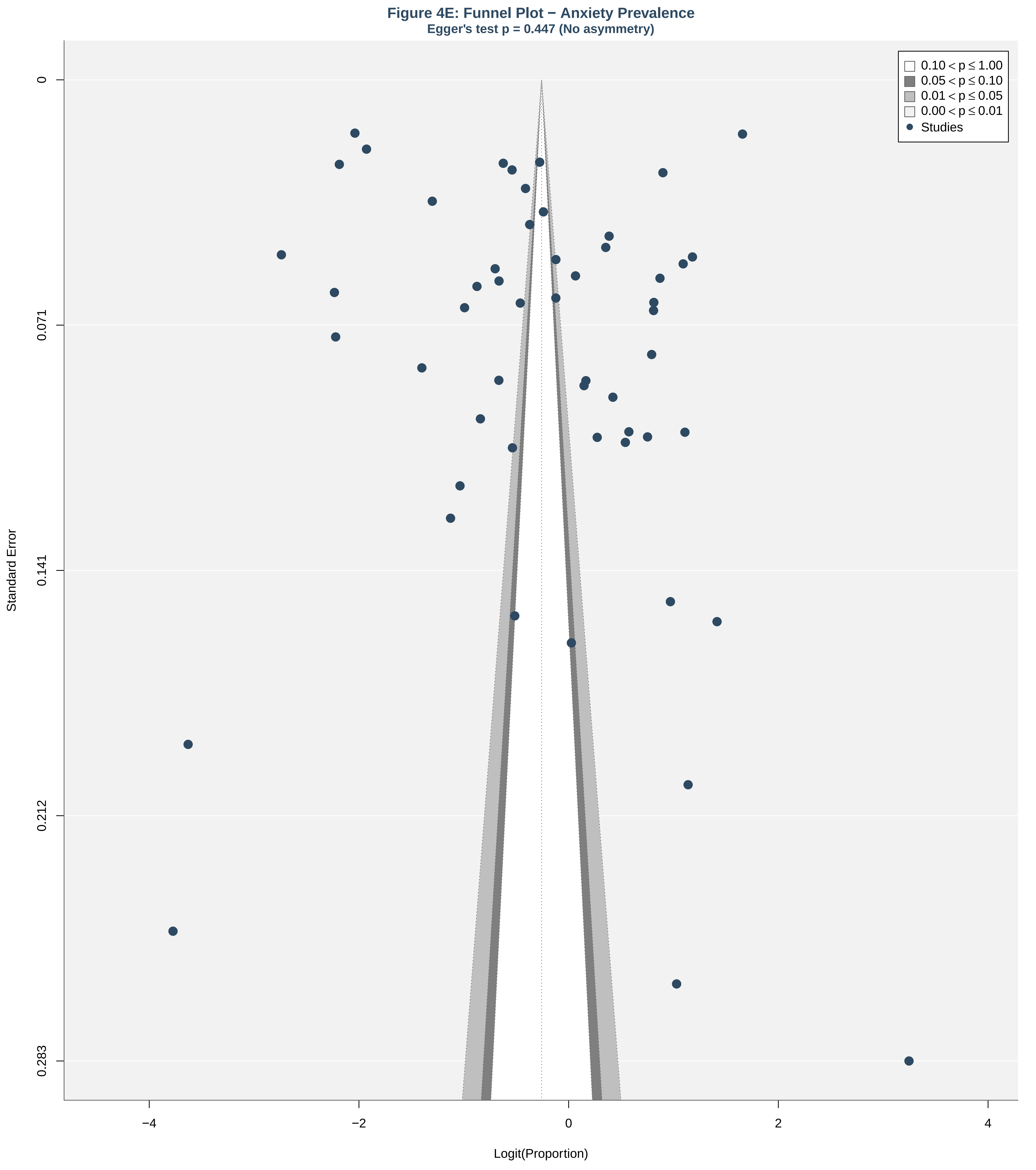

Figure 4E: Funnel Plot − Anxiety Prevalence
Egger's test p = 0.447 (No asymmetry)
0.10 < p ≤ 1.00
0.05 < p ≤ 0.10
0.01 < p ≤ 0.05
0.00 < p ≤ 0.01
Studies
Standard Error
0
0.071
0.141
0.212
0.283
Logit(Proportion)
−4
−2
0
2
4

Figure 5A: Forest Plot − Stress Prevalence

N = 26 studies

Study	Events	N	Prevalence (%)	95% CI	Weight
Cao et al. (2023)	639	1770	0.36	[0.34; 0.38]	−−%
Chen et al. (2023)	1787	22624	0.08	[0.08; 0.08]	−−%
Kavvadas et al. (2023)	708	1497	0.47	[0.45; 0.50]	−−%
Liu et al. (2023)	1327	2381	0.56	[0.54; 0.58]	−−%
Martínez−Líbano et al. (2023)	605	1062	0.57	[0.54; 0.60]	−−%
Rajasekhar et al. (2023)	509	2035	0.25	[0.23; 0.27]	−−%
Alotaibi et al. (2024)	588	1142	0.51	[0.49; 0.54]	−−%
Chen et al. (A) (2024)	509	2046	0.25	[0.23; 0.27]	−−%
Chen et al. (B) (2024)	451	4858	0.09	[0.08; 0.10]	−−%
Gao et al. (A) (2024)	645	3049	0.21	[0.20; 0.23]	−−%
Kharroubi et al. (2024)	231	261	0.89	[0.84; 0.92]	−−%
Kilic et al. (2024)	120	518	0.23	[0.20; 0.27]	−−%
Lai et al. (2024)	188	222	0.85	[0.79; 0.89]	−−%
Lee et al. (2024)	515	1359	0.38	[0.35; 0.41]	−−%
Li et al. (B) (2024)	4287	7845	0.55	[0.54; 0.56]	−−%
Li et al. (C) (2024)	478	7484	0.06	[0.06; 0.07]	−−%
Makki et al. (2024)	340	520	0.65	[0.61; 0.69]	−−%
Simionescu et al. (2024)	408	422	0.97	[0.94; 0.98]	−−%
Wu et al. (2024)	478	1255	0.38	[0.35; 0.41]	−−%
Xin et al. (2024)	1317	4257	0.31	[0.30; 0.32]	−−%
Yaghmour et al. (2024)	68	382	0.18	[0.14; 0.22]	−−%
Ahmed et al. (2025)	1194	1891	0.63	[0.61; 0.65]	−−%
de Alencar Ribeiro et al. (2025)	112	179	0.63	[0.55; 0.70]	−−%
Erbil et al. (2025)	191	500	0.38	[0.34; 0.43]	−−%
Frausing et al. (2025)	102	152	0.67	[0.59; 0.75]	−−%
Sudershan et al. (2025)	1270	1471	0.86	[0.84; 0.88]	−−%
**Common effect model**		**71182**	**0.27**	**[0.26; 0.27]**	**.**
**Random effects model**			**0.46**	**[0.33; 0.59]**	**100.0%**

Heterogeneity: I^2 = 99.8%, τ^2 = 1.9270, p = 0

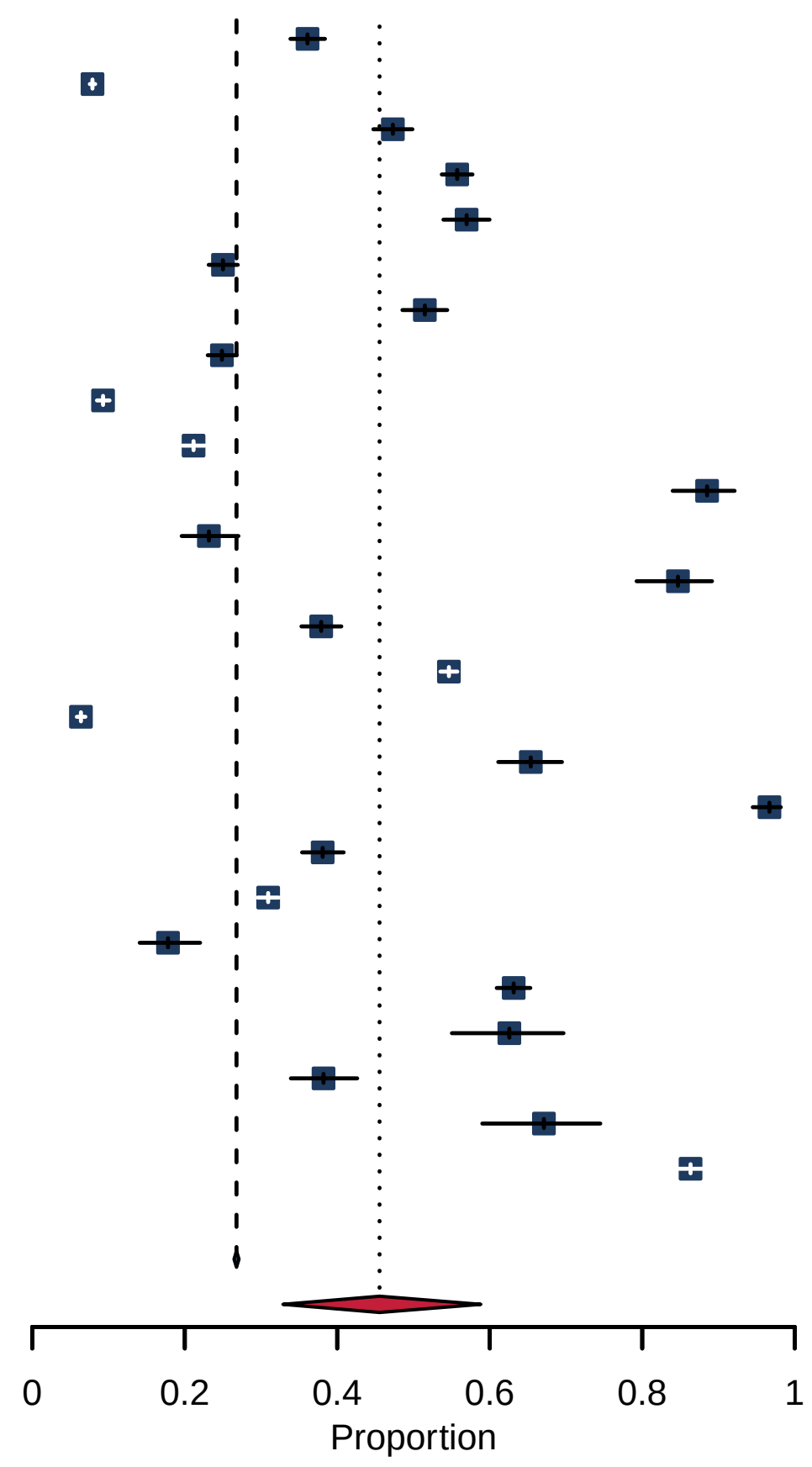

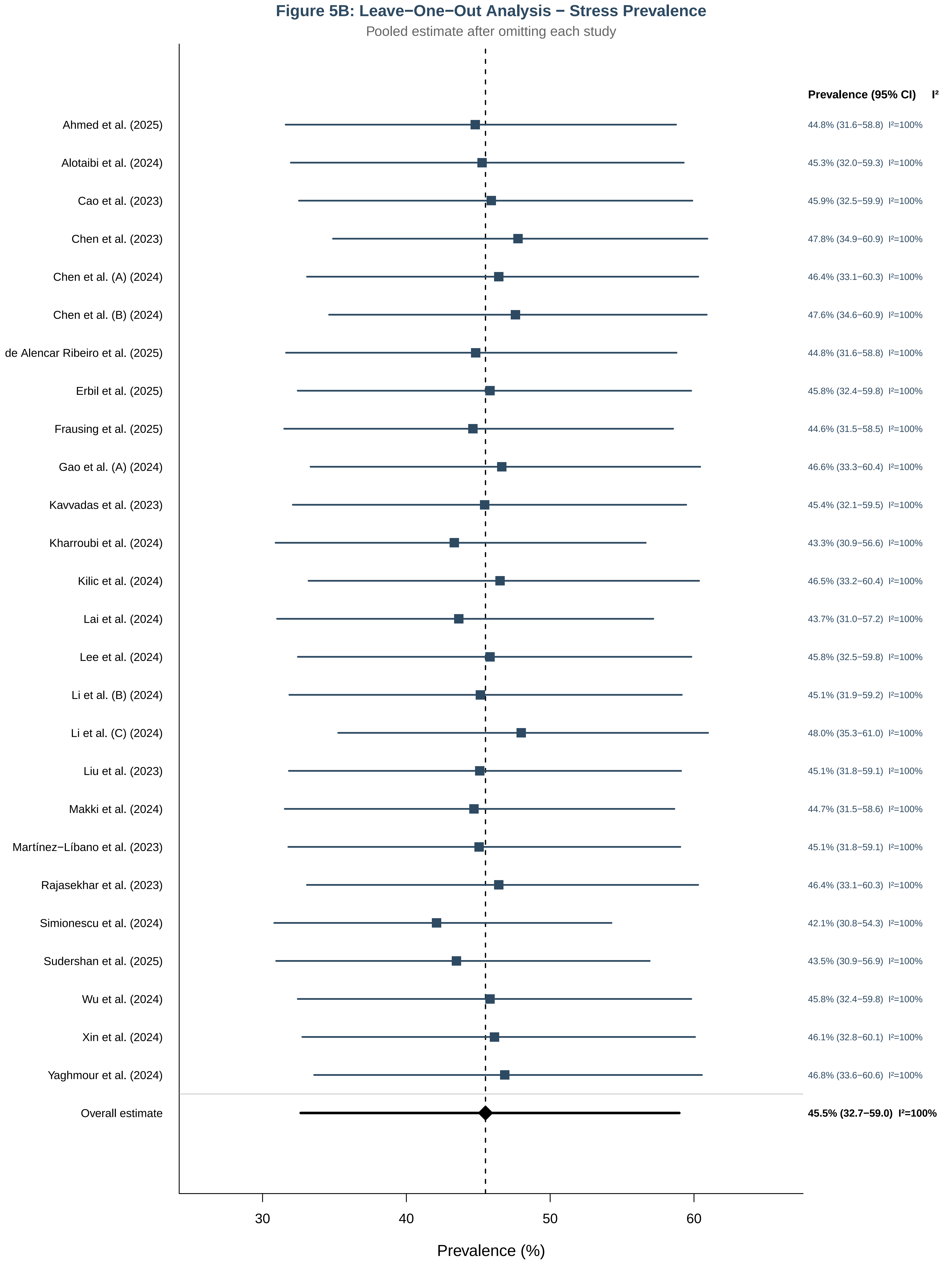

Figure 5B: Leave−One−Out Analysis − Stress Prevalence
Pooled estimate after omitting each study
Prevalence (95% CI) I²
Ahmed et al. (2025) 44.8% (31.6−58.8) I²=100%
Alotaibi et al. (2024) 45.3% (32.0−59.3) I²=100%
Cao et al. (2023) 45.9% (32.5−59.9) I²=100%
Chen et al. (2023) 47.8% (34.9−60.9) I²=100%
Chen et al. (A) (2024) 46.4% (33.1−60.3) I²=100%
Chen et al. (B) (2024) 47.6% (34.6−60.9) I²=100%
de Alencar Ribeiro et al. (2025) 44.8% (31.6−58.8) I²=100%
Erbil et al. (2025) 45.8% (32.4−59.8) I²=100%
Frausing et al. (2025) 44.6% (31.5−58.5) I²=100%
Gao et al. (A) (2024) 46.6% (33.3−60.4) I²=100%
Kavvadas et al. (2023) 45.4% (32.1−59.5) I²=100%
Kharroubi et al. (2024) 43.3% (30.9−56.6) I²=100%
Kilic et al. (2024) 46.5% (33.2−60.4) I²=100%
Lai et al. (2024) 43.7% (31.0−57.2) I²=100%
Lee et al. (2024) 45.8% (32.5−59.8) I²=100%
Li et al. (B) (2024) 45.1% (31.9−59.2) I²=100%
Li et al. (C) (2024) 48.0% (35.3−61.0) I²=100%
Liu et al. (2023) 45.1% (31.8−59.1) I²=100%
Makki et al. (2024) 44.7% (31.5−58.6) I²=100%
Martínez−Líbano et al. (2023) 45.1% (31.8−59.1) I²=100%
Rajasekhar et al. (2023) 46.4% (33.1−60.3) I²=100%
Simionescu et al. (2024) 42.1% (30.8−54.3) I²=100%
Sudershan et al. (2025) 43.5% (30.9−56.9) I²=100%
Wu et al. (2024) 45.8% (32.4−59.8) I²=100%
Xin et al. (2024) 46.1% (32.8−60.1) I²=100%
Yaghmour et al. (2024) 46.8% (33.6−60.6) I²=100%
Overall estimate 45.5% (32.7−59.0) I²=100%
30
40
50
60
Prevalence (%)

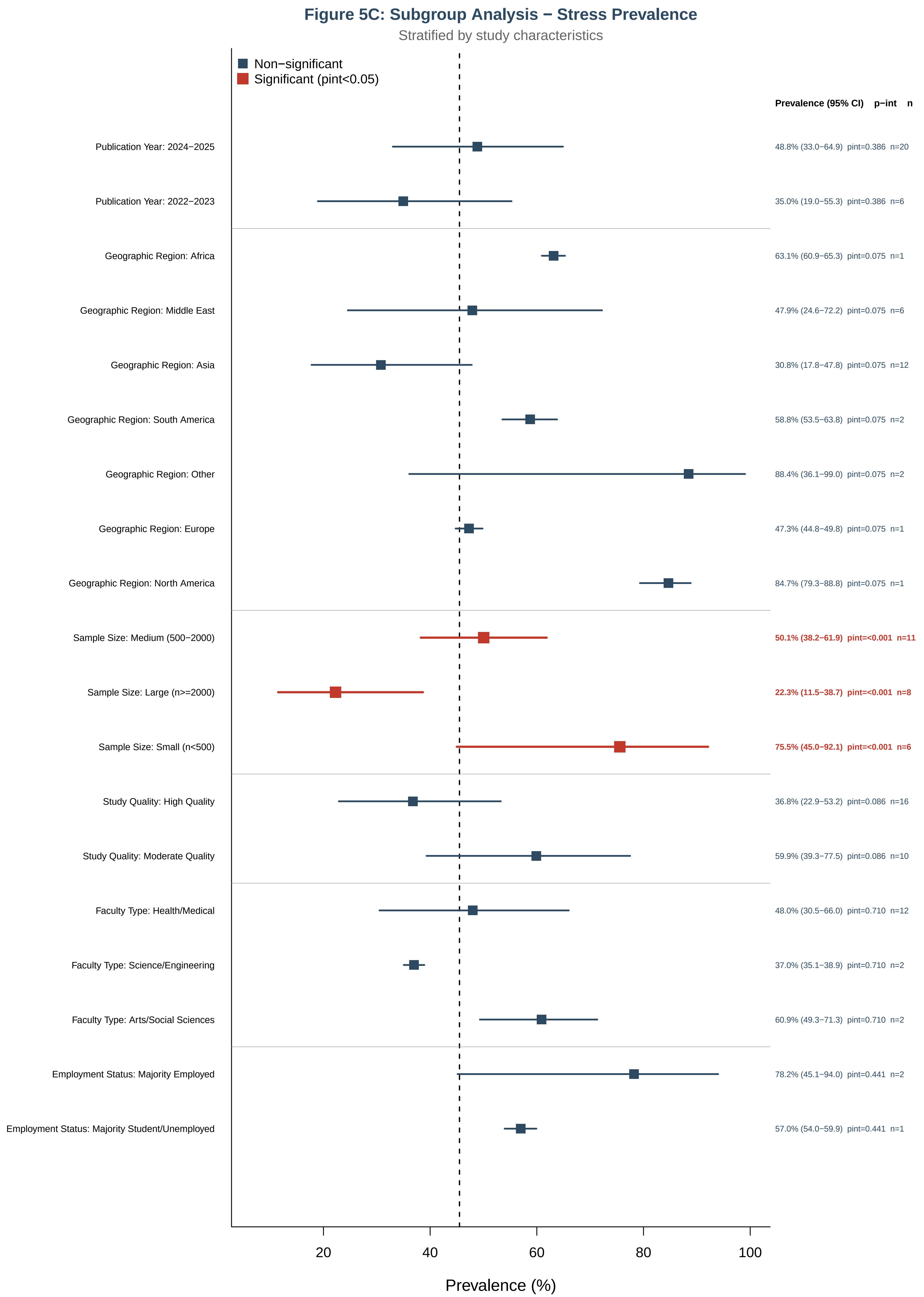
Figure 5C: Subgroup Analysis − Stress Prevalence
Stratified by study characteristics
Non−significant
Significant (pint<0.05)
Prevalence (95% CI) p−int n
Publication Year: 2024−2025
48.8% (33.0−64.9) pint=0.386 n=20
Publication Year: 2022−2023
35.0% (19.0−55.3) pint=0.386 n=6
Geographic Region: Africa
63.1% (60.9−65.3) pint=0.075 n=1
Geographic Region: Middle East
47.9% (24.6−72.2) pint=0.075 n=6
Geographic Region: Asia
30.8% (17.8−47.8) pint=0.075 n=12
Geographic Region: South America
58.8% (53.5−63.8) pint=0.075 n=2
Geographic Region: Other
88.4% (36.1−99.0) pint=0.075 n=2
Geographic Region: Europe
47.3% (44.8−49.8) pint=0.075 n=1
Geographic Region: North America
84.7% (79.3−88.8) pint=0.075 n=1
Sample Size: Medium (500−2000)
50.1% (38.2−61.9) pint=<0.001 n=11
Sample Size: Large (n>=2000)
22.3% (11.5−38.7) pint=<0.001 n=8
Sample Size: Small (n<500)
75.5% (45.0−92.1) pint=<0.001 n=6
Study Quality: High Quality
36.8% (22.9−53.2) pint=0.086 n=16
Study Quality: Moderate Quality
59.9% (39.3−77.5) pint=0.086 n=10
Faculty Type: Health/Medical
48.0% (30.5−66.0) pint=0.710 n=12
Faculty Type: Science/Engineering
37.0% (35.1−38.9) pint=0.710 n=2
Faculty Type: Arts/Social Sciences
60.9% (49.3−71.3) pint=0.710 n=2
Employment Status: Majority Employed
78.2% (45.1−94.0) pint=0.441 n=2
Employment Status: Majority Student/Unemployed
57.0% (54.0−59.9) pint=0.441 n=1
20
40
60
80
100
Prevalence (%)

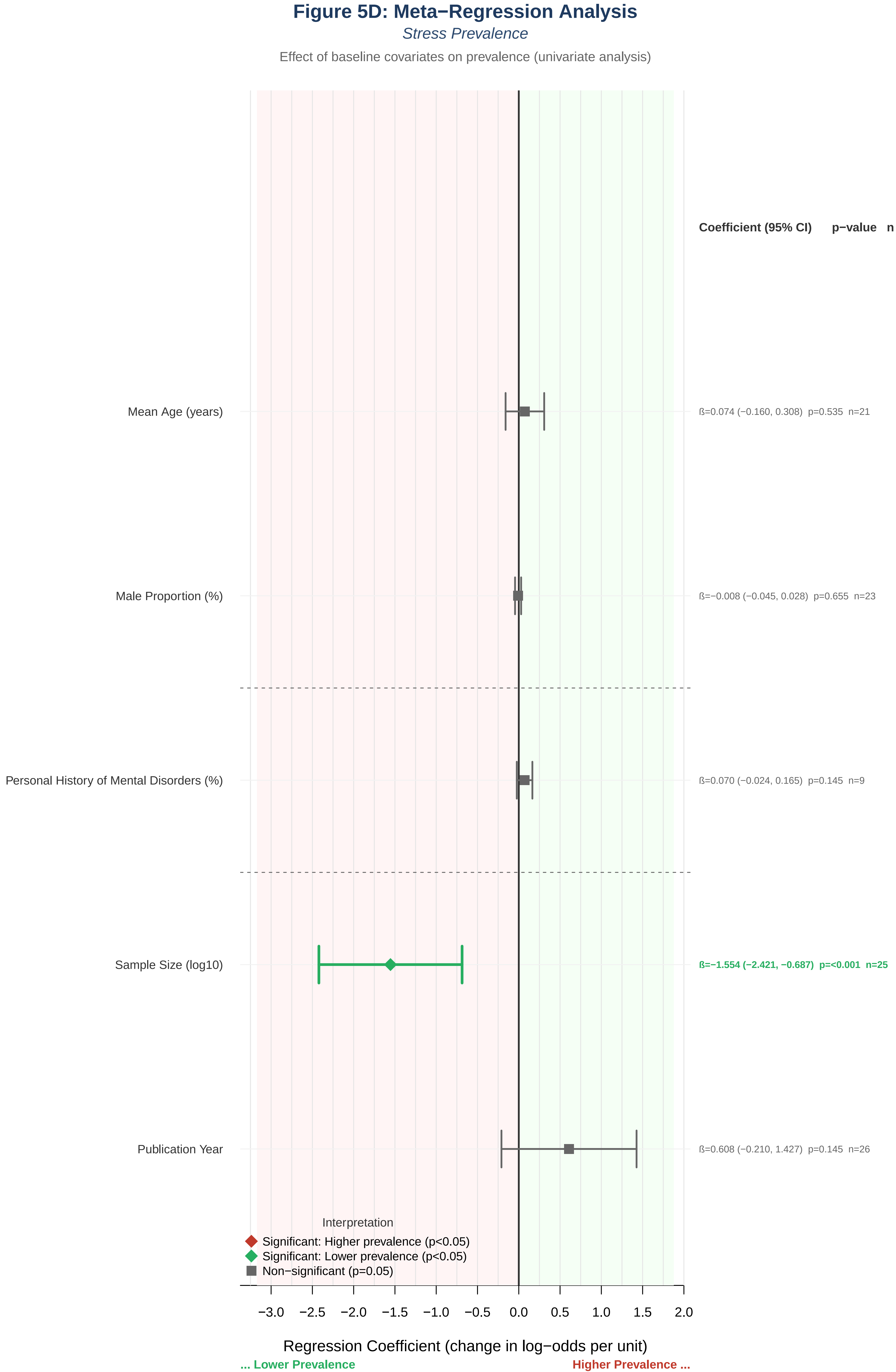

Figure 5D: Meta−Regression Analysis
Stress Prevalence
Effect of baseline covariates on prevalence (univariate analysis)
Coefficient (95% CI) p−value n
Mean Age (years)
ß=0.074 (−0.160, 0.308) p=0.535 n=21
Male Proportion (%)
ß=−0.008 (−0.045, 0.028) p=0.655 n=23
Personal History of Mental Disorders (%)
ß=0.070 (−0.024, 0.165) p=0.145 n=9
Sample Size (log10)
ß=−1.554 (−2.421, −0.687) p=<0.001 n=25
Publication Year
ß=0.608 (−0.210, 1.427) p=0.145 n=26
Interpretation
Significant: Higher prevalence (p<0.05)
Significant: Lower prevalence (p<0.05)
Non−significant (p=0.05)
−3.0
−2.5
−2.0
−1.5
−1.0
−0.5
0.0
0.5
1.0
1.5
2.0
Regression Coefficient (change in log−odds per unit)
... Lower Prevalence
Higher Prevalence ...

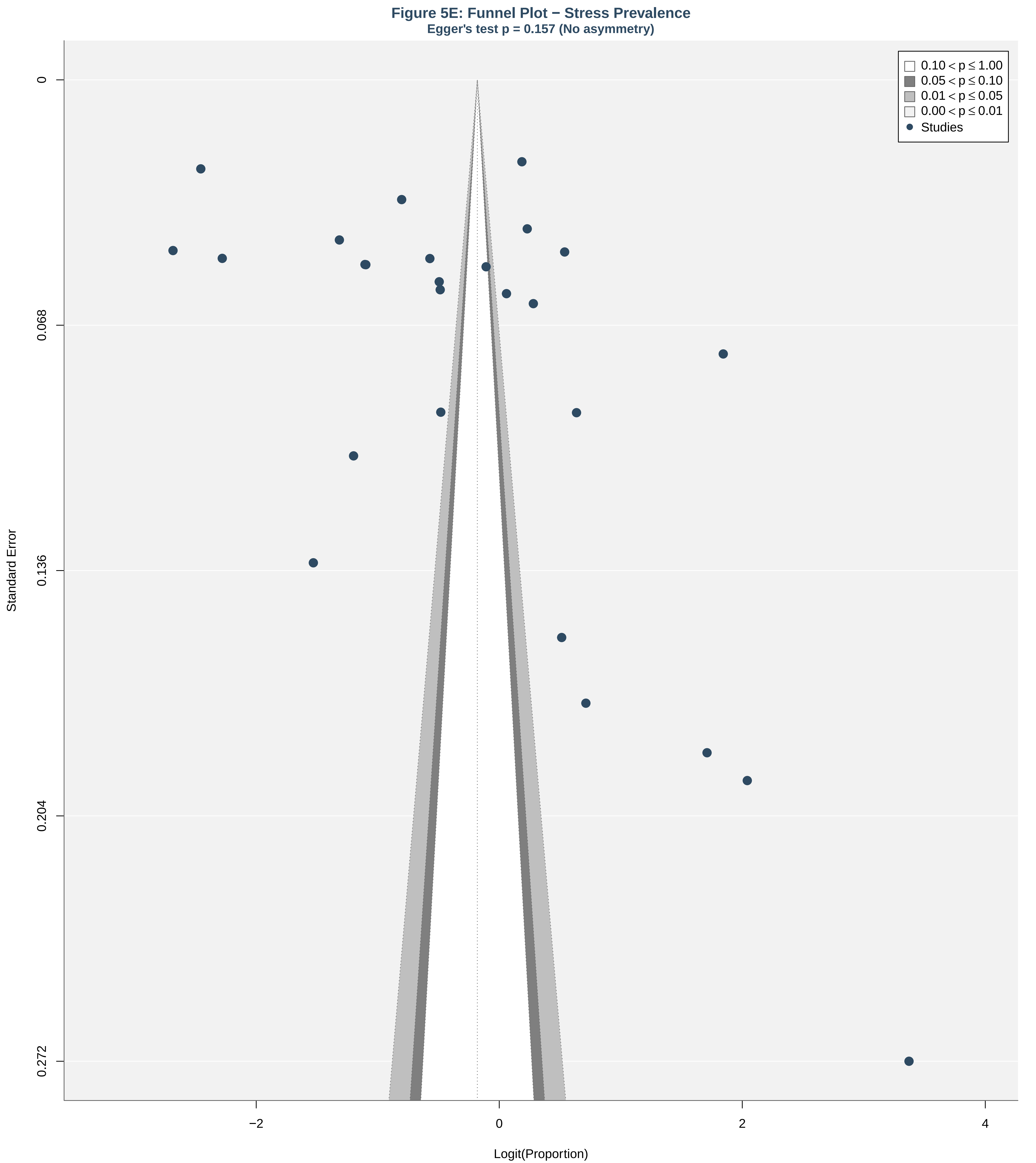
Figure 5E: Funnel Plot − Stress Prevalence
Egger's test p = 0.157 (No asymmetry)
0.10 < p ≤ 1.00
0.05 < p ≤ 0.10
0.01 < p ≤ 0.05
0.00 < p ≤ 0.01
Studies
Standard Error
0
0.068
0.136
0.204
0.272
−2
0
2
4
Logit(Proportion)

Figure 6A: Forest Plot − Mild Depression Prevalence

N = 32 studies

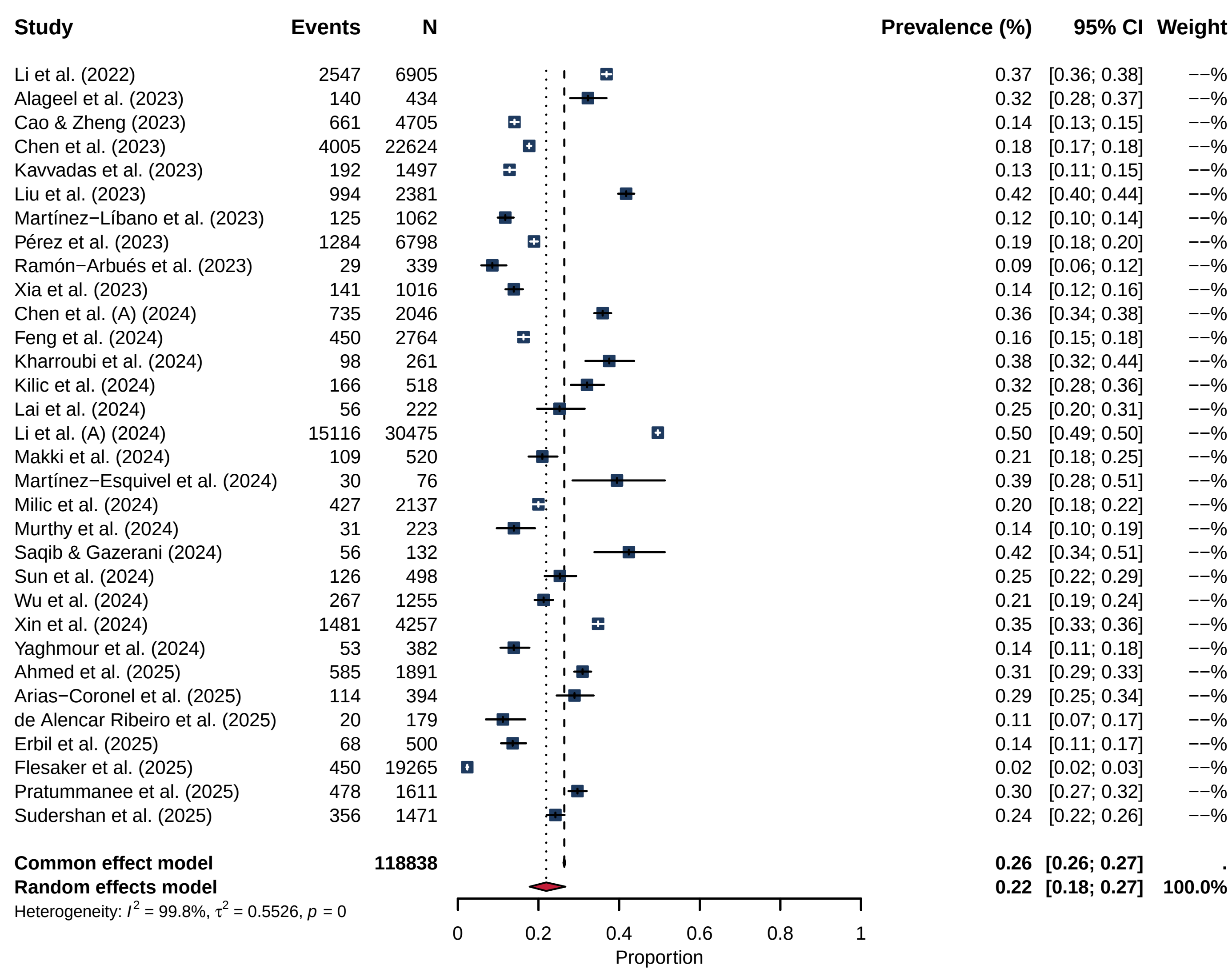

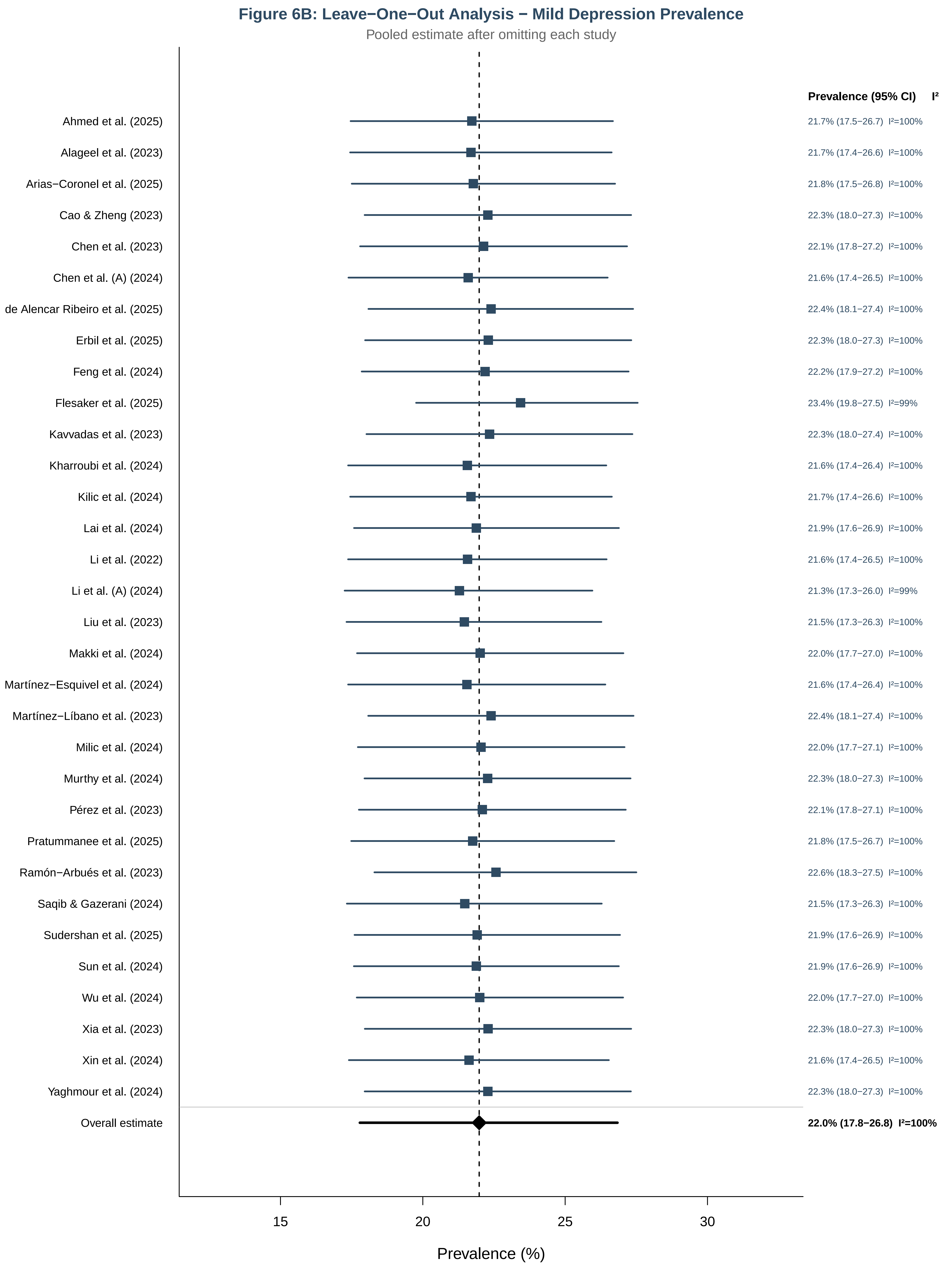

Figure 6B: Leave−One−Out Analysis − Mild Depression Prevalence
Pooled estimate after omitting each study
Prevalence (95% CI) I²
Ahmed et al. (2025) 21.7% (17.5−26.7) I²=100%
Alageel et al. (2023) 21.7% (17.4−26.6) I²=100%
Arias−Coronel et al. (2025) 21.8% (17.5−26.8) I²=100%
Cao & Zheng (2023) 22.3% (18.0−27.3) I²=100%
Chen et al. (2023) 22.1% (17.8−27.2) I²=100%
Chen et al. (A) (2024) 21.6% (17.4−26.5) I²=100%
de Alencar Ribeiro et al. (2025) 22.4% (18.1−27.4) I²=100%
Erbil et al. (2025) 22.3% (18.0−27.3) I²=100%
Feng et al. (2024) 22.2% (17.9−27.2) I²=100%
Flesaker et al. (2025) 23.4% (19.8−27.5) I²=99%
Kavvadas et al. (2023) 22.3% (18.0−27.4) I²=100%
Kharroubi et al. (2024) 21.6% (17.4−26.4) I²=100%
Kilic et al. (2024) 21.7% (17.4−26.6) I²=100%
Lai et al. (2024) 21.9% (17.6−26.9) I²=100%
Li et al. (2022) 21.6% (17.4−26.5) I²=100%
Li et al. (A) (2024) 21.3% (17.3−26.0) I²=99%
Liu et al. (2023) 21.5% (17.3−26.3) I²=100%
Makki et al. (2024) 22.0% (17.7−27.0) I²=100%
Martínez−Esquivel et al. (2024) 21.6% (17.4−26.4) I²=100%
Martínez−Líbano et al. (2023) 22.4% (18.1−27.4) I²=100%
Milic et al. (2024) 22.0% (17.7−27.1) I²=100%
Murthy et al. (2024) 22.3% (18.0−27.3) I²=100%
Pérez et al. (2023) 22.1% (17.8−27.1) I²=100%
Pratummanee et al. (2025) 21.8% (17.5−26.7) I²=100%
Ramón−Arbués et al. (2023) 22.6% (18.3−27.5) I²=100%
Saqib & Gazerani (2024) 21.5% (17.3−26.3) I²=100%
Sudershan et al. (2025) 21.9% (17.6−26.9) I²=100%
Sun et al. (2024) 21.9% (17.6−26.9) I²=100%
Wu et al. (2024) 22.0% (17.7−27.0) I²=100%
Xia et al. (2023) 22.3% (18.0−27.3) I²=100%
Xin et al. (2024) 21.6% (17.4−26.5) I²=100%
Yaghmour et al. (2024) 22.3% (18.0−27.3) I²=100%
Overall estimate 22.0% (17.8−26.8) I²=100%
15
20
25
30
Prevalence (%)

Figure 6C: Subgroup Analysis − Mild Depression Prevalence

Stratified by study characteristics

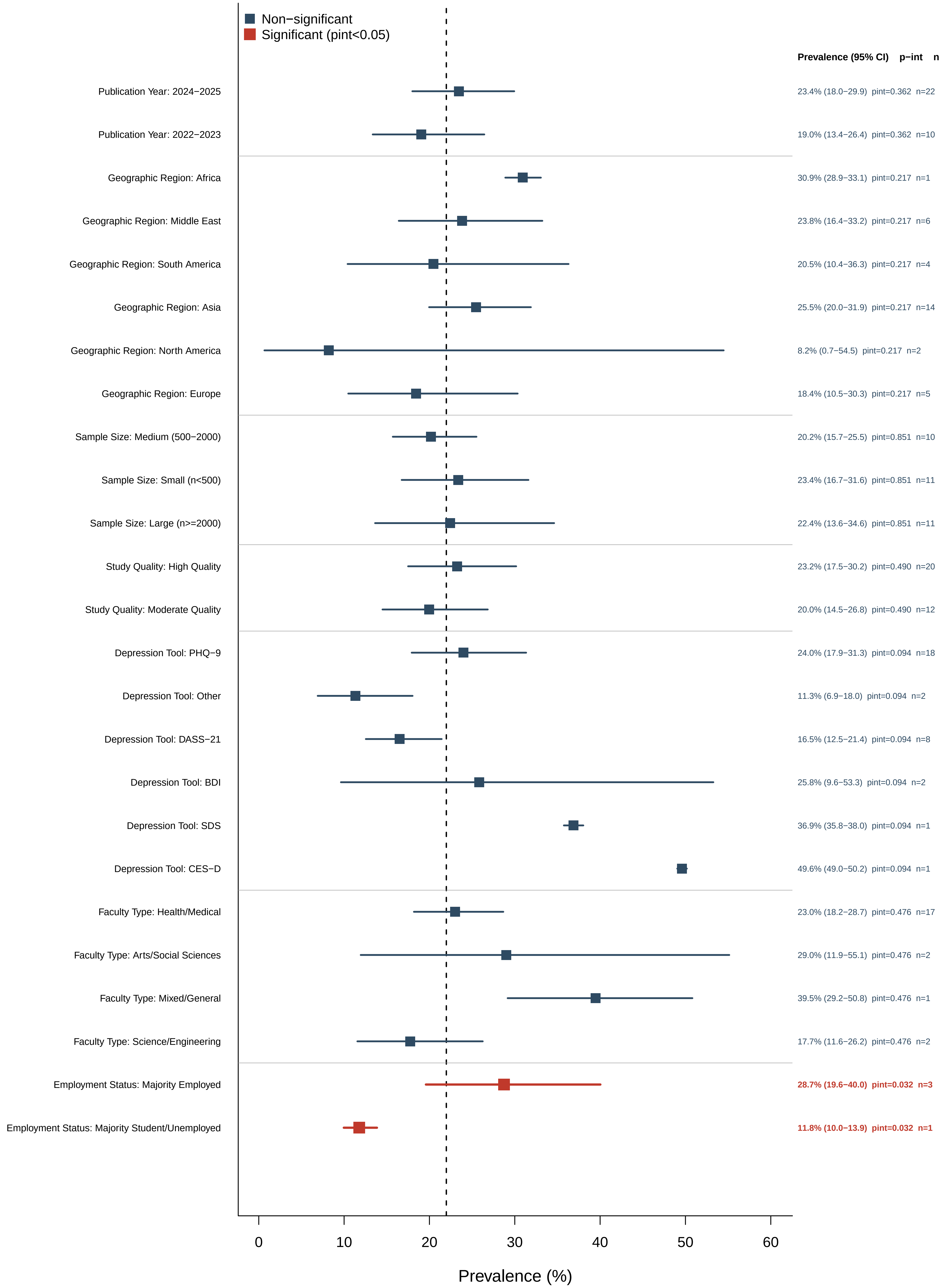

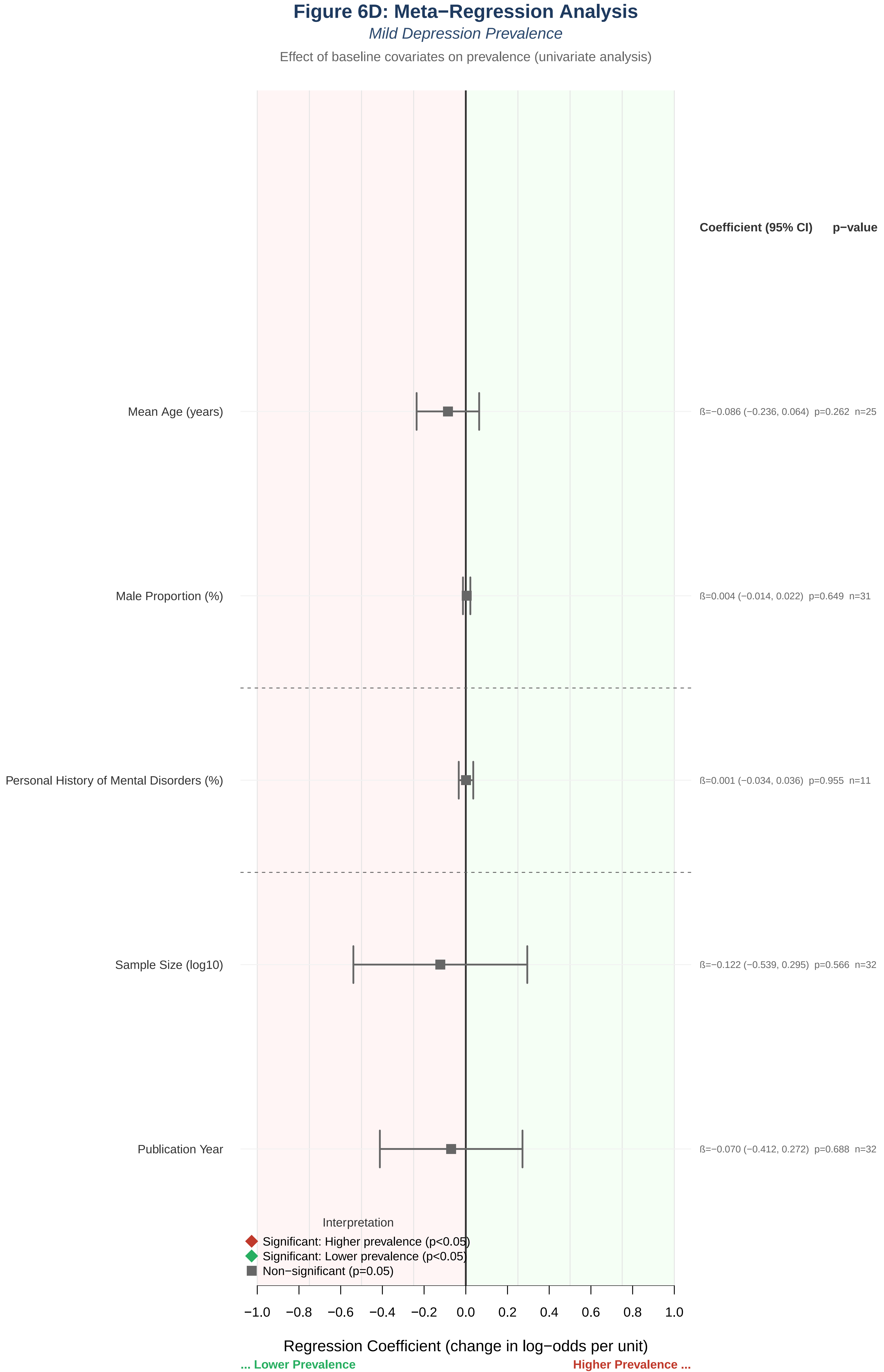
Figure 6D: Meta−Regression Analysis
Mild Depression Prevalence
Effect of baseline covariates on prevalence (univariate analysis)
Coefficient (95% CI) p−value n
Mean Age (years)
ß=−0.086 (−0.236, 0.064) p=0.262 n=25
Male Proportion (%)
ß=0.004 (−0.014, 0.022) p=0.649 n=31
Personal History of Mental Disorders (%)
ß=0.001 (−0.034, 0.036) p=0.955 n=11
Sample Size (log10)
ß=−0.122 (−0.539, 0.295) p=0.566 n=32
Publication Year
ß=−0.070 (−0.412, 0.272) p=0.688 n=32
Interpretation
Significant: Higher prevalence (p<0.05)
Significant: Lower prevalence (p<0.05)
Non−significant (p=0.05)
−1.0 −0.8 −0.6 −0.4 −0.2 0.0 0.2 0.4 0.6 0.8 1.0
Regression Coefficient (change in log−odds per unit)
... Lower Prevalence
Higher Prevalence ...

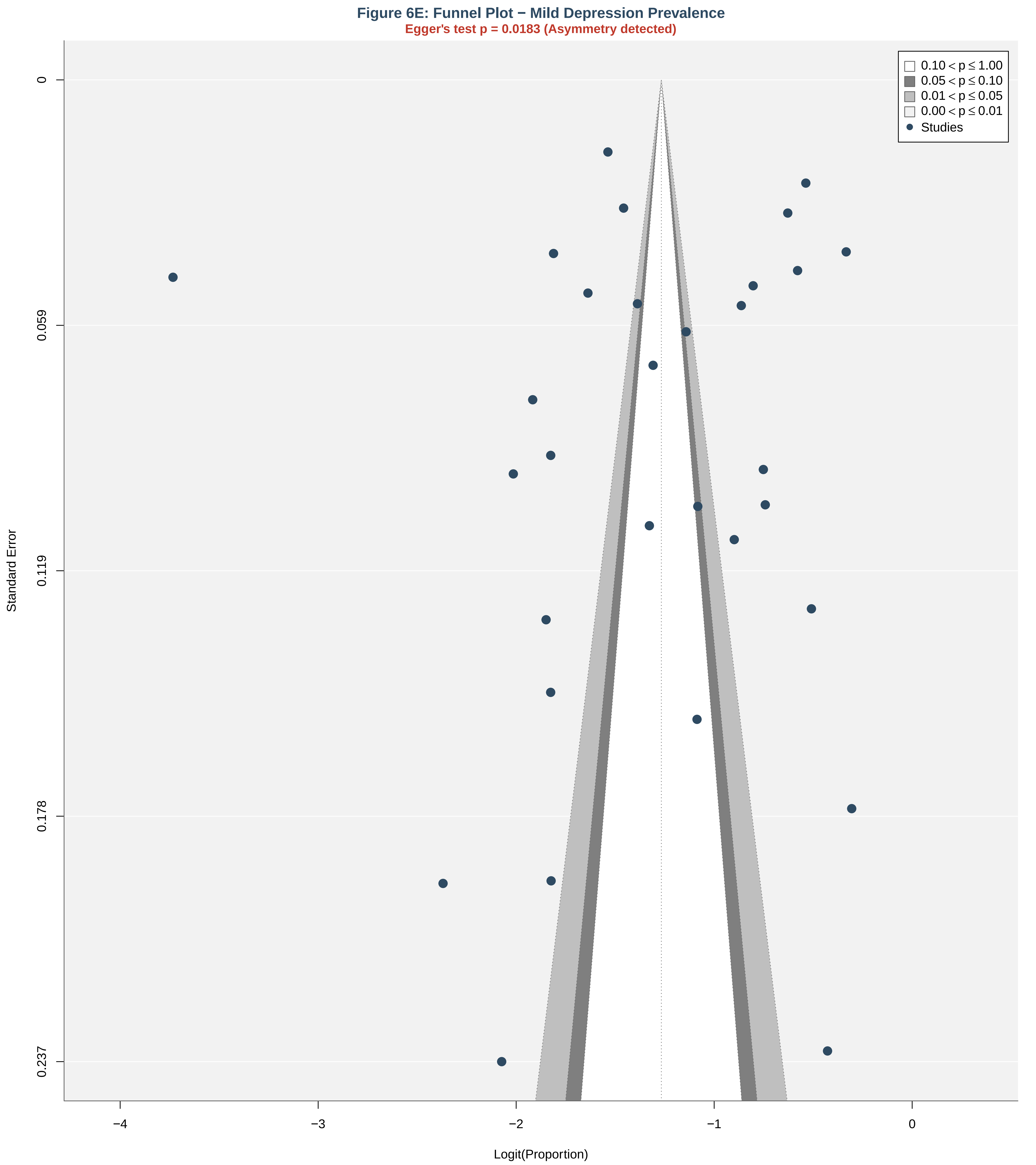

Figure 6E: Funnel Plot − Mild Depression Prevalence
Egger's test p = 0.0183 (Asymmetry detected)
0.10 < p ≤ 1.00
0.05 < p ≤ 0.10
0.01 < p ≤ 0.05
0.00 < p ≤ 0.01
Studies
Standard Error
0
0.059
0.119
0.178
0.237
−4
−3
−2
−1
0
Logit(Proportion)

Figure 7A: Forest Plot − Moderate Depression Prevalence

N = 31 studies

Study	Events	N	Prevalence (%)	95% CI	Weight
Li et al. (2022)	430	6905	0.06	[0.06; 0.07]	−−%
Alageel et al. (2023)	110	434	0.25	[0.21; 0.30]	−−%
Cao & Zheng (2023)	130	4705	0.03	[0.02; 0.03]	−−%
Chen et al. (2023)	1201	22624	0.05	[0.05; 0.06]	−−%
Deng & Zhang (2023)	51	1083	0.05	[0.04; 0.06]	−−%
He et al. (2023)	66	378	0.17	[0.14; 0.22]	−−%
Kavvadas et al. (2023)	265	1497	0.18	[0.16; 0.20]	−−%
Martínez−Líbano et al. (2023)	232	1062	0.22	[0.19; 0.24]	−−%
Pérez et al. (2023)	1606	6798	0.24	[0.23; 0.25]	−−%
Xia et al. (2023)	53	1016	0.05	[0.04; 0.07]	−−%
Chen et al. (A) (2024)	437	2046	0.21	[0.20; 0.23]	−−%
Feng et al. (2024)	330	2764	0.12	[0.11; 0.13]	−−%
Kharroubi et al. (2024)	51	261	0.20	[0.15; 0.25]	−−%
Kilic et al. (2024)	88	518	0.17	[0.14; 0.21]	−−%
Lai et al. (2024)	30	222	0.14	[0.09; 0.19]	−−%
Martínez−Esquivel et al. (2024)	16	76	0.21	[0.13; 0.32]	−−%
Milic et al. (2024)	246	2137	0.12	[0.10; 0.13]	−−%
Murthy et al. (2024)	22	223	0.10	[0.06; 0.15]	−−%
Saqib & Gazerani (2024)	26	132	0.20	[0.13; 0.28]	−−%
Sun et al. (2024)	17	498	0.03	[0.02; 0.05]	−−%
Wu et al. (2024)	143	1255	0.11	[0.10; 0.13]	−−%
Xin et al. (2024)	493	4257	0.12	[0.11; 0.13]	−−%
Yaghmour et al. (2024)	59	382	0.15	[0.12; 0.19]	−−%
Ahmed et al. (2025)	538	1891	0.28	[0.26; 0.31]	−−%
Arias−Coronel et al. (2025)	91	394	0.23	[0.19; 0.28]	−−%
de Alencar Ribeiro et al. (2025)	24	179	0.13	[0.09; 0.19]	−−%
Erbil et al. (2025)	99	500	0.20	[0.16; 0.24]	−−%
Flesaker et al. (2025)	330	19265	0.02	[0.02; 0.02]	−−%
Frausing et al. (2025)	32	152	0.21	[0.15; 0.28]	−−%
Pratummanee et al. (2025)	345	1611	0.21	[0.19; 0.24]	−−%
Sudershan et al. (2025)	318	1471	0.22	[0.20; 0.24]	−−%
**Common effect model**		**86736**	**0.09**	**[0.09; 0.09]**	**.**
**Random effects model**			**0.13**	**[0.10; 0.16]**	**100.0%**

Heterogeneity: $I^2 = 99.4\%$, $\tau^2 = 0.6157$, $p = 0$

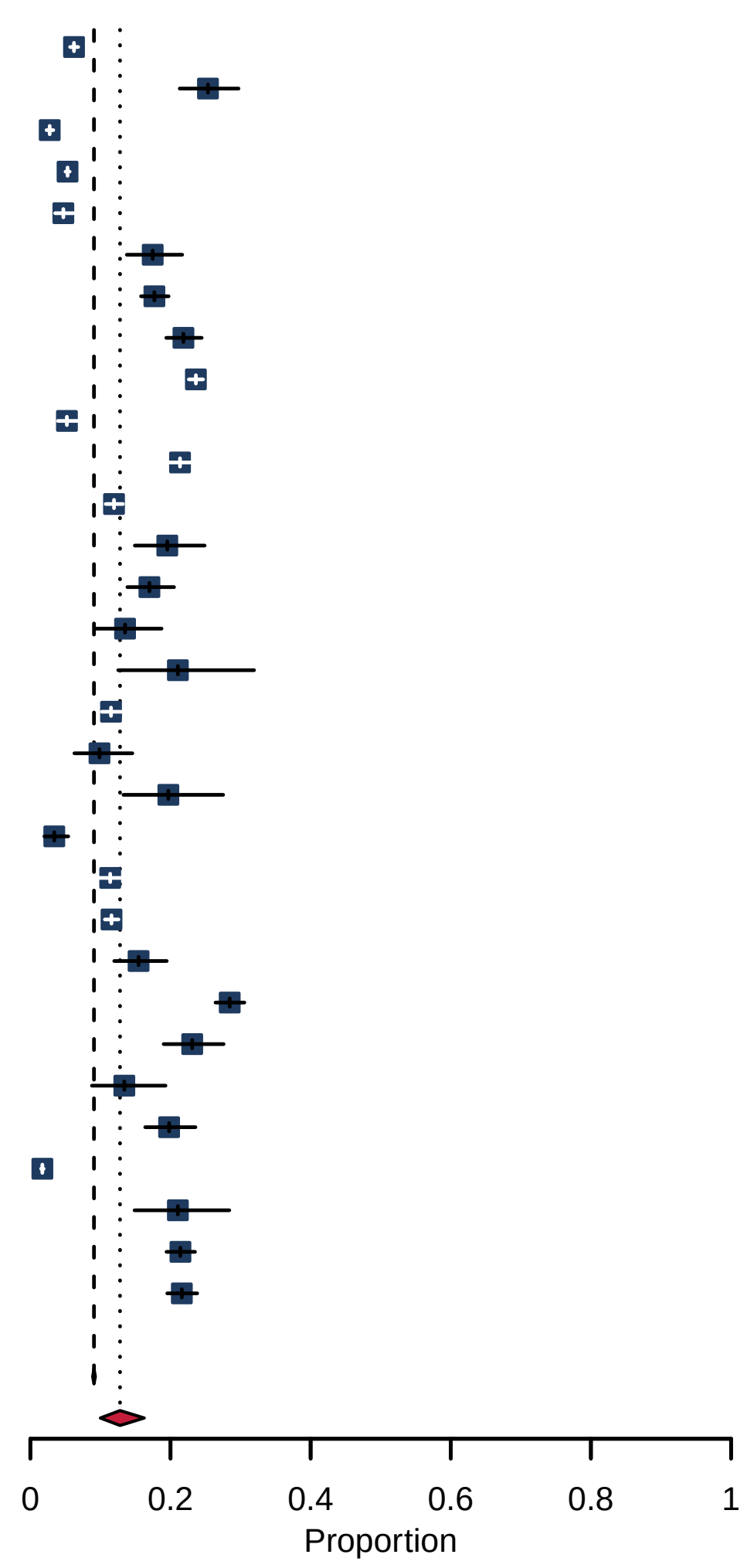

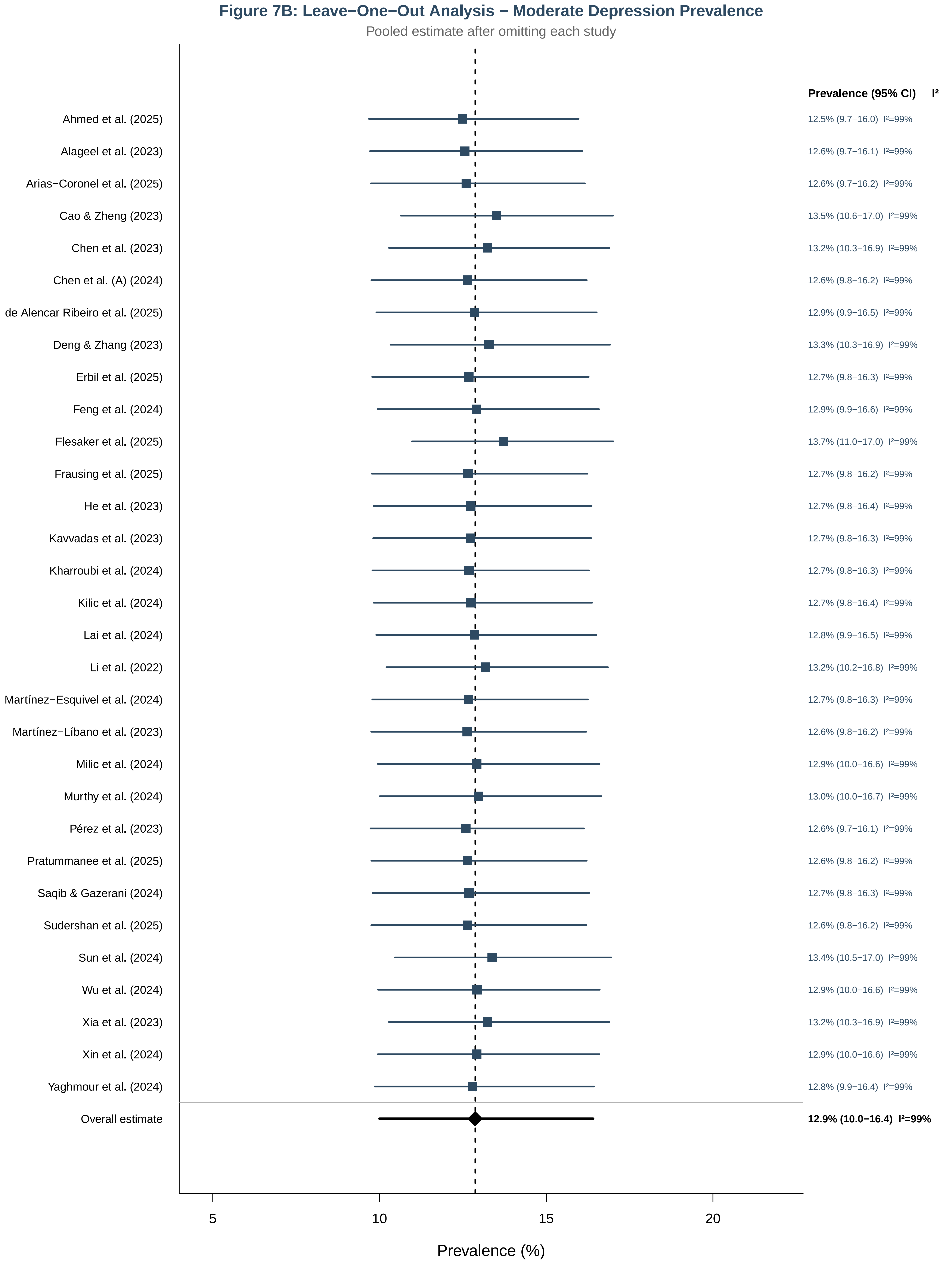
Figure 7B: Leave−One−Out Analysis − Moderate Depression Prevalence
Pooled estimate after omitting each study
Prevalence (95% CI) I²
Ahmed et al. (2025) 12.5% (9.7−16.0) I²=99%
Alageel et al. (2023) 12.6% (9.7−16.1) I²=99%
Arias−Coronel et al. (2025) 12.6% (9.7−16.2) I²=99%
Cao & Zheng (2023) 13.5% (10.6−17.0) I²=99%
Chen et al. (2023) 13.2% (10.3−16.9) I²=99%
Chen et al. (A) (2024) 12.6% (9.8−16.2) I²=99%
de Alencar Ribeiro et al. (2025) 12.9% (9.9−16.5) I²=99%
Deng & Zhang (2023) 13.3% (10.3−16.9) I²=99%
Erbil et al. (2025) 12.7% (9.8−16.3) I²=99%
Feng et al. (2024) 12.9% (9.9−16.6) I²=99%
Flesaker et al. (2025) 13.7% (11.0−17.0) I²=99%
Frausing et al. (2025) 12.7% (9.8−16.2) I²=99%
He et al. (2023) 12.7% (9.8−16.4) I²=99%
Kavvadas et al. (2023) 12.7% (9.8−16.3) I²=99%
Kharroubi et al. (2024) 12.7% (9.8−16.3) I²=99%
Kilic et al. (2024) 12.7% (9.8−16.4) I²=99%
Lai et al. (2024) 12.8% (9.9−16.5) I²=99%
Li et al. (2022) 13.2% (10.2−16.8) I²=99%
Martínez−Esquivel et al. (2024) 12.7% (9.8−16.3) I²=99%
Martínez−Líbano et al. (2023) 12.6% (9.8−16.2) I²=99%
Milic et al. (2024) 12.9% (10.0−16.6) I²=99%
Murthy et al. (2024) 13.0% (10.0−16.7) I²=99%
Pérez et al. (2023) 12.6% (9.7−16.1) I²=99%
Pratummanee et al. (2025) 12.6% (9.8−16.2) I²=99%
Saqib & Gazerani (2024) 12.7% (9.8−16.3) I²=99%
Sudershan et al. (2025) 12.6% (9.8−16.2) I²=99%
Sun et al. (2024) 13.4% (10.5−17.0) I²=99%
Wu et al. (2024) 12.9% (10.0−16.6) I²=99%
Xia et al. (2023) 13.2% (10.3−16.9) I²=99%
Xin et al. (2024) 12.9% (10.0−16.6) I²=99%
Yaghmour et al. (2024) 12.8% (9.9−16.4) I²=99%
Overall estimate 12.9% (10.0−16.4) I²=99%
5
10
15
20
Prevalence (%)

Figure 7C: Subgroup Analysis − Moderate Depression Prevalence

Stratified by study characteristics

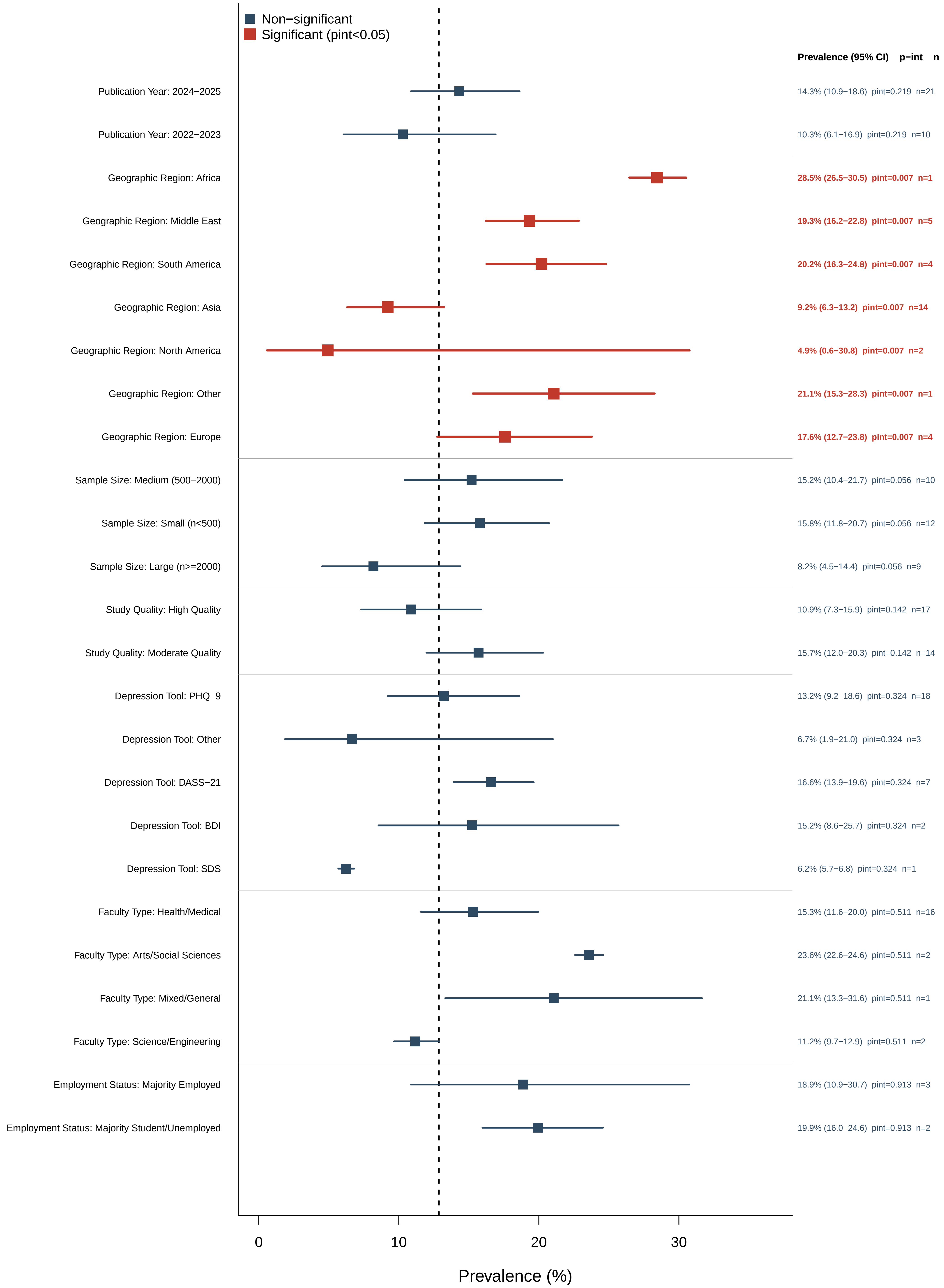

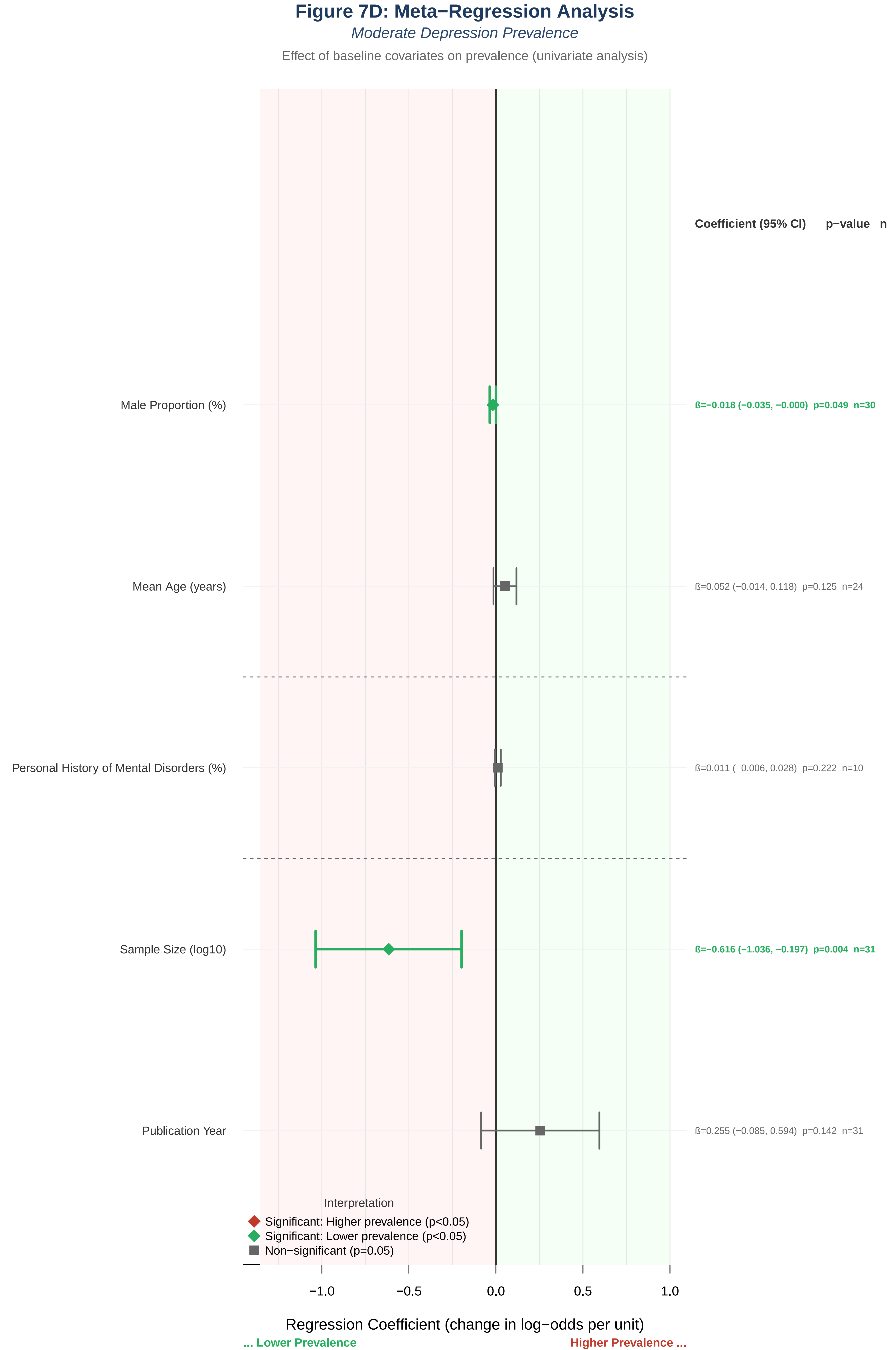

Figure 7D: Meta−Regression Analysis
Moderate Depression Prevalence
Effect of baseline covariates on prevalence (univariate analysis)
Coefficient (95% CI) p−value n
Male Proportion (%)
ß=−0.018 (−0.035, −0.000) p=0.049 n=30
Mean Age (years)
ß=0.052 (−0.014, 0.118) p=0.125 n=24
Personal History of Mental Disorders (%)
ß=0.011 (−0.006, 0.028) p=0.222 n=10
Sample Size (log10)
ß=−0.616 (−1.036, −0.197) p=0.004 n=31
Publication Year
ß=0.255 (−0.085, 0.594) p=0.142 n=31
Interpretation
Significant: Higher prevalence (p<0.05)
Significant: Lower prevalence (p<0.05)
Non−significant (p=0.05)
−1.0
−0.5
0.0
0.5
1.0
Regression Coefficient (change in log−odds per unit)
... Lower Prevalence
Higher Prevalence ...

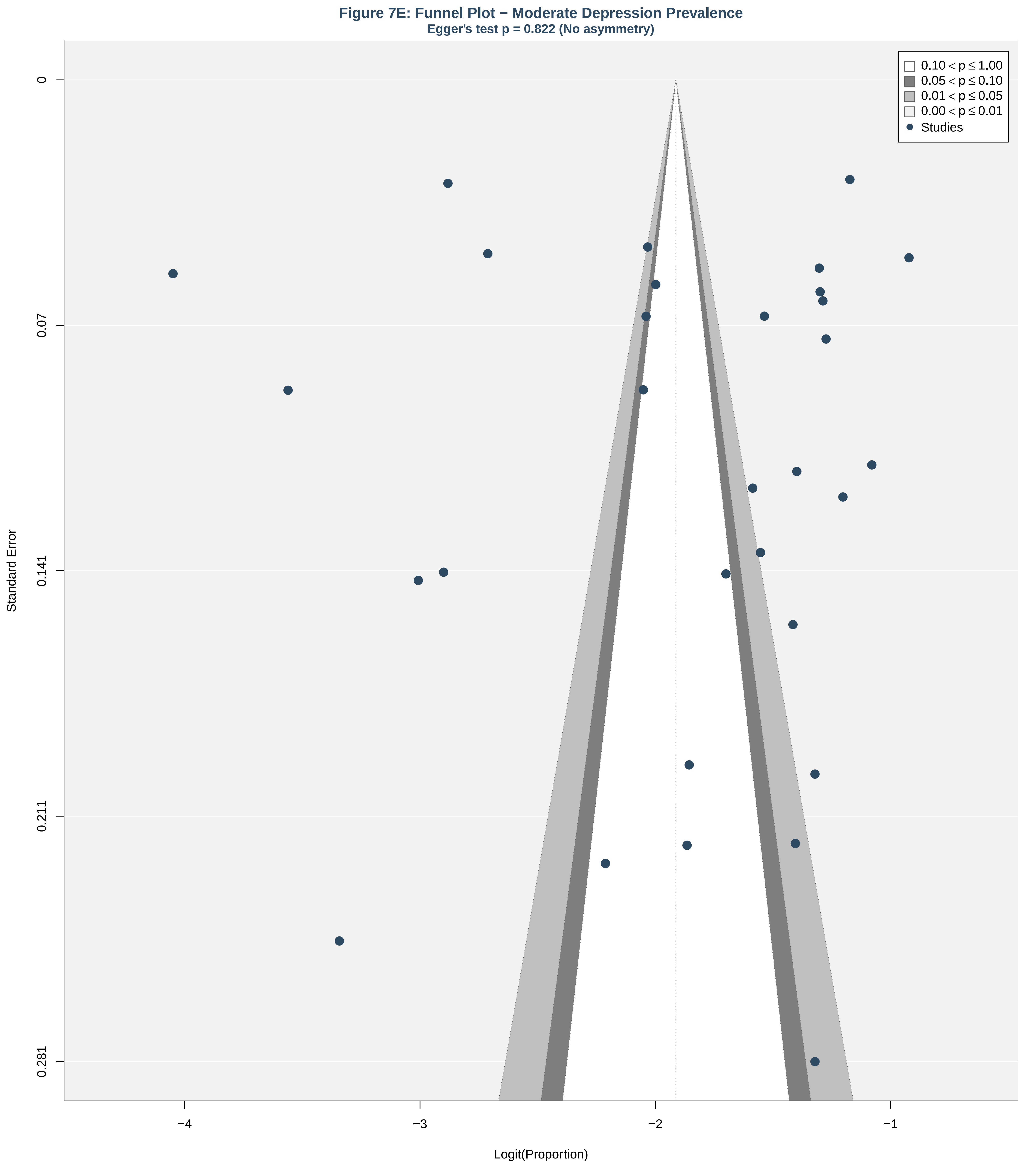

Figure 7E: Funnel Plot − Moderate Depression Prevalence
Egger's test p = 0.822 (No asymmetry)
0.10 < p ≤ 1.00
0.05 < p ≤ 0.10
0.01 < p ≤ 0.05
0.00 < p ≤ 0.01
Studies
Standard Error
0
0.07
0.141
0.211
0.281
−4
−3
−2
−1
Logit(Proportion)

Figure 8A: Forest Plot − Severe Depression Prevalence

N = 32 studies

Study	Events	N	Prevalence (%)	95% CI	Weight
Li et al. (2022)	7	6905	0.00	[0.00; 0.00]	−−%
Alageel et al. (2023)	26	434	0.06	[0.04; 0.09]	−−%
Cao & Zheng (2023)	62	4705	0.01	[0.01; 0.02]	−−%
Chen et al. (2023)	171	22624	0.01	[0.01; 0.01]	−−%
Deng & Zhang (2023)	18	1083	0.02	[0.01; 0.03]	−−%
He et al. (2023)	33	378	0.09	[0.06; 0.12]	−−%
Kavvadas et al. (2023)	249	1497	0.17	[0.15; 0.19]	−−%
Martínez−Líbano et al. (2023)	204	1062	0.19	[0.17; 0.22]	−−%
Pérez et al. (2023)	1686	6798	0.25	[0.24; 0.26]	−−%
Xia et al. (2023)	22	1016	0.02	[0.01; 0.03]	−−%
Chen et al. (A) (2024)	158	2046	0.08	[0.07; 0.09]	−−%
Kharroubi et al. (2024)	17	261	0.07	[0.04; 0.10]	−−%
Kilic et al. (2024)	0	518	0.00	[0.00; 0.01]	−−%
Lai et al. (2024)	13	222	0.06	[0.03; 0.10]	−−%
Li et al. (A) (2024)	3048	30475	0.10	[0.10; 0.10]	−−%
Makki et al. (2024)	265	520	0.51	[0.47; 0.55]	−−%
Martínez−Esquivel et al. (2024)	13	76	0.17	[0.09; 0.27]	−−%
Milic et al. (2024)	38	2137	0.02	[0.01; 0.02]	−−%
Murthy et al. (2024)	5	223	0.02	[0.01; 0.05]	−−%
Saqib & Gazerani (2024)	5	132	0.04	[0.01; 0.09]	−−%
Sun et al. (2024)	0	498	0.00	[0.00; 0.01]	−−%
Wu et al. (2024)	20	1255	0.02	[0.01; 0.02]	−−%
Xin et al. (2024)	478	4257	0.11	[0.10; 0.12]	−−%
Yaghmour et al. (2024)	0	382	0.00	[0.00; 0.01]	−−%
Ahmed et al. (2025)	165	1891	0.09	[0.07; 0.10]	−−%
Arias−Coronel et al. (2025)	48	394	0.12	[0.09; 0.16]	−−%
de Alencar Ribeiro et al. (2025)	14	179	0.08	[0.04; 0.13]	−−%
Erbil et al. (2025)	31	500	0.06	[0.04; 0.09]	−−%
Flesaker et al. (2025)	125	19265	0.01	[0.01; 0.01]	−−%
Frausing et al. (2025)	103	152	0.68	[0.60; 0.75]	−−%
Pratummanee et al. (2025)	74	1611	0.05	[0.04; 0.06]	−−%
Sudershan et al. (2025)	184	1471	0.13	[0.11; 0.14]	−−%
**Common effect model**		**114967**	**0.06**	**[0.06; 0.06]**	**.**
**Random effects model**			**0.04**	**[0.02; 0.08]**	**100.0%**

Heterogeneity: $I^2 = 99.4\%$, $\tau^2 = 3.3521$, $p = 0$

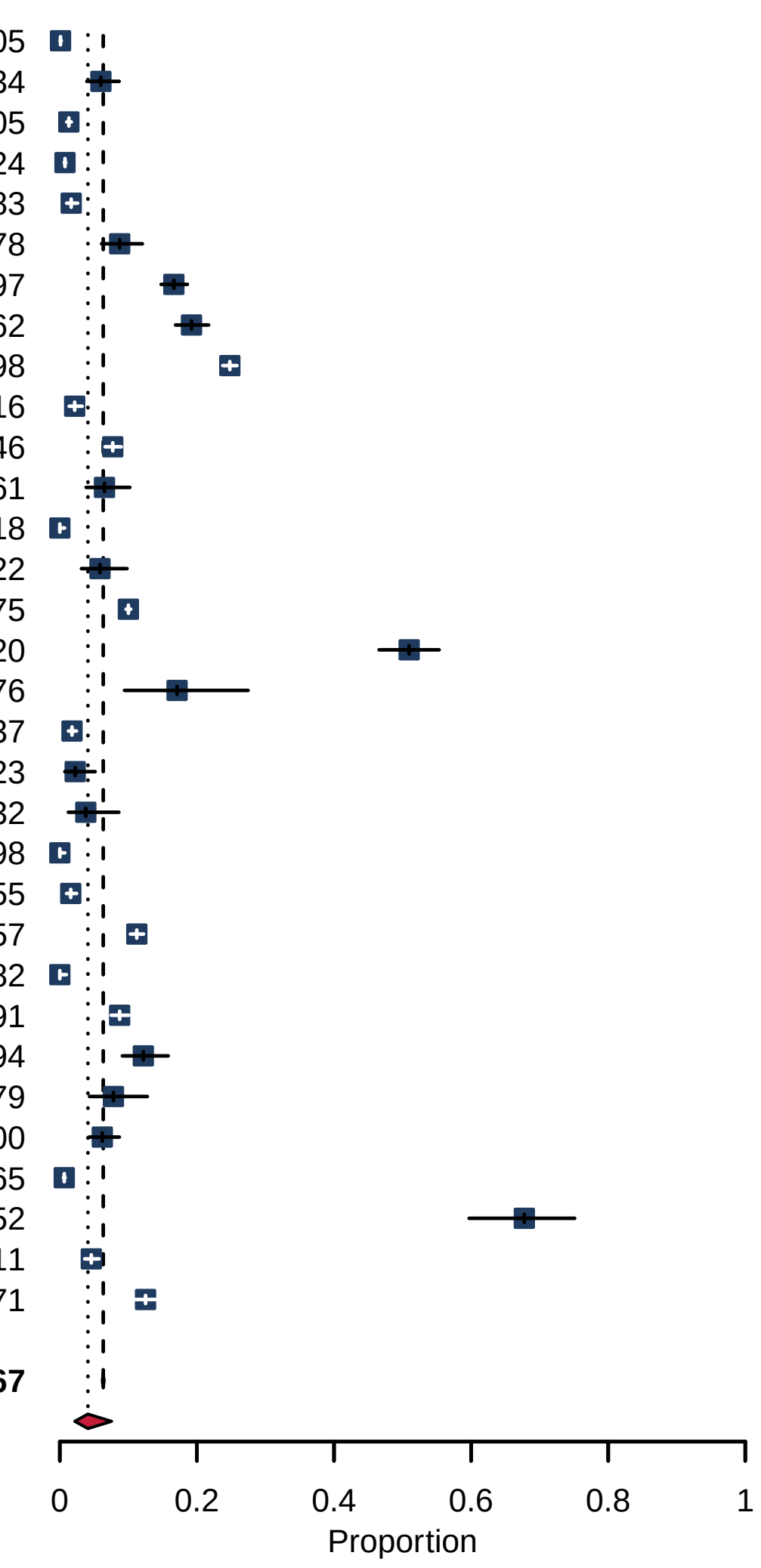

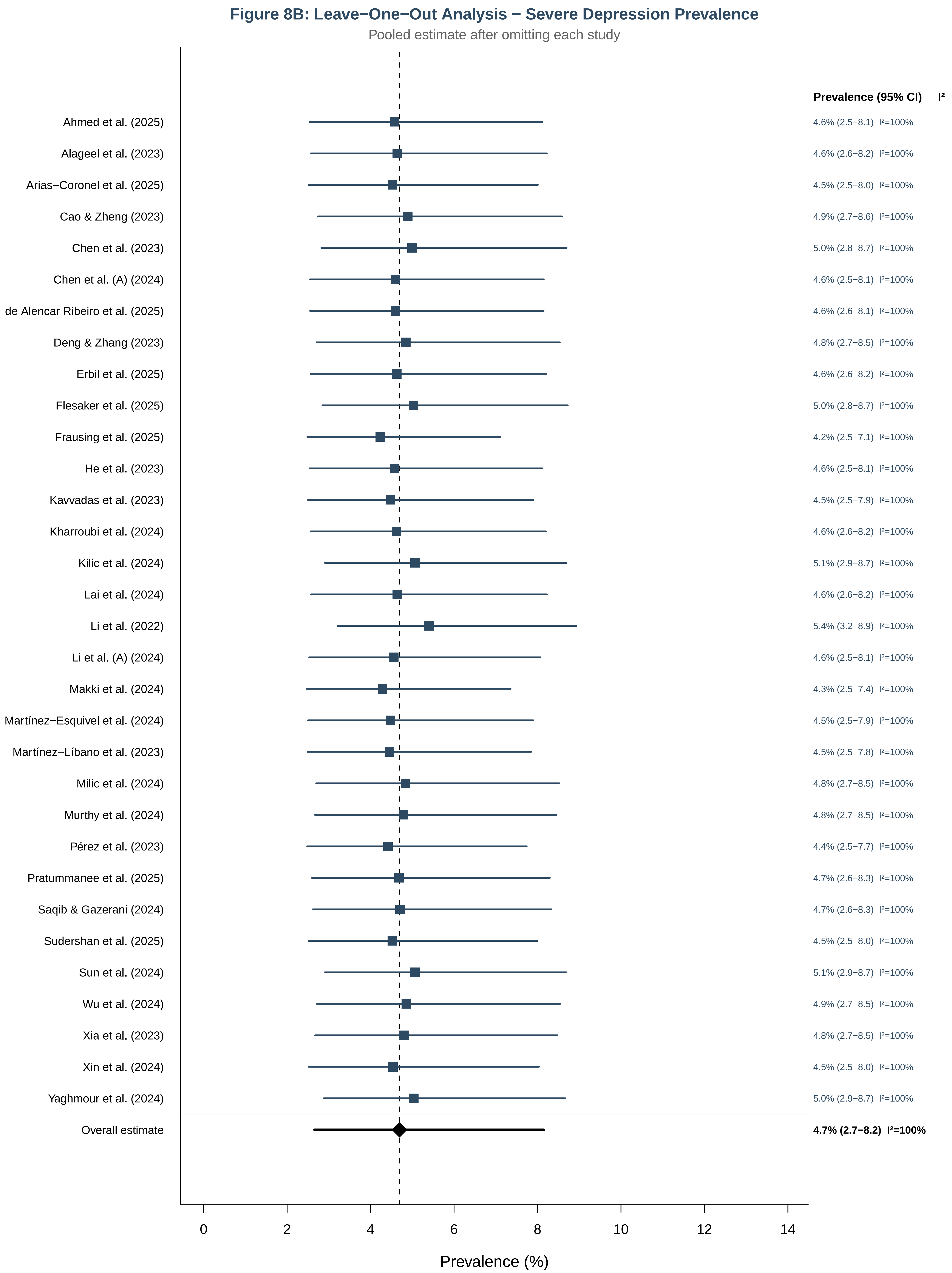

Figure 8B: Leave−One−Out Analysis − Severe Depression Prevalence
Pooled estimate after omitting each study
Prevalence (95% CI)
I²
Ahmed et al. (2025)
4.6% (2.5−8.1) I²=100%
Alageel et al. (2023)
4.6% (2.6−8.2) I²=100%
Arias−Coronel et al. (2025)
4.5% (2.5−8.0) I²=100%
Cao & Zheng (2023)
4.9% (2.7−8.6) I²=100%
Chen et al. (2023)
5.0% (2.8−8.7) I²=100%
Chen et al. (A) (2024)
4.6% (2.5−8.1) I²=100%
de Alencar Ribeiro et al. (2025)
4.6% (2.6−8.1) I²=100%
Deng & Zhang (2023)
4.8% (2.7−8.5) I²=100%
Erbil et al. (2025)
4.6% (2.6−8.2) I²=100%
Flesaker et al. (2025)
5.0% (2.8−8.7) I²=100%
Frausing et al. (2025)
4.2% (2.5−7.1) I²=100%
He et al. (2023)
4.6% (2.5−8.1) I²=100%
Kavvadas et al. (2023)
4.5% (2.5−7.9) I²=100%
Kharroubi et al. (2024)
4.6% (2.6−8.2) I²=100%
Kilic et al. (2024)
5.1% (2.9−8.7) I²=100%
Lai et al. (2024)
4.6% (2.6−8.2) I²=100%
Li et al. (2022)
5.4% (3.2−8.9) I²=100%
Li et al. (A) (2024)
4.6% (2.5−8.1) I²=100%
Makki et al. (2024)
4.3% (2.5−7.4) I²=100%
Martínez−Esquivel et al. (2024)
4.5% (2.5−7.9) I²=100%
Martínez−Líbano et al. (2023)
4.5% (2.5−7.8) I²=100%
Milic et al. (2024)
4.8% (2.7−8.5) I²=100%
Murthy et al. (2024)
4.8% (2.7−8.5) I²=100%
Pérez et al. (2023)
4.4% (2.5−7.7) I²=100%
Pratummanee et al. (2025)
4.7% (2.6−8.3) I²=100%
Saqib & Gazerani (2024)
4.7% (2.6−8.3) I²=100%
Sudershan et al. (2025)
4.5% (2.5−8.0) I²=100%
Sun et al. (2024)
5.1% (2.9−8.7) I²=100%
Wu et al. (2024)
4.9% (2.7−8.5) I²=100%
Xia et al. (2023)
4.8% (2.7−8.5) I²=100%
Xin et al. (2024)
4.5% (2.5−8.0) I²=100%
Yaghmour et al. (2024)
5.0% (2.9−8.7) I²=100%
Overall estimate
4.7% (2.7−8.2) I²=100%
0
2
4
6
8
10
12
14
Prevalence (%)

Figure 8C: Subgroup Analysis − Severe Depression Prevalence

Stratified by study characteristics

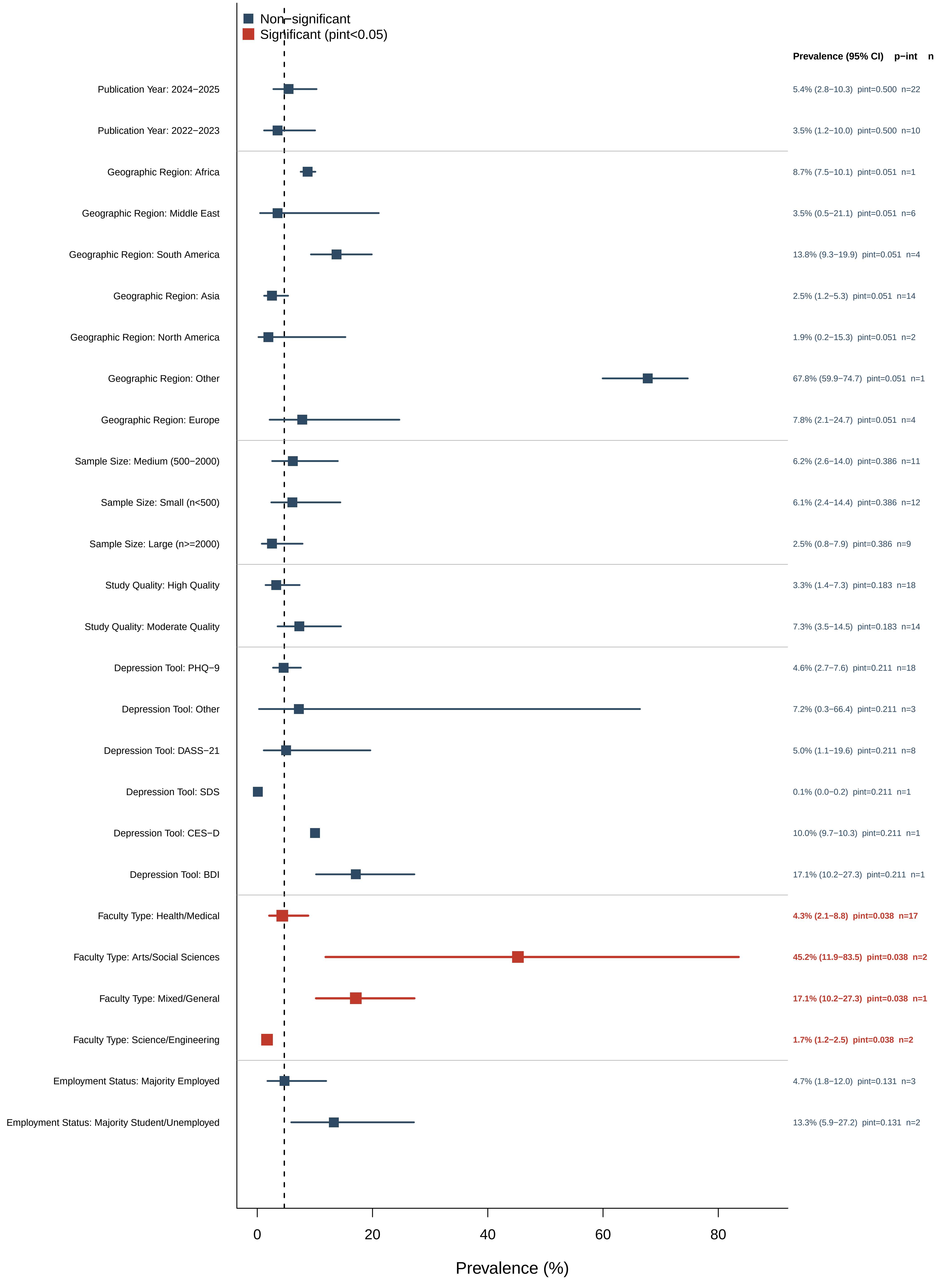

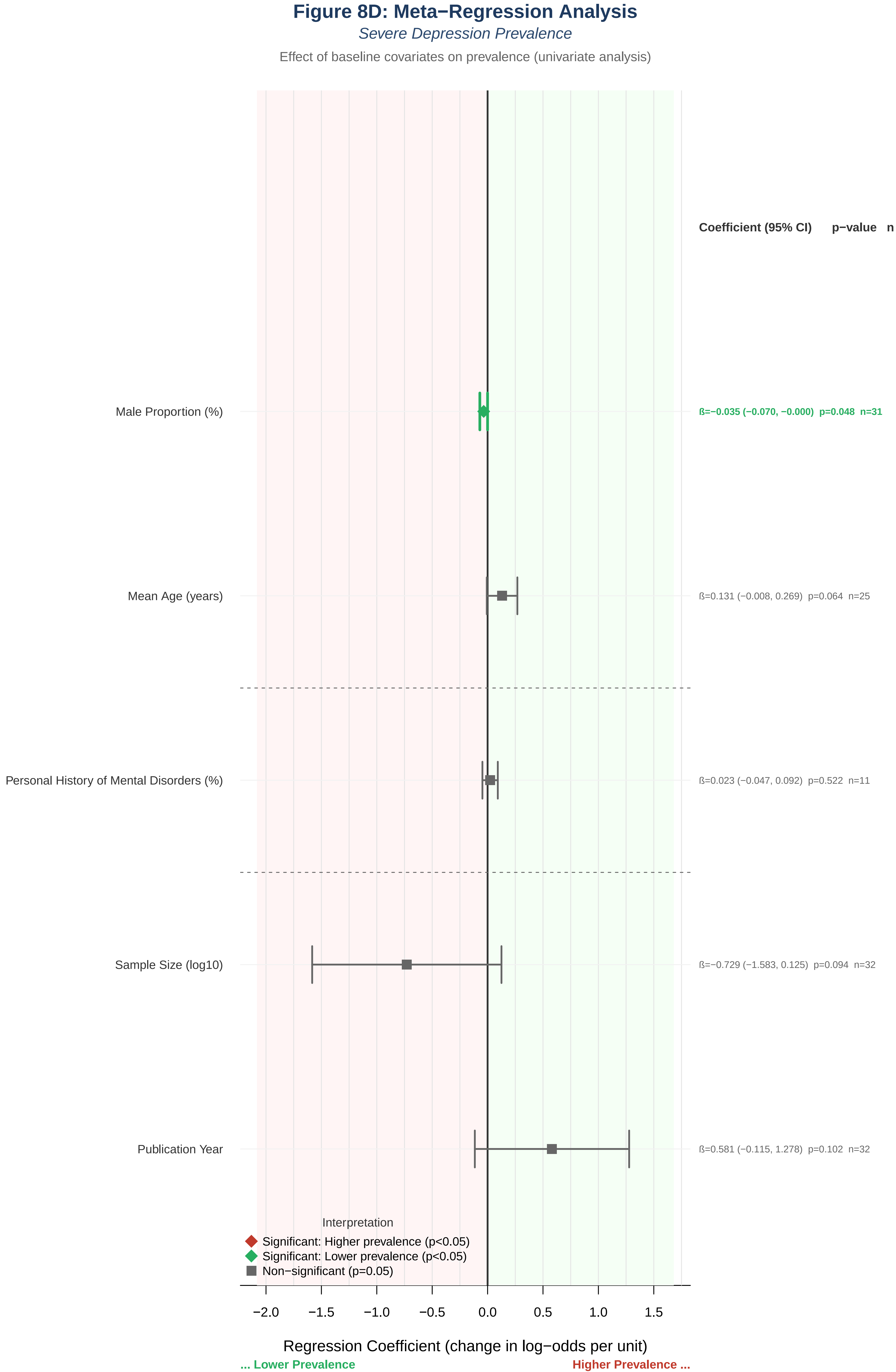
Figure 8D: Meta−Regression Analysis
Severe Depression Prevalence
Effect of baseline covariates on prevalence (univariate analysis)
Coefficient (95% CI) p−value n
Male Proportion (%)
ß=−0.035 (−0.070, −0.000) p=0.048 n=31
Mean Age (years)
ß=0.131 (−0.008, 0.269) p=0.064 n=25
Personal History of Mental Disorders (%)
ß=0.023 (−0.047, 0.092) p=0.522 n=11
Sample Size (log10)
ß=−0.729 (−1.583, 0.125) p=0.094 n=32
Publication Year
ß=0.581 (−0.115, 1.278) p=0.102 n=32
Interpretation
Significant: Higher prevalence (p<0.05)
Significant: Lower prevalence (p<0.05)
Non−significant (p=0.05)
−2.0 −1.5 −1.0 −0.5 0.0 0.5 1.0 1.5
Regression Coefficient (change in log−odds per unit)
... Lower Prevalence
Higher Prevalence ...

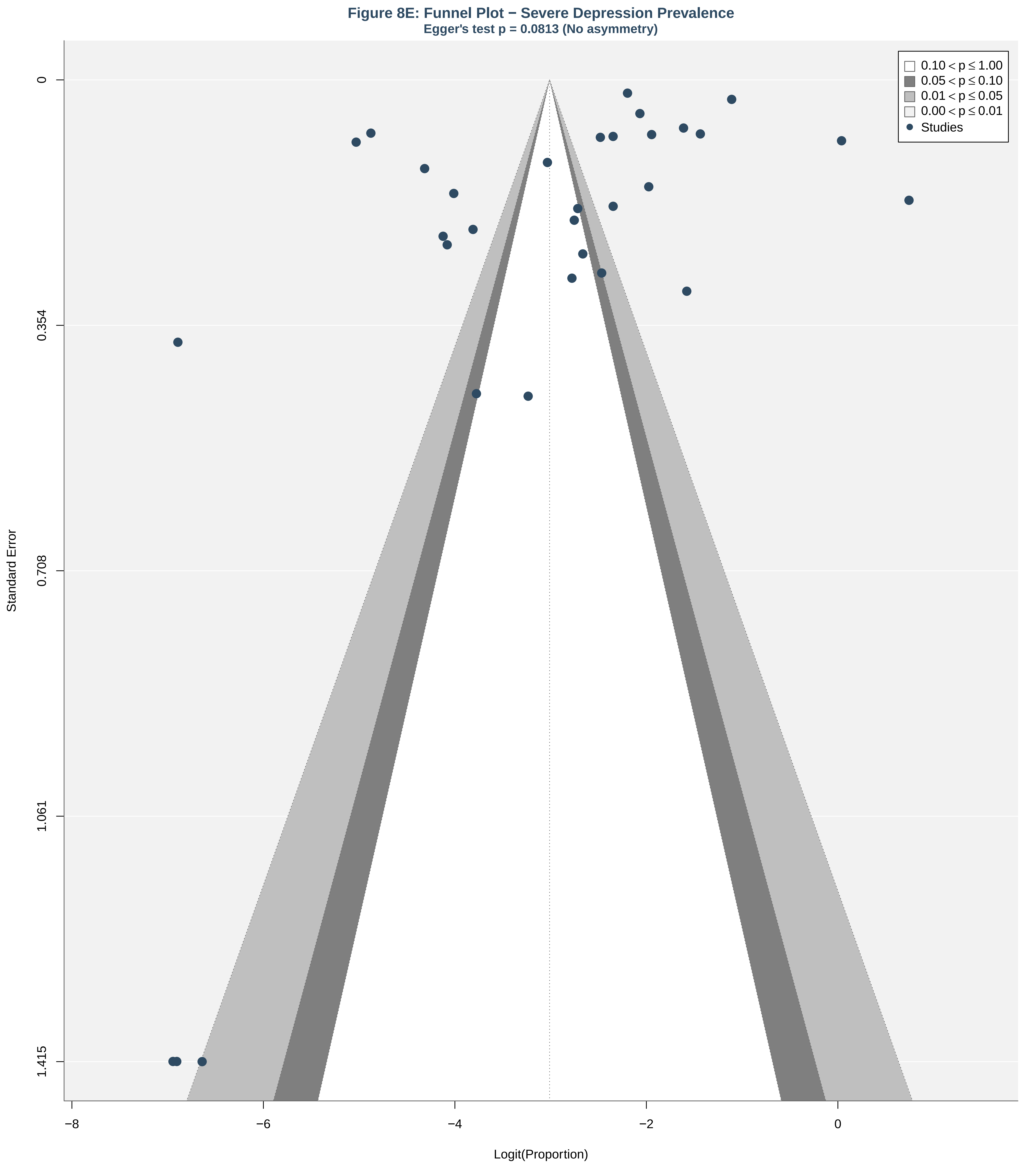
Figure 8E: Funnel Plot − Severe Depression Prevalence
Egger's test p = 0.0813 (No asymmetry)
0.10 < p ≤ 1.00
0.05 < p ≤ 0.10
0.01 < p ≤ 0.05
0.00 < p ≤ 0.01
Studies
Standard Error
0
0.354
0.708
1.061
1.415
−8
−6
−4
−2
0
Logit(Proportion)

Figure 9A: Forest Plot − Mild Anxiety Prevalence

N = 33 studies

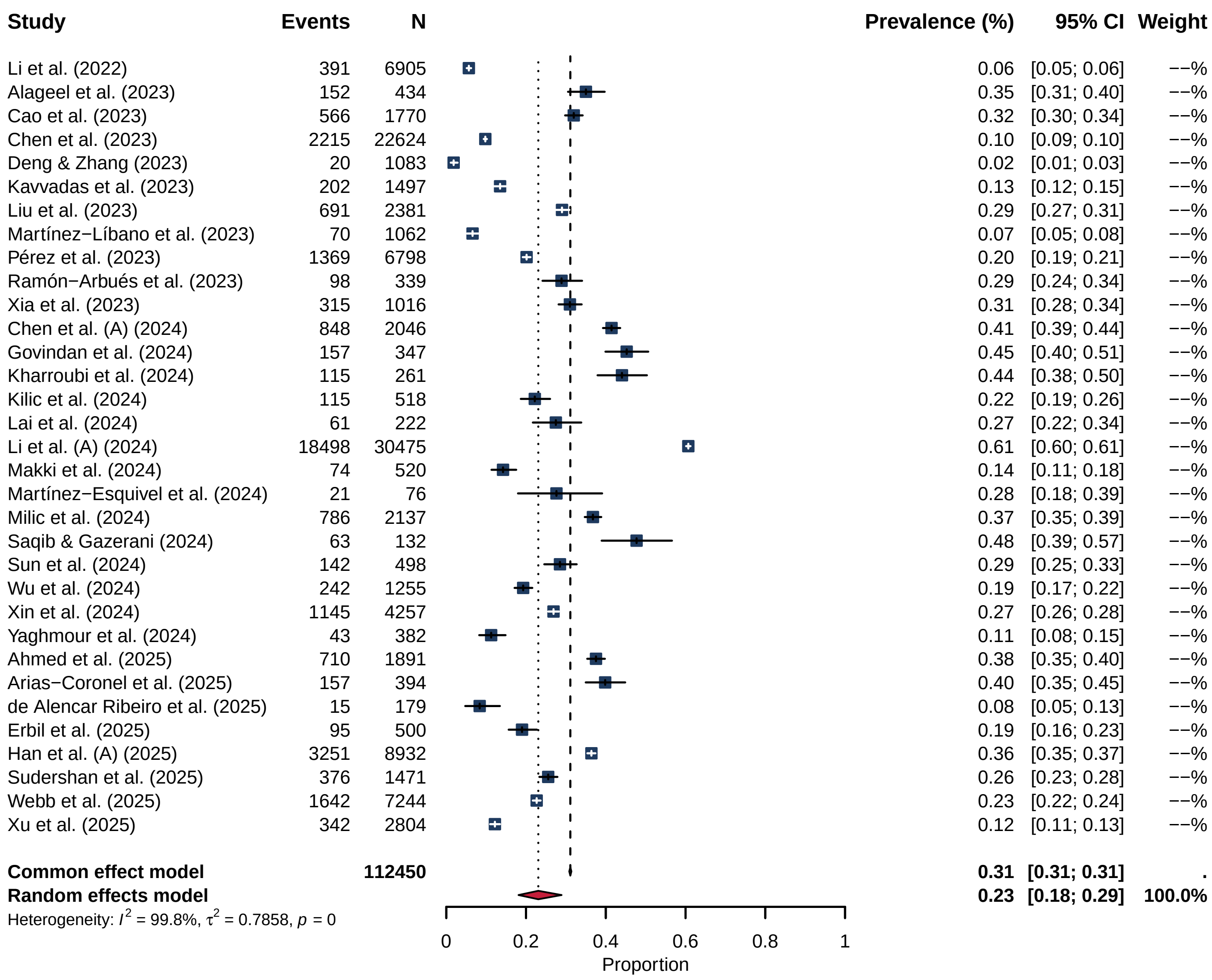

Figure 9B: Leave−One−Out Analysis − Mild Anxiety Prevalence

Pooled estimate after omitting each study

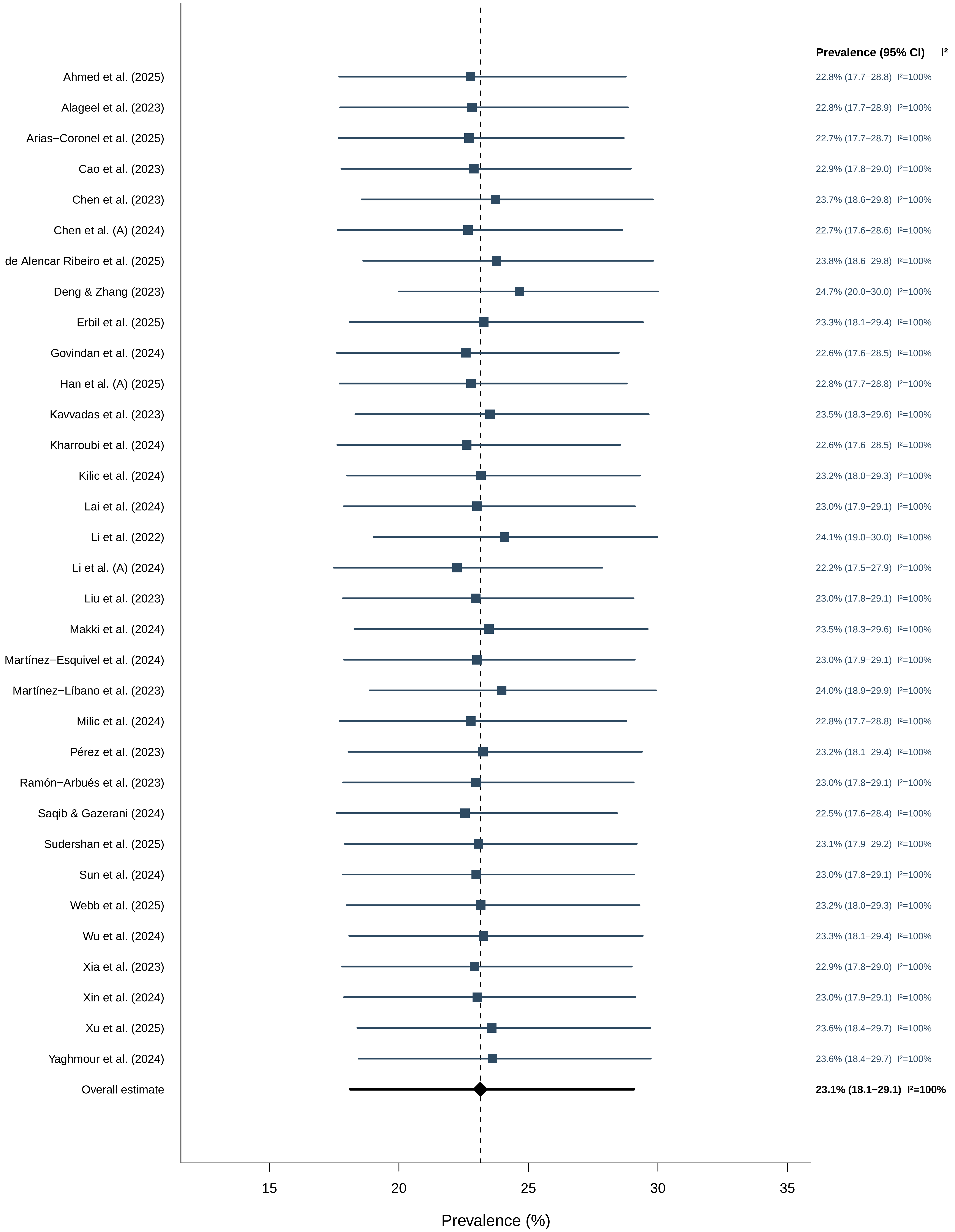

Figure 9C: Subgroup Analysis − Mild Anxiety Prevalence

Stratified by study characteristics

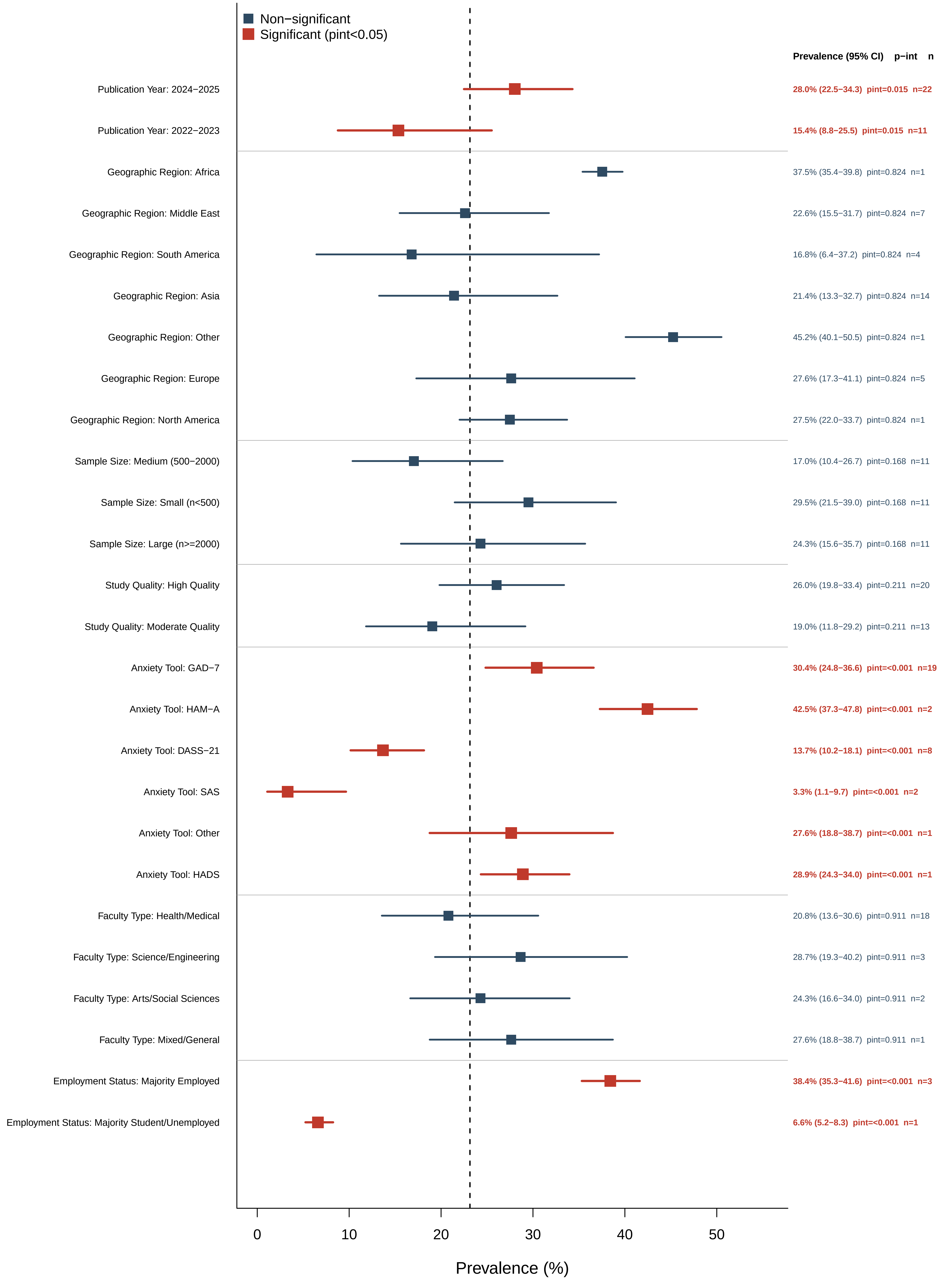

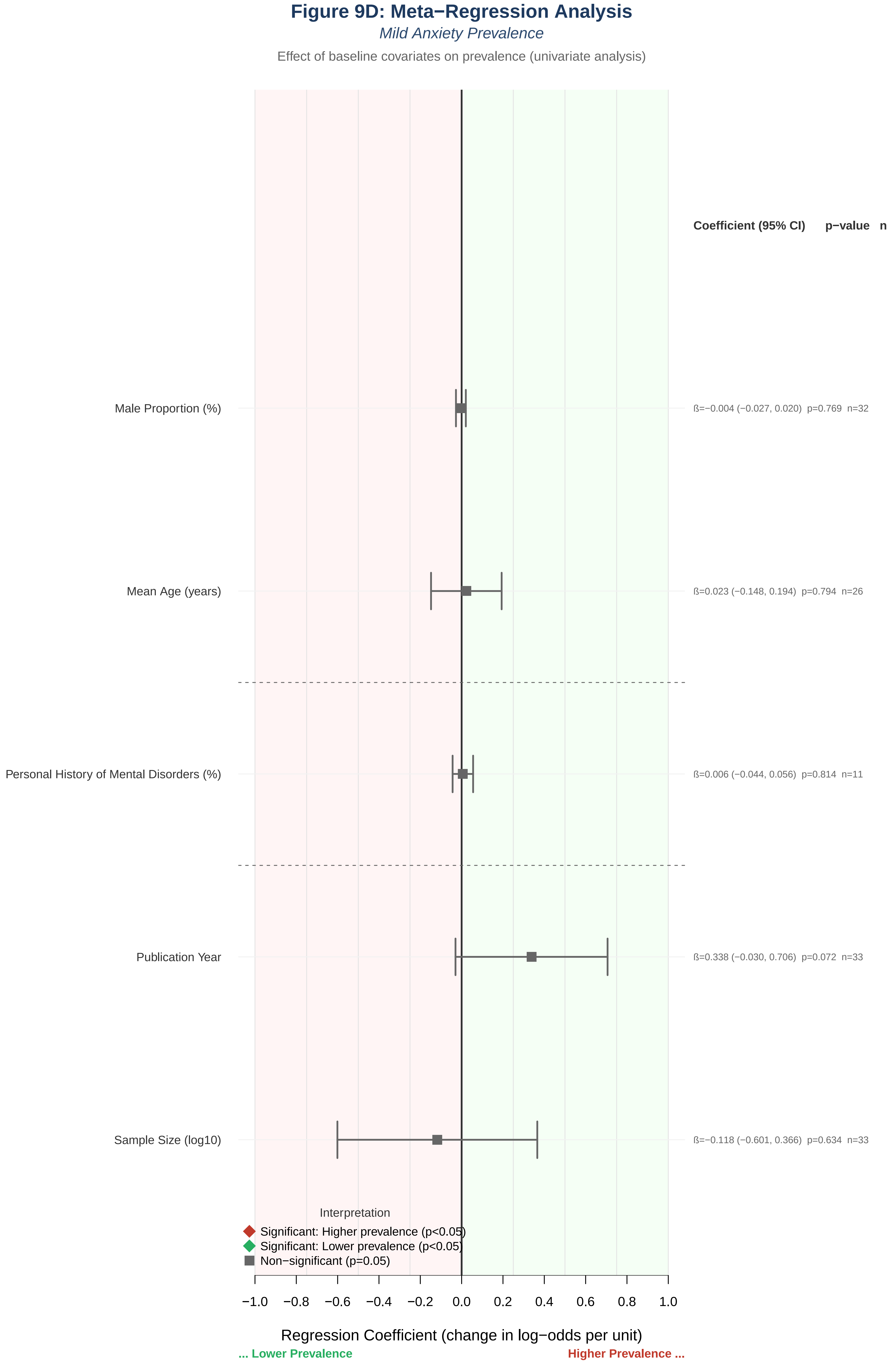
Figure 9D: Meta−Regression Analysis
Mild Anxiety Prevalence
Effect of baseline covariates on prevalence (univariate analysis)
Coefficient (95% CI) p−value n
Male Proportion (%)
ß=−0.004 (−0.027, 0.020) p=0.769 n=32
Mean Age (years)
ß=0.023 (−0.148, 0.194) p=0.794 n=26
Personal History of Mental Disorders (%)
ß=0.006 (−0.044, 0.056) p=0.814 n=11
Publication Year
ß=0.338 (−0.030, 0.706) p=0.072 n=33
Sample Size (log10)
ß=−0.118 (−0.601, 0.366) p=0.634 n=33
Interpretation
Significant: Higher prevalence (p<0.05)
Significant: Lower prevalence (p<0.05)
Non−significant (p=0.05)
−1.0
−0.8
−0.6
−0.4
−0.2
0.0
0.2
0.4
0.6
0.8
1.0
Regression Coefficient (change in log−odds per unit)
... Lower Prevalence
Higher Prevalence ...

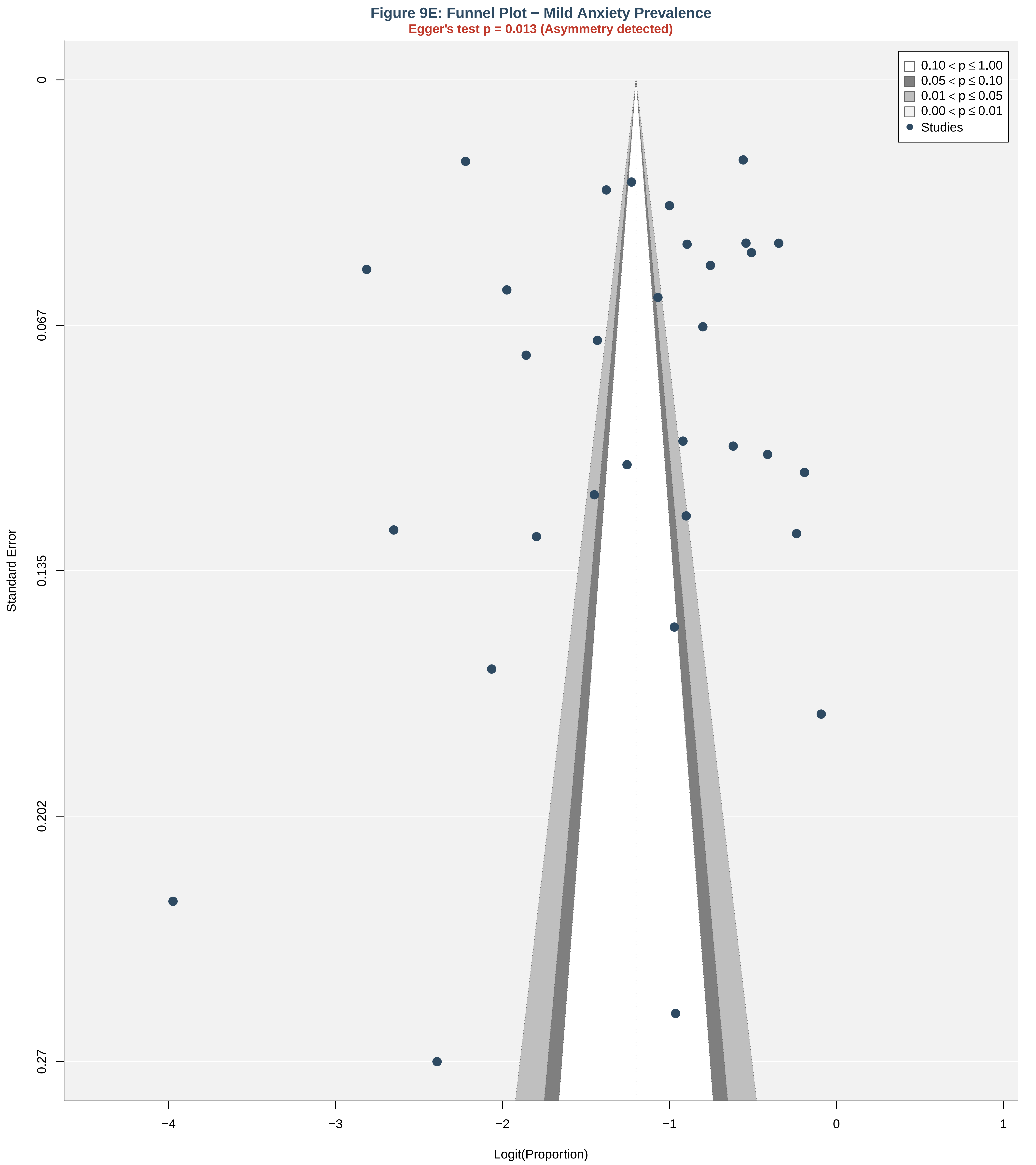

Figure 9E: Funnel Plot − Mild Anxiety Prevalence
Egger's test p = 0.013 (Asymmetry detected)
0.10 < p ≤ 1.00
0.05 < p ≤ 0.10
0.01 < p ≤ 0.05
0.00 < p ≤ 0.01
Studies
Standard Error
0
0.067
0.135
0.202
0.27
−4
−3
−2
−1
0
1
Logit(Proportion)

Figure 10A: Forest Plot − Moderate Anxiety Prevalence

N = 32 studies

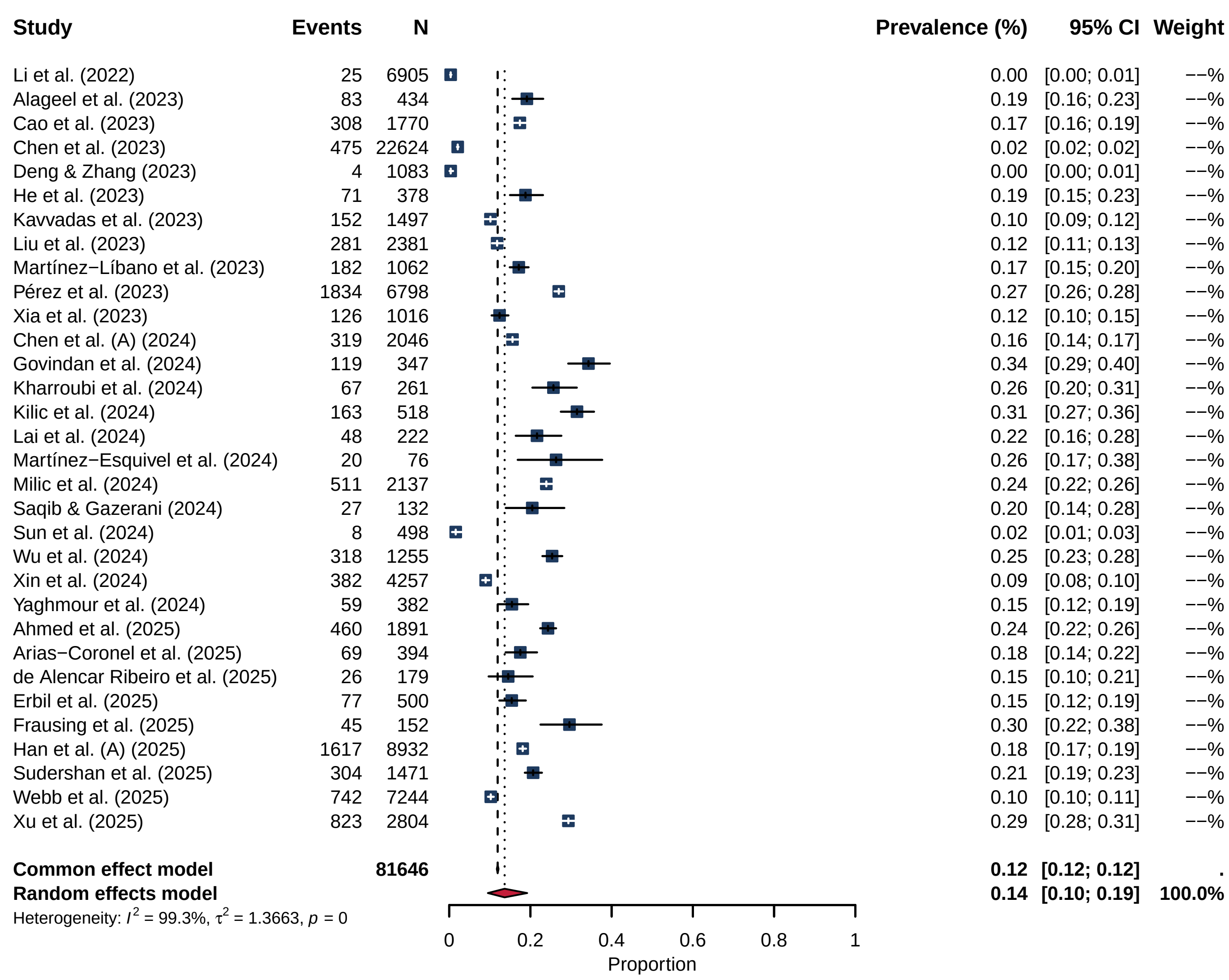

Figure 10B: Leave−One−Out Analysis − Moderate Anxiety Prevalence

Pooled estimate after omitting each study

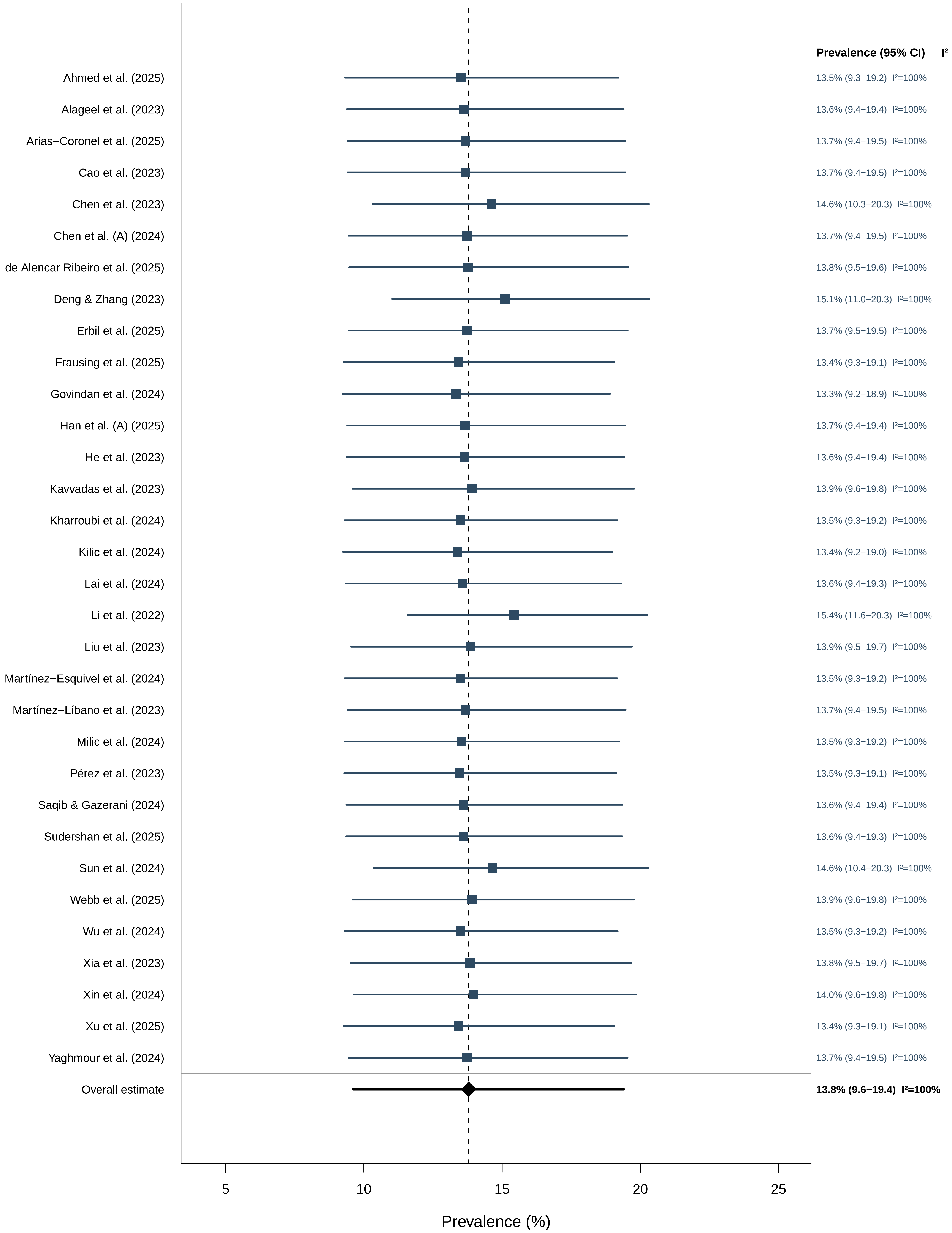

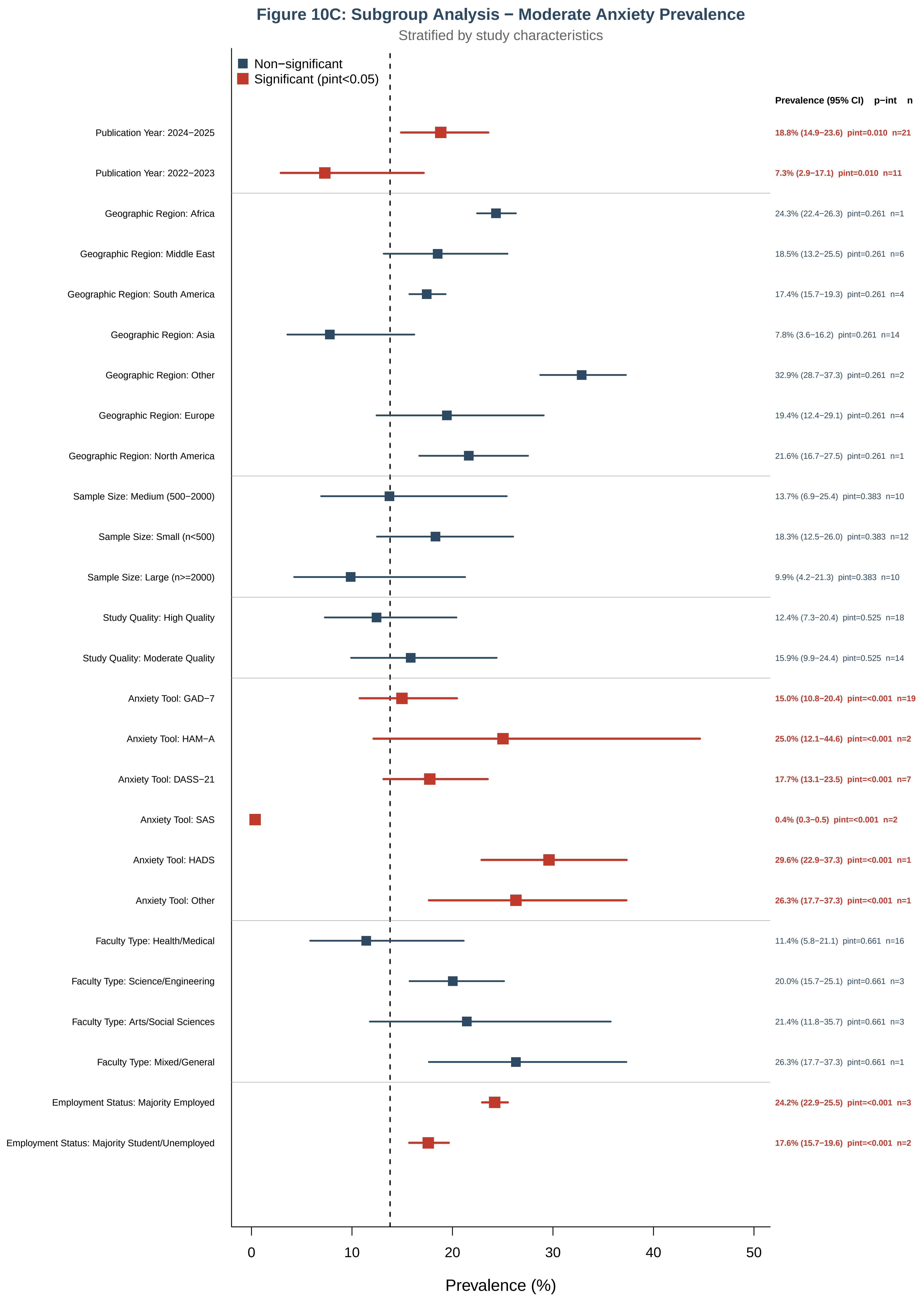

Figure 10C: Subgroup Analysis − Moderate Anxiety Prevalence
Stratified by study characteristics
Non−significant
Significant (pint<0.05)
Prevalence (95% CI) p−int n
Publication Year: 2024−2025
18.8% (14.9−23.6) pint=0.010 n=21
Publication Year: 2022−2023
7.3% (2.9−17.1) pint=0.010 n=11
Geographic Region: Africa
24.3% (22.4−26.3) pint=0.261 n=1
Geographic Region: Middle East
18.5% (13.2−25.5) pint=0.261 n=6
Geographic Region: South America
17.4% (15.7−19.3) pint=0.261 n=4
Geographic Region: Asia
7.8% (3.6−16.2) pint=0.261 n=14
Geographic Region: Other
32.9% (28.7−37.3) pint=0.261 n=2
Geographic Region: Europe
19.4% (12.4−29.1) pint=0.261 n=4
Geographic Region: North America
21.6% (16.7−27.5) pint=0.261 n=1
Sample Size: Medium (500−2000)
13.7% (6.9−25.4) pint=0.383 n=10
Sample Size: Small (n<500)
18.3% (12.5−26.0) pint=0.383 n=12
Sample Size: Large (n>=2000)
9.9% (4.2−21.3) pint=0.383 n=10
Study Quality: High Quality
12.4% (7.3−20.4) pint=0.525 n=18
Study Quality: Moderate Quality
15.9% (9.9−24.4) pint=0.525 n=14
Anxiety Tool: GAD−7
15.0% (10.8−20.4) pint=<0.001 n=19
Anxiety Tool: HAM−A
25.0% (12.1−44.6) pint=<0.001 n=2
Anxiety Tool: DASS−21
17.7% (13.1−23.5) pint=<0.001 n=7
Anxiety Tool: SAS
0.4% (0.3−0.5) pint=<0.001 n=2
Anxiety Tool: HADS
29.6% (22.9−37.3) pint=<0.001 n=1
Anxiety Tool: Other
26.3% (17.7−37.3) pint=<0.001 n=1
Faculty Type: Health/Medical
11.4% (5.8−21.1) pint=0.661 n=16
Faculty Type: Science/Engineering
20.0% (15.7−25.1) pint=0.661 n=3
Faculty Type: Arts/Social Sciences
21.4% (11.8−35.7) pint=0.661 n=3
Faculty Type: Mixed/General
26.3% (17.7−37.3) pint=0.661 n=1
Employment Status: Majority Employed
24.2% (22.9−25.5) pint=<0.001 n=3
Employment Status: Majority Student/Unemployed
17.6% (15.7−19.6) pint=<0.001 n=2
0
10
20
30
40
50
Prevalence (%)

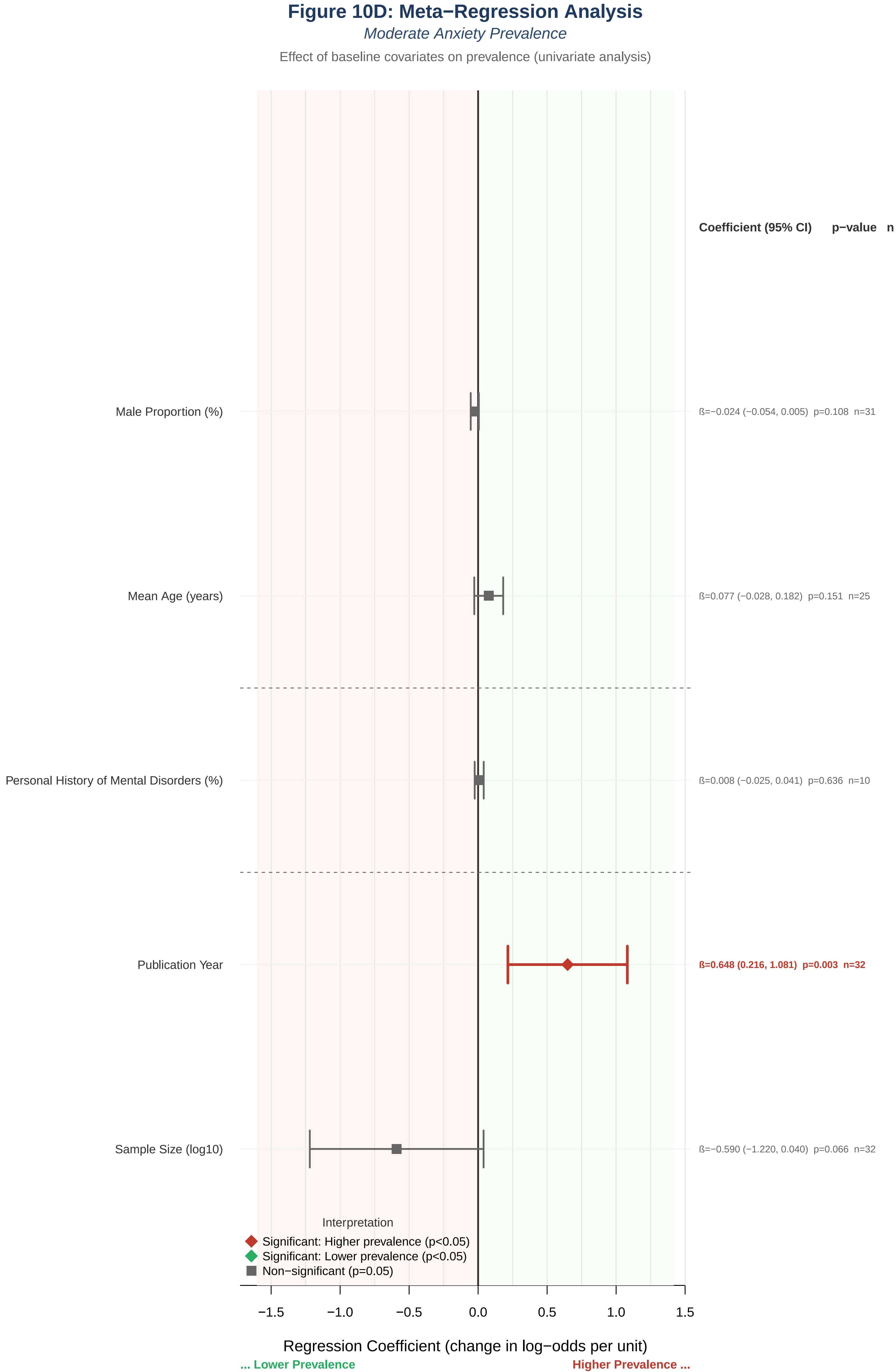
Figure 10D: Meta−Regression Analysis
Moderate Anxiety Prevalence
Effect of baseline covariates on prevalence (univariate analysis)
Coefficient (95% CI) p−value n
Male Proportion (%)
ß=−0.024 (−0.054, 0.005) p=0.108 n=31
Mean Age (years)
ß=0.077 (−0.028, 0.182) p=0.151 n=25
Personal History of Mental Disorders (%)
ß=0.008 (−0.025, 0.041) p=0.636 n=10
Publication Year
ß=0.648 (0.216, 1.081) p=0.003 n=32
Sample Size (log10)
ß=−0.590 (−1.220, 0.040) p=0.066 n=32
Interpretation
Significant: Higher prevalence (p<0.05)
Significant: Lower prevalence (p<0.05)
Non−significant (p=0.05)
−1.5
−1.0
−0.5
0.0
0.5
1.0
1.5
Regression Coefficient (change in log−odds per unit)
... Lower Prevalence
Higher Prevalence ...

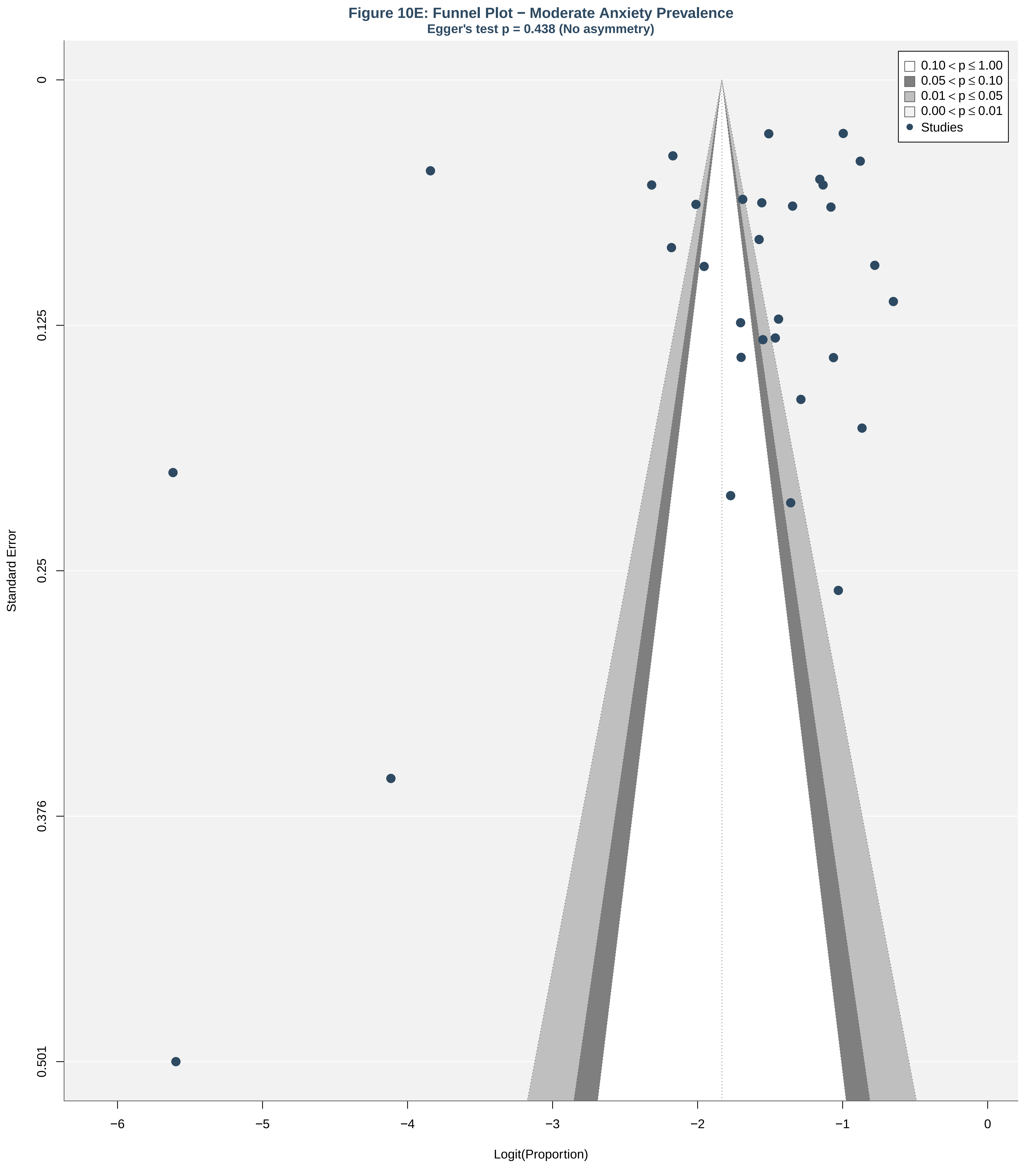
Figure 10E: Funnel Plot − Moderate Anxiety Prevalence
Egger's test p = 0.438 (No asymmetry)
0.10 < p ≤ 1.00
0.05 < p ≤ 0.10
0.01 < p ≤ 0.05
0.00 < p ≤ 0.01
Studies
Standard Error
0
0.125
0.25
0.376
0.501
−6
−5
−4
−3
−2
−1
0
Logit(Proportion)

Figure 11A: Forest Plot − Severe Anxiety Prevalence

N = 33 studies

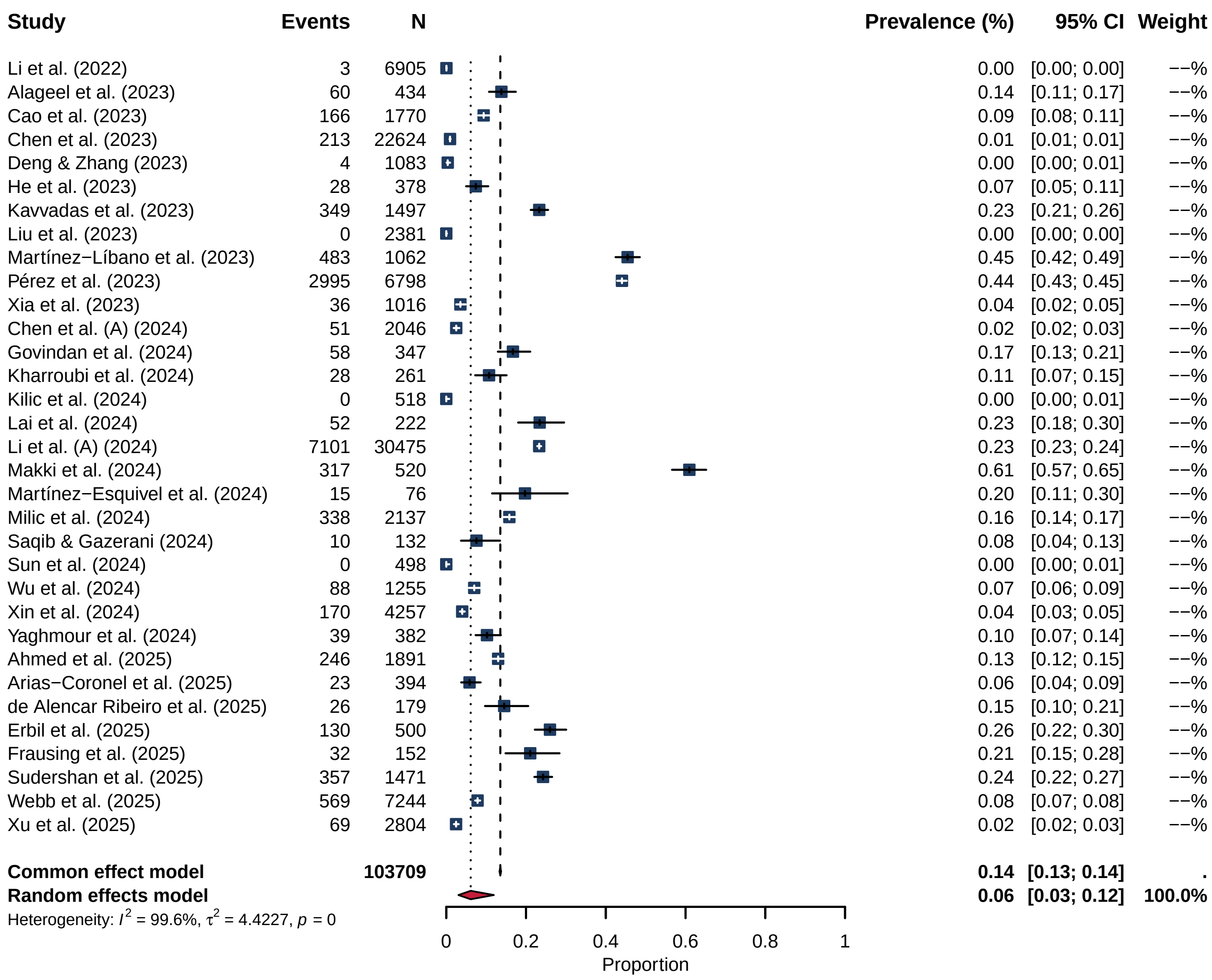

Figure 11B: Leave−One−Out Analysis − Severe Anxiety Prevalence

Pooled estimate after omitting each study

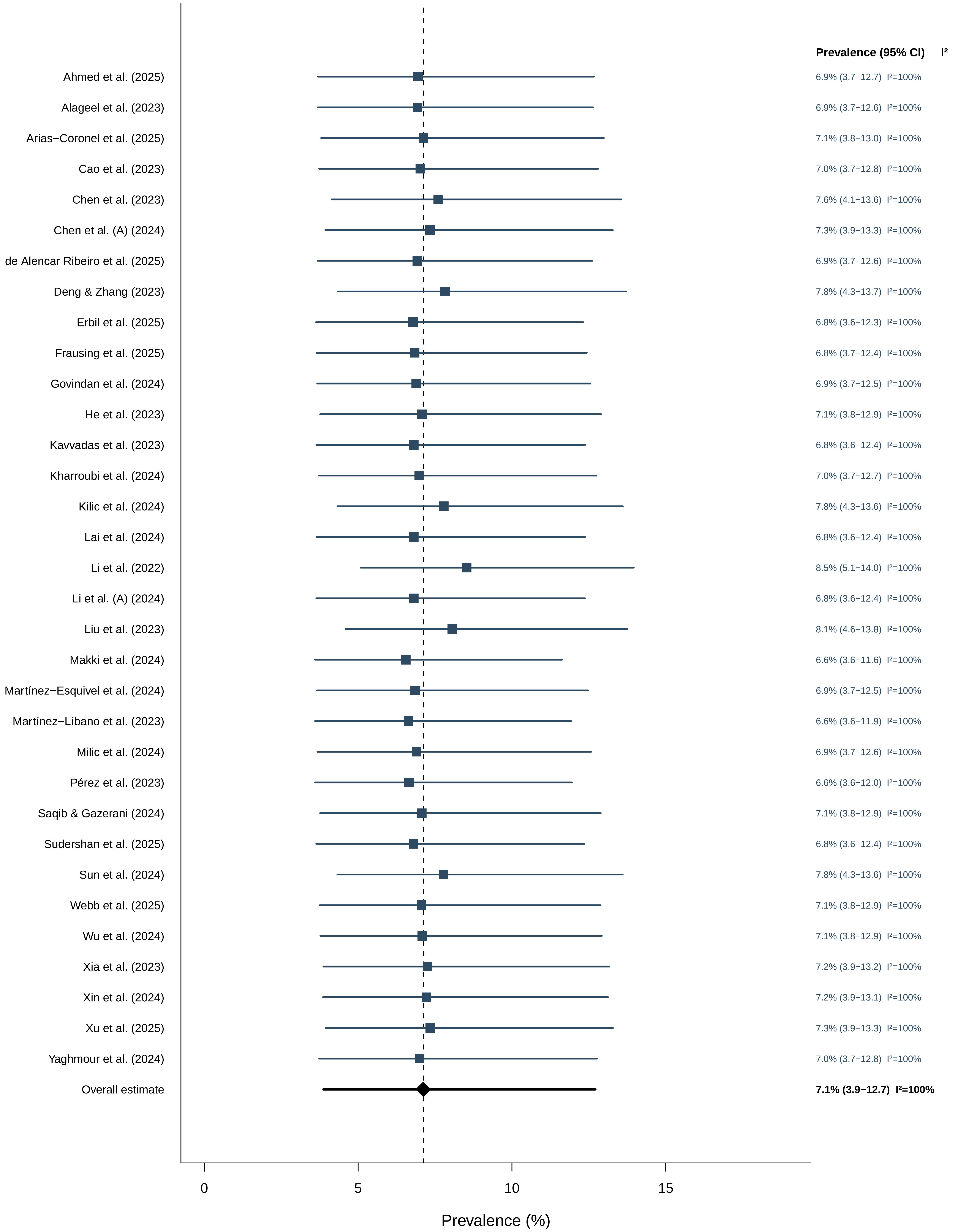

Figure 11C: Subgroup Analysis − Severe Anxiety Prevalence

Stratified by study characteristics

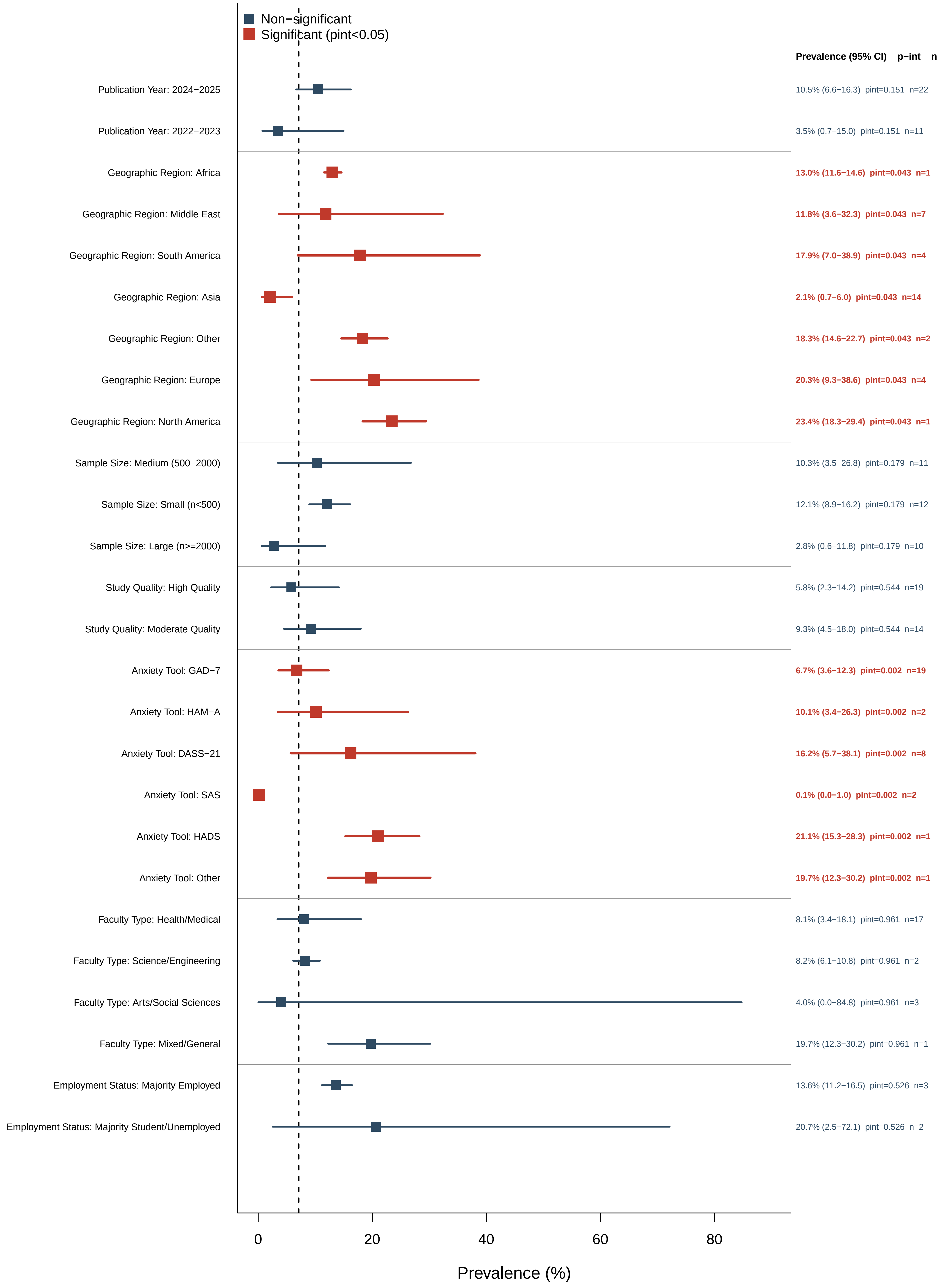

Figure 11D: Meta−Regression Analysis

Severe Anxiety Prevalence

Effect of baseline covariates on prevalence (univariate analysis)

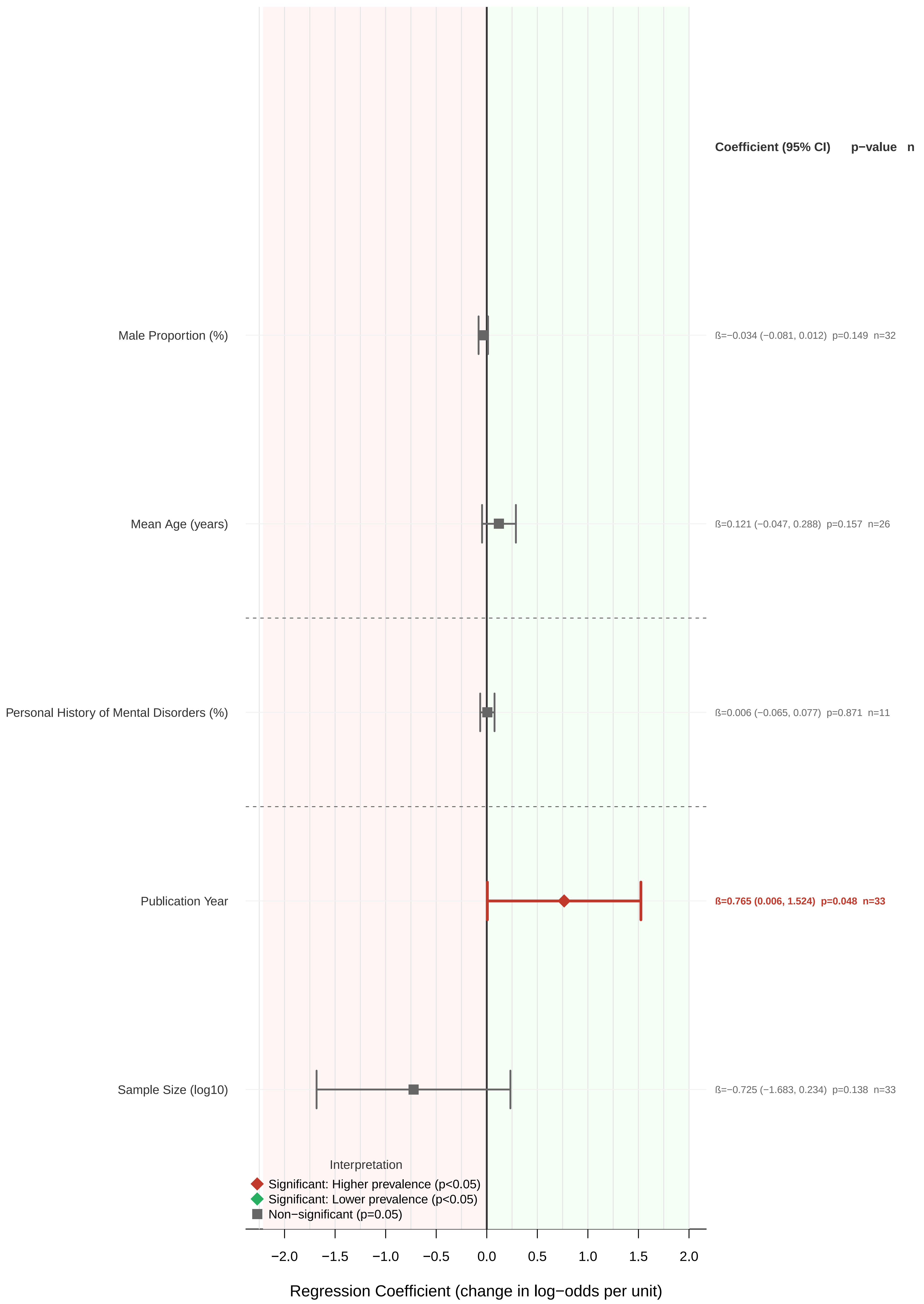

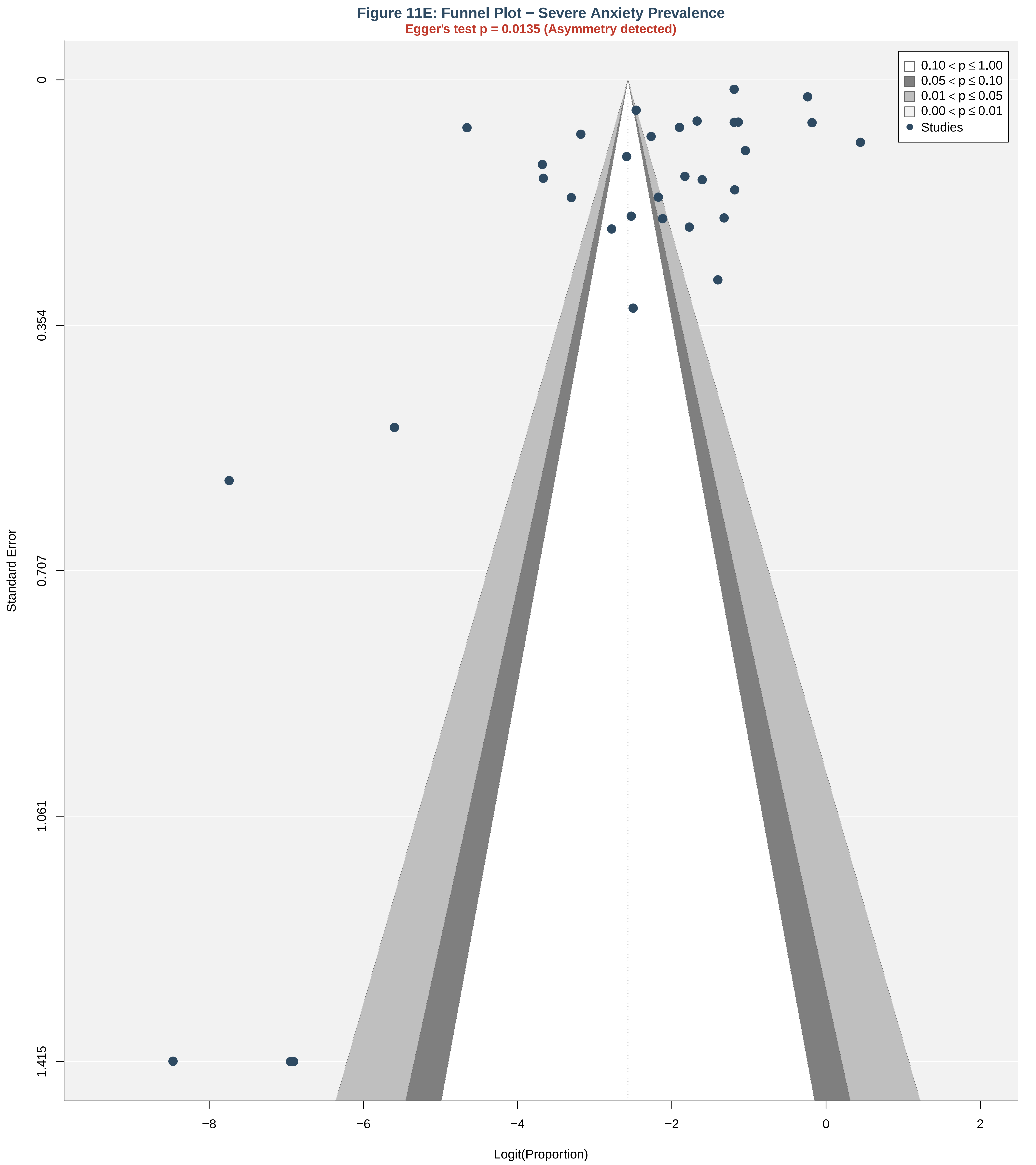
Figure 11E: Funnel Plot − Severe Anxiety Prevalence
Egger's test p = 0.0135 (Asymmetry detected)
0.10 < p ≤ 1.00
0.05 < p ≤ 0.10
0.01 < p ≤ 0.05
0.00 < p ≤ 0.01
Studies
Standard Error
0
0.354
0.707
1.061
1.415
−8
−6
−4
−2
0
2
Logit(Proportion)

Figure 12A: Forest Plot − High Perceived Stress Prevalence

N = 19 studies

Study	Events	N	Prevalence (%)	95% CI	Weight
Cao et al. (2023)	639	1770	0.36	[0.34; 0.38]	−−%
Chen et al. (2023)	598	22624	0.03	[0.02; 0.03]	−−%
Kavvadas et al. (2023)	318	1497	0.21	[0.19; 0.23]	−−%
Liu et al. (2023)	1327	2381	0.56	[0.54; 0.58]	−−%
Martínez−Líbano et al. (2023)	301	1062	0.28	[0.26; 0.31]	−−%
Chen et al. (A) (2024)	172	2046	0.08	[0.07; 0.10]	−−%
Kharroubi et al. (2024)	35	261	0.13	[0.10; 0.18]	−−%
Kilic et al. (2024)	0	518	0.00	[0.00; 0.01]	−−%
Lai et al. (2024)	29	222	0.13	[0.09; 0.18]	−−%
Li et al. (B) (2024)	2133	7845	0.27	[0.26; 0.28]	−−%
Makki et al. (2024)	238	520	0.46	[0.41; 0.50]	−−%
Simionescu et al. (2024)	236	422	0.56	[0.51; 0.61]	−−%
Wu et al. (2024)	103	1255	0.08	[0.07; 0.10]	−−%
Yaghmour et al. (2024)	0	382	0.00	[0.00; 0.01]	−−%
Ahmed et al. (2025)	395	1891	0.21	[0.19; 0.23]	−−%
de Alencar Ribeiro et al. (2025)	58	179	0.32	[0.26; 0.40]	−−%
Erbil et al. (2025)	53	500	0.11	[0.08; 0.14]	−−%
Frausing et al. (2025)	70	152	0.46	[0.38; 0.54]	−−%
Sudershan et al. (2025)	282	1471	0.19	[0.17; 0.21]	−−%
**Common effect model**		**46998**	**0.15**	**[0.15; 0.15]**	**.**
**Random effects model**			**0.14**	**[0.07; 0.28]**	**100.0%**

Heterogeneity: I^2 = 99.7%, τ^2 = 3.2100, p = 0

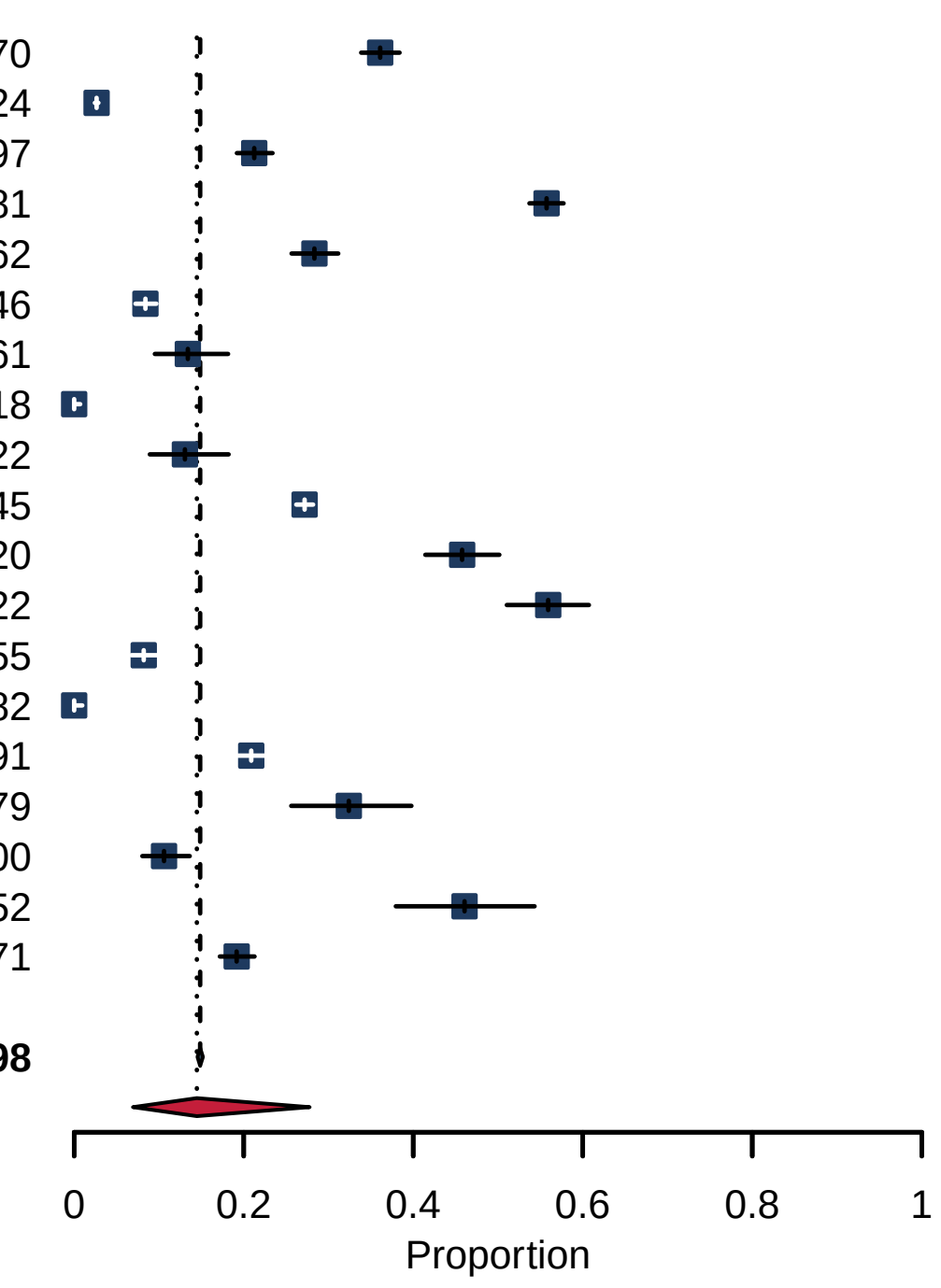

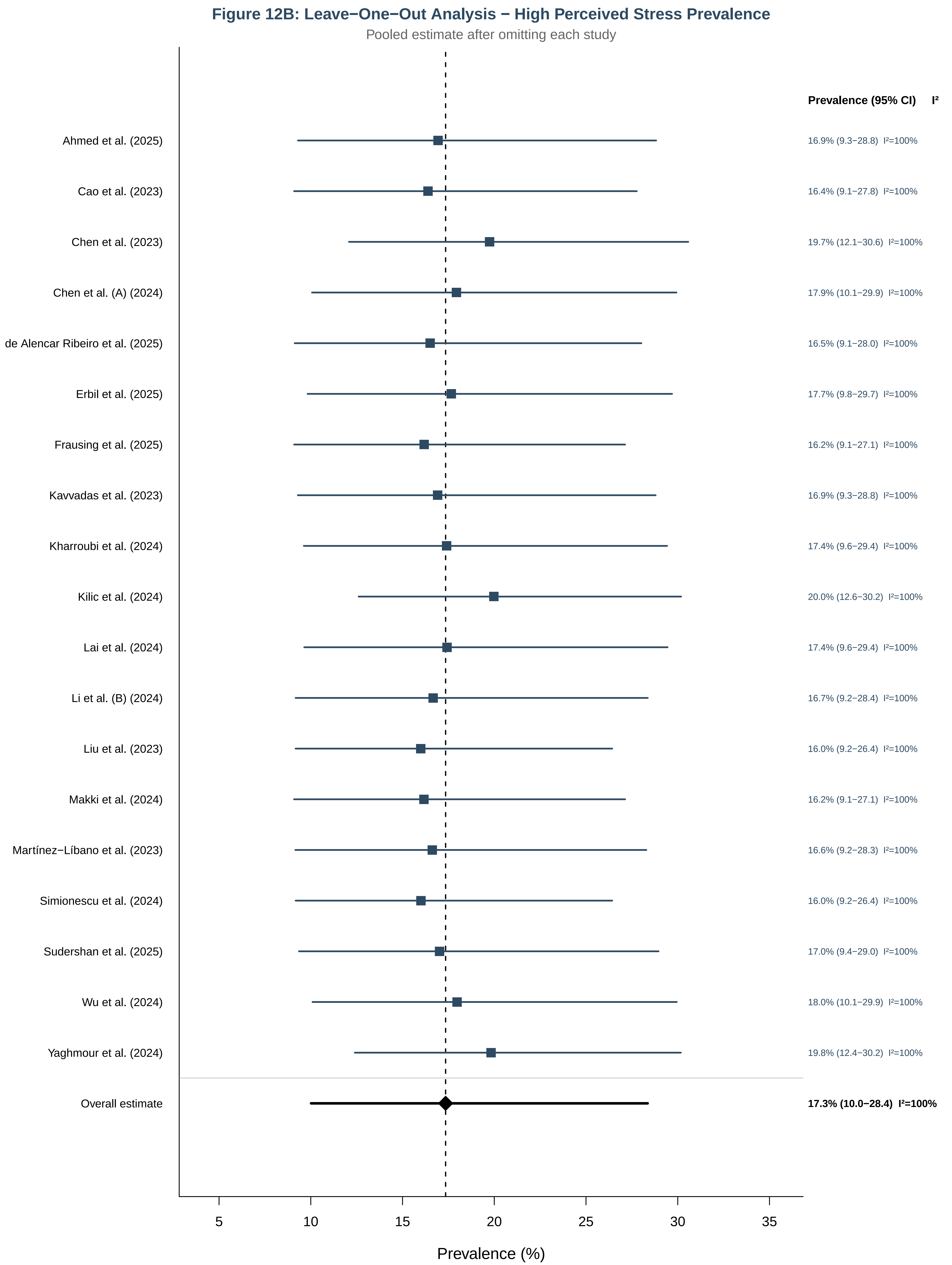
Figure 12B: Leave−One−Out Analysis − High Perceived Stress Prevalence
Pooled estimate after omitting each study
Prevalence (95% CI) I²
Ahmed et al. (2025) 16.9% (9.3−28.8) I²=100%
Cao et al. (2023) 16.4% (9.1−27.8) I²=100%
Chen et al. (2023) 19.7% (12.1−30.6) I²=100%
Chen et al. (A) (2024) 17.9% (10.1−29.9) I²=100%
de Alencar Ribeiro et al. (2025) 16.5% (9.1−28.0) I²=100%
Erbil et al. (2025) 17.7% (9.8−29.7) I²=100%
Frausing et al. (2025) 16.2% (9.1−27.1) I²=100%
Kavvadas et al. (2023) 16.9% (9.3−28.8) I²=100%
Kharroubi et al. (2024) 17.4% (9.6−29.4) I²=100%
Kilic et al. (2024) 20.0% (12.6−30.2) I²=100%
Lai et al. (2024) 17.4% (9.6−29.4) I²=100%
Li et al. (B) (2024) 16.7% (9.2−28.4) I²=100%
Liu et al. (2023) 16.0% (9.2−26.4) I²=100%
Makki et al. (2024) 16.2% (9.1−27.1) I²=100%
Martínez−Líbano et al. (2023) 16.6% (9.2−28.3) I²=100%
Simionescu et al. (2024) 16.0% (9.2−26.4) I²=100%
Sudershan et al. (2025) 17.0% (9.4−29.0) I²=100%
Wu et al. (2024) 18.0% (10.1−29.9) I²=100%
Yaghmour et al. (2024) 19.8% (12.4−30.2) I²=100%
Overall estimate 17.3% (10.0−28.4) I²=100%
5
10
15
20
25
30
35
Prevalence (%)

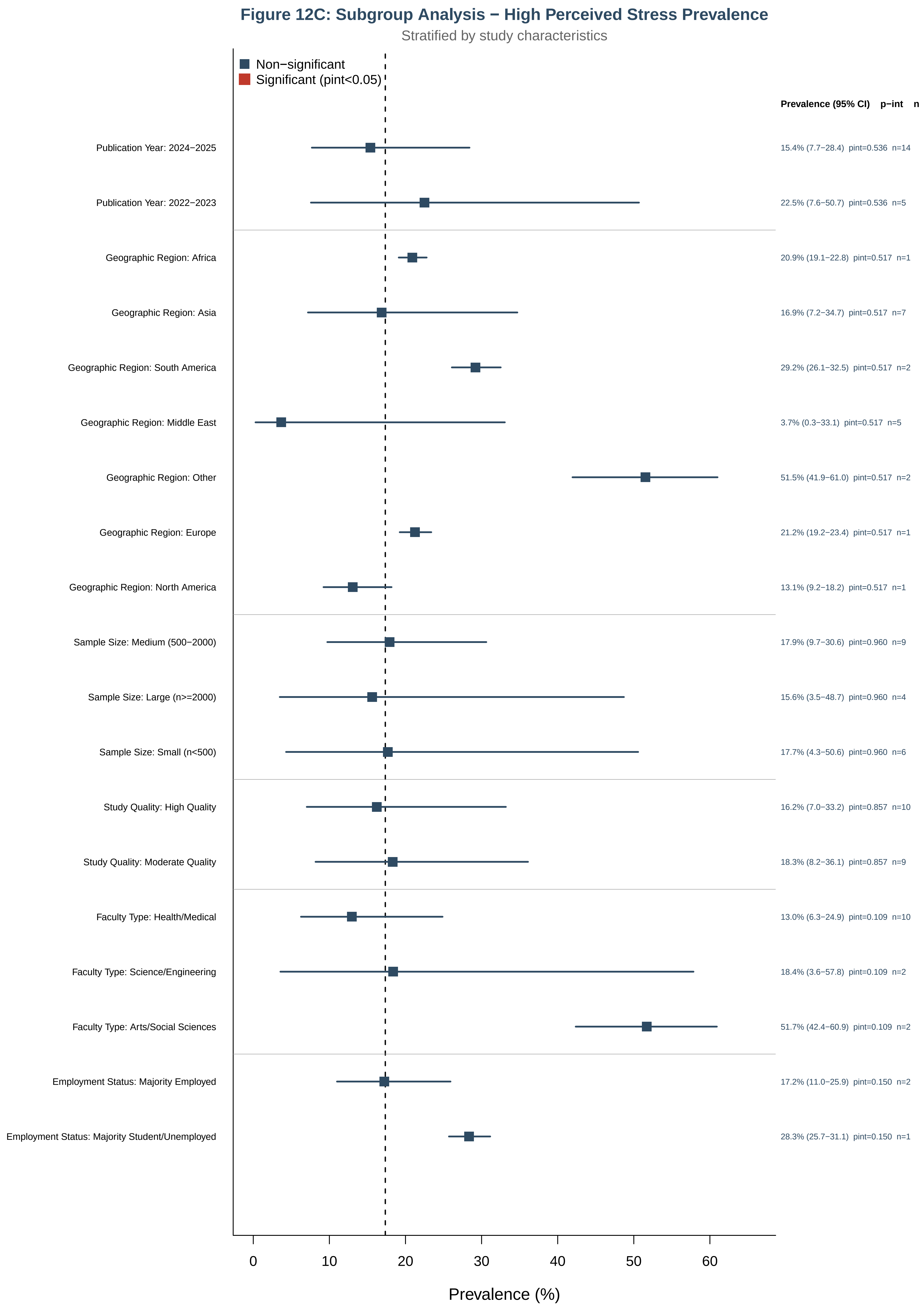
Figure 12C: Subgroup Analysis − High Perceived Stress Prevalence
Stratified by study characteristics
Non−significant
Significant (pint<0.05)
Prevalence (95% CI) p−int n
Publication Year: 2024−2025
15.4% (7.7−28.4) pint=0.536 n=14
Publication Year: 2022−2023
22.5% (7.6−50.7) pint=0.536 n=5
Geographic Region: Africa
20.9% (19.1−22.8) pint=0.517 n=1
Geographic Region: Asia
16.9% (7.2−34.7) pint=0.517 n=7
Geographic Region: South America
29.2% (26.1−32.5) pint=0.517 n=2
Geographic Region: Middle East
3.7% (0.3−33.1) pint=0.517 n=5
Geographic Region: Other
51.5% (41.9−61.0) pint=0.517 n=2
Geographic Region: Europe
21.2% (19.2−23.4) pint=0.517 n=1
Geographic Region: North America
13.1% (9.2−18.2) pint=0.517 n=1
Sample Size: Medium (500−2000)
17.9% (9.7−30.6) pint=0.960 n=9
Sample Size: Large (n>=2000)
15.6% (3.5−48.7) pint=0.960 n=4
Sample Size: Small (n<500)
17.7% (4.3−50.6) pint=0.960 n=6
Study Quality: High Quality
16.2% (7.0−33.2) pint=0.857 n=10
Study Quality: Moderate Quality
18.3% (8.2−36.1) pint=0.857 n=9
Faculty Type: Health/Medical
13.0% (6.3−24.9) pint=0.109 n=10
Faculty Type: Science/Engineering
18.4% (3.6−57.8) pint=0.109 n=2
Faculty Type: Arts/Social Sciences
51.7% (42.4−60.9) pint=0.109 n=2
Employment Status: Majority Employed
17.2% (11.0−25.9) pint=0.150 n=2
Employment Status: Majority Student/Unemployed
28.3% (25.7−31.1) pint=0.150 n=1
0
10
20
30
40
50
60
Prevalence (%)

Figure 12D: Meta−Regression Analysis

High Perceived Stress Prevalence

Effect of baseline covariates on prevalence (univariate analysis)

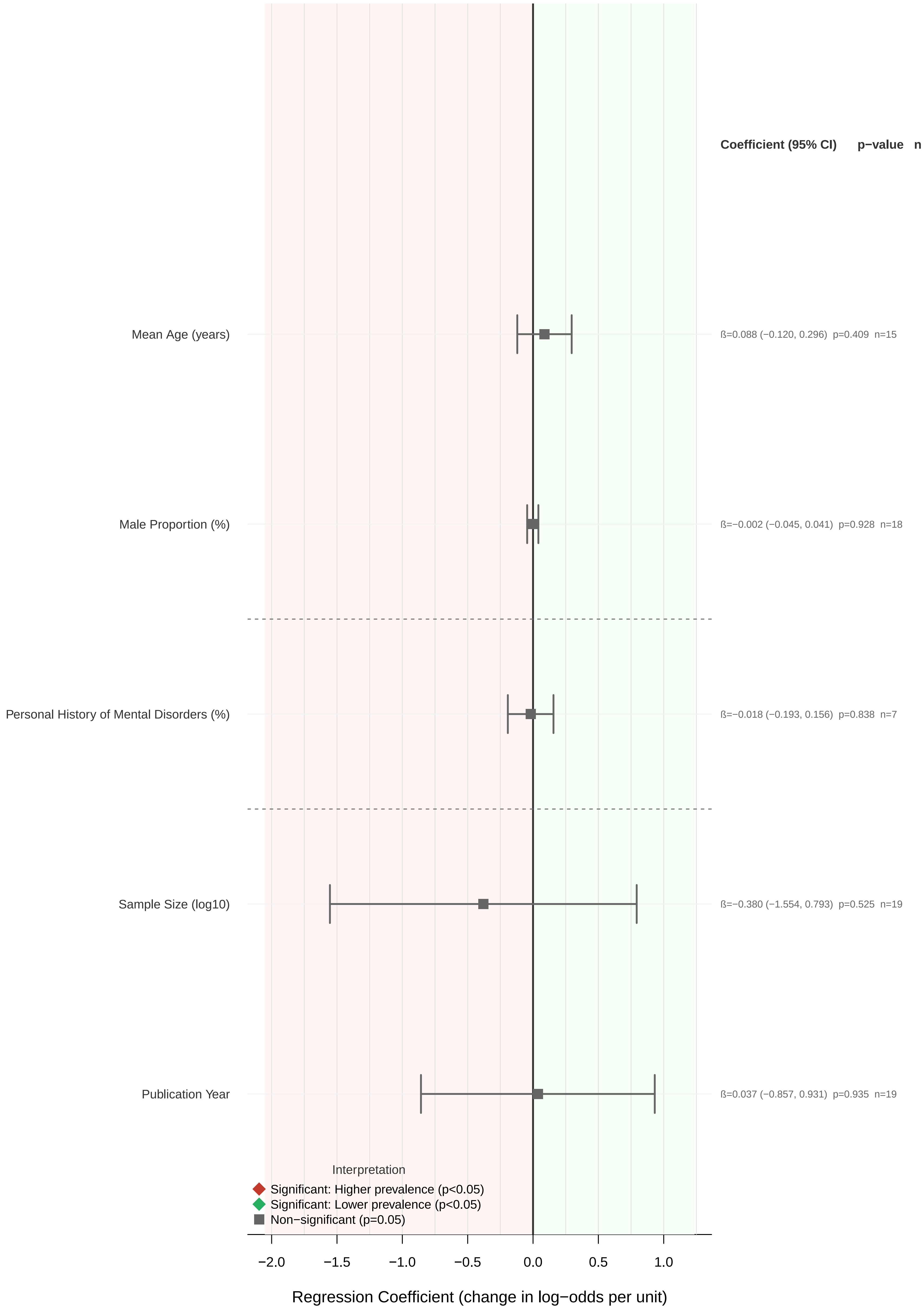

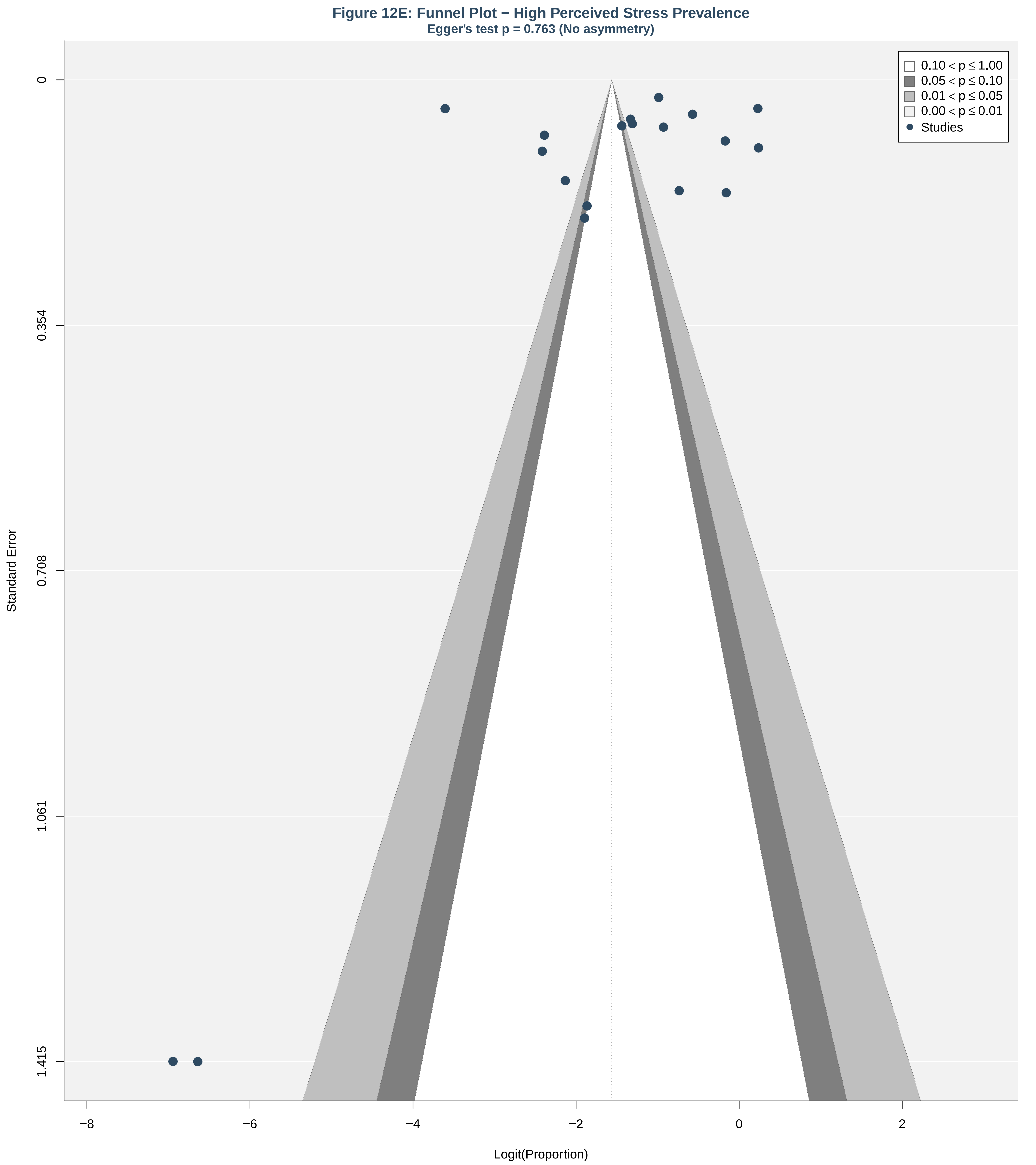

Figure 12E: Funnel Plot − High Perceived Stress Prevalence
Egger's test p = 0.763 (No asymmetry)
0.10 < p ≤ 1.00
0.05 < p ≤ 0.10
0.01 < p ≤ 0.05
0.00 < p ≤ 0.01
Studies
Standard Error
0
0.354
0.708
1.061
1.415
−8
−6
−4
−2
0
2
Logit(Proportion)

ANALYSIS COMPLETE

Single−Arm Meta−Analysis of Proportions

Outcomes Analyzed:

Depression Prevalence | Anxiety Prevalence | Stress Prevalence

Mild/Moderate/Severe Depression | Mild/Moderate/Severe Anxiety | High Stress

For Each Outcome:

A: Forest Plot (Common + Random Effects, Pooled Proportion)

B: Leave−One−Out Sensitivity Analysis

C: Subgroup Analysis (Region, Year, Sample Size, Tools)

D: Meta−Regression (Year, Sample Size)

E: Funnel Plot with Egger's Test

Total Studies: ~63 | Total Participants: N = 216,060
